# Probing the Free Energy of Small Water Clusters: Revisiting Classical Nucleation Theory

**DOI:** 10.1021/acs.jpclett.2c01361

**Published:** 2022-08-22

**Authors:** Ali Afzalifar, George C. Shields, Vance R. Fowler, Robin H. A. Ras

**Affiliations:** † Department of Applied Physics, Aalto University School of Science, Puumiehenkuja 2, 02150 Espoo, P.O. Box 15100, Aalto FI-00076, Finland; ‡ Department of Chemistry, Furman University, Greenville, South Carolina 29613, United States; § Department of Bioproducts and Biosystems, Aalto University School of Chemical Engineering, P.O. Box 16000, Aalto FI-00076, Finland

## Abstract

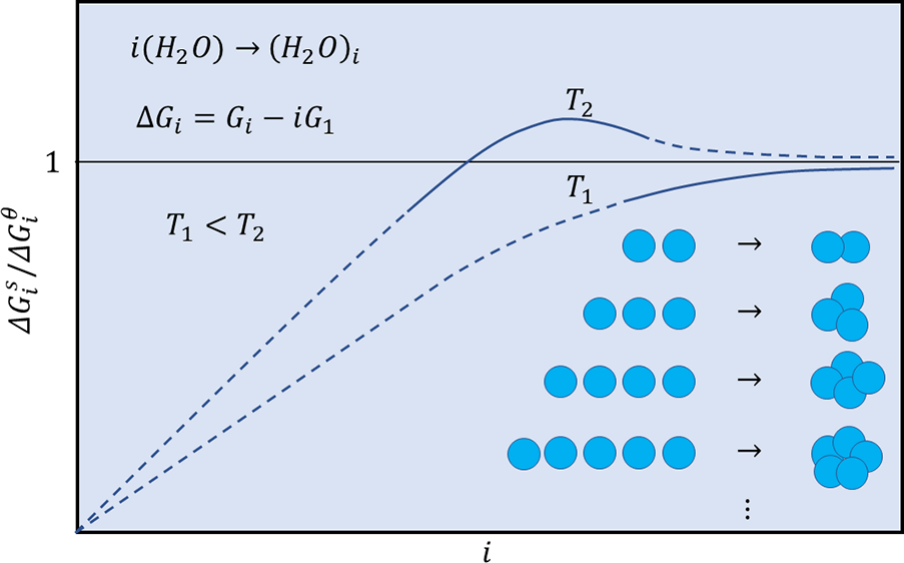

By addressing the defects in classical nucleation theory (CNT), we develop an approach for extracting the free energy of small water clusters from nucleation rate experiments without any assumptions about the form of the cluster free energy. For temperatures higher than ∼250 K, the extracted free energies from experimental data points indicate that their ratio to the free energies predicted by CNT exhibits nonmonotonic behavior as the cluster size changes. We show that this ratio increases from almost zero for monomers and passes through (at least) one maximum before approaching one for large clusters. For temperatures lower than ∼250 K, the behavior of the ratio between extracted energies and CNT’s prediction changes; it increases with cluster size, but it remains below one for almost all of the experimental data points. We also applied a state-of-the-art quantum mechanics model to calculate free energies of water clusters (2–14 molecules); the results support the observed change in behavior based on temperature, albeit for temperatures above and below ∼298 K. We compared two different model chemistries, DLPNO-CCSD(T)/CBS//ωB97xD/6-31++G** and G3, against each other and the experimental value for formation of the water dimer.

Water is essential to life and the most abundant substance on the earth’s surface, and thus understandably the most extensively studied substance in the history of science. However, the more we sharpen our theoretical and experimental tools, the more elusive the behavior of water seems, which leaves it as an enduring mystery.^1^ The lack of knowledge about the anomalous properties of water and its structure is still one of the big unsolved problems in science.^2^ In particular, the accurate representation of the energetics of small water clusters has been an attractive topic due to its importance for the development of liquid and gas state models of water and also application in various fields, e.g., atmospheric science, nanotechnology, and the energy industry. In this Letter, we propose an approach for calculating the free energy of water clusters, from the experiments on water nucleation rate, without any assumptions about the form of the cluster free energy.

To do so, we use classical nucleation theory (CNT), which still provides the most popular framework for predicting nucleation, despite decades of research after its development by Becker and Döring^3^ and Zeldovich.^4^ The popularity of CNT stems from its simplicity, achieved by controversial assumptions about the free energy of cluster formation, which renders its prediction questionable and in many cases in obvious disagreement with experiment. Therefore, we begin developing our approach by addressing CNT’s problems and some common but inaccurate views about CNT. It should be noted that although here the focus is on water, this approach can be used for other substances, as well. Using this approach, we probe the water nucleation experiments and show that at higher temperatures (∼250 K < *T*) the ratio of the extracted free energy to CNT’s prediction exhibits nonmonotonic behavior with a change in cluster size: toward the smallest clusters, this ratio continuously decreases to almost zero for monomers, while as the cluster size increases, this ratio surpasses one and has at least one maximum before returning toward one for sufficiently large clusters. For lower temperatures (*T* < ∼250 K), this ratio stays below one for almost all experimental data, although it increases with cluster size.

The kinetic aspect of CNT combined with its thermodynamic aspect treats nucleation in the form of a transition from the metastable vapor to the liquid phase as clusters surmount a free energy barrier. The rate of this transition is called the nucleation rate and (applying Courtney’s correction^5^) is
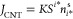
1where *K* is the product of the Zeldovich factor^4^ and the frequency of attachment of the monomer to the critical cluster, i* is the number of molecules in the critical cluster, *S* is the supersaturation [the ratio of vapor pressure to saturation pressure (*S* = *P*_v_/*P*_s_)], and *n*_i*_^s^ is the number density of critical clusters in the saturated vapor. The term *S*^i*^ formulates the thermodynamic driving force for the phase transition from a supersaturated vapor to a liquid cluster of size i*. For an arbitrary cluster size, the thermodynamic driving force is readily calculated as *S* is experimentally measurable. In contrast to *S*^i*^, CNT resorts to several assumptions to calculate *n*_*i*_^s^. Following Boltzmann’s law, *n*_*i*_^s^ is related to the cluster free energy at saturation *ΔG*_*i*_^s^
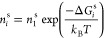
2where *k*_B_ is Boltzmann’s constant and *T* is the temperature. CNT presupposes that *ΔG*_*i*_^s^ is solely equal to surface work *ΔG*_*i*_^θ^ and omits theoretical possibilities such as contributions of configurational effects and various degrees of freedom to the cluster free energy. CNT considers an *i*-mer as a spherical droplet with a sharp interface and properties identical to those of the bulk liquid. Thus, the surface work is calculated as *ΔG*_*i*_^θ^ = θ_∞_*A*_1_*i*^2/3^, the product of planar surface tension θ_∞_ and cluster surface area *A*_1_*i*^2/3^, where *A*_1_ is the monomer surface area. One naturally expects that for monomers eq 2 satisfies the identity *n*_1_^s^ = *n*_1_^s^. This requires that *ΔG*_1_^θ^ = 0, which is not true in CNT; hence, CNT appears to be self-inconsistent. Girshick and Chiu^6^ suggested a simple way to recover self-consistency [termed the internally consistent classical theory (ICCT)] by correcting the surface work with the equation *ΔG*_*i*_^θ^ = *θA*_1_(*i*^2/3^ – 1). The requirement that *ΔG*_1_^θ^ = 0 has been questioned stating that CNT treats monomers as single-molecule droplets rather than vapor monomers.^7,8^ However, Saltz^9^ argued that (except for the lack of the 1/*S* factor) the theory is consistent because, on the basis of the ideal vapor assumption in CNT, *n*_1_^s^ is equal to total cluster number density *n*^s^. Although Saltz’s argument is correct in a strict sense, it is intriguing to conceive this problem conversely. We can drop the ideal vapor assumption but think of *n*^s^ just as an approximation for *n*_1_^s^. Therefore, setting *n*_1_^s^ ≈ *n*^s^ = *P*_s_/*k*_B_*T*, eq 2 must yield *ΔG*_1_^θ^ = 0, which is also an approximation. We discuss below how this approximation improves the prediction of free energy for monomers and dimers.

Upon determination of the free energy barrier to nucleation as *ΔG*_*i*_ = *ΔG*_*i*_^s^ – (*i* – 1)*k*_B_*T* ln(*S*), i* is found through Gibbs free energy minimization, resulting in the so-called Gibbs–Thomson equation
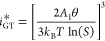
3

The accuracy of the Gibbs–Thomson equation can be checked against the first nucleation theorem,^10^ which provides a model-independent way to calculate i* from the nucleation rate as
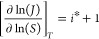
4where for an experimental nucleation rate *J*_exp_, the result is called the experimental critical size *i*_exp_^*^. Using eq 4, Girshick^11^ showed the Gibbs–Thomson equation disagrees with the experiments for water and several other substances. The comparison between *i*_GT_^*^ and *i*_exp_^*^ for nine nucleation experiments with water is shown in Figure 1a. Girshick related the deviation between *i*_GT_^*^ and *i*_exp_^*^ to a size-dependent error in calculating *ΔG*_*i*_^θ^. Merikanto et al.^12^ also showed, through Monte Carlo simulations, that below a certain size threshold the error in *ΔG*_*i*_^θ^ is size-dependent. Both works stated that the stepwise free energy change upon addition of the monomer, ΔΔ*G*_*i*_^θ^ = Δ*G*_*i*_^θ^ – Δ*G*_*i*–1_^θ^, is expected to approach the macroscopic prediction as the size of the droplet increases, not the total surface work. As *i* increases, the error in ΔΔ*G*_*i*_^θ^ decreases to zero, and the total error in *ΔG*_*i*_^θ^ becomes independent of size and dependent on only temperature; i.e., after a size threshold, the actual cluster free energy can be calculated as θ_∞_*A*_1_*i*^2/3^ – *D*(*T*), where *D*(*T*) is a correction term. Consequently, if i* becomes larger than the size threshold, the first nucleation theorem dictates that *i*_GT_^*^ = *i*_exp_^*^. Therefore, a correction that depends on only temperature, as proposed by McGraw and Laaksonen,^13^ inevitably relies on the validity of the Gibbs–Thomson equation or assumes that *i*_exp_^*^ is larger than the threshold. However, the reported experimental critical sizes are in the subnanometer range, and it is unlikely that they surpassed the relevant threshold. Indeed, the deviation of *i*_GT_^*^ from *i*_exp_^*^ in Figure 1a shows that the size threshold is not surpassed by *i*_exp_^*^ in most of the experiments.

**Figure 1:**
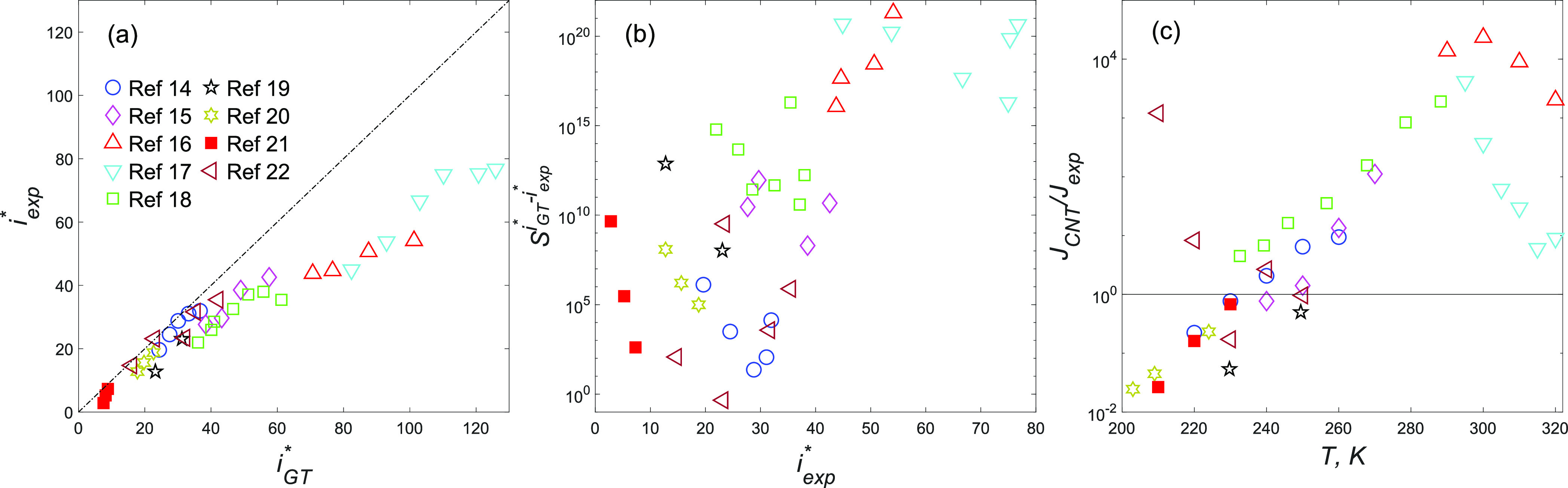
(a) Comparison of *i*_exp_^*^ with *i*_GT_^*^ from refs (14−22). Values for refs (14−17), (20), and (21) are from the original references. Those for refs (18) and (19) are from ref (15). Those for ref (22) are calculated in this work. All *i*_exp_^*^ values are calculated including the number 1 on the right-hand side of eq 4. (b) *S*^*i*_GT_^*^–*i*_exp_^*^^ vs *i*_exp_^*^. (c) *J*_CNT_/*J*_exp_ vs *T*. Values of *i*_GT_^*^, *J*_CNT_, and *J*_exp_ correspond to the supersaturation at which *i*_exp_^*^ is calculated.

The potential effect of the deviation between *i*_GT_^*^ and *i*_exp_^*^ on nucleation rate is illustrated by means of *S*^*i*_GT_^*^–*i*_exp_^*^^ in Figure 1b. It is noted that we exclude the first two isotherms of ref (22) from the analysis as their measured rates significantly deviate from the others. In all cases, correcting *S*^i*^ in eq 1 would decrease the nucleation rate, particularly toward higher isotherms and/or larger critical clusters. Although this effect is an obvious consequence of the first nucleation theorem, it is concealed by the typical ways of comparing CNT with experiment in the literature, such as Figure 1c, which shows *J*_CNT_/*J*_exp_ versus temperature. The deviations in *J*_CNT_/*J*_exp_ are too small to be indicative of the huge errors associated with *S*^*i*_GT_^*^–*i*_exp_^*^^. The error caused by overprediction of i* is compensated by the exponential decrease in *n*_i_^*s^ due to the increase in Δ*G*_*i*_^θ^. This compensation mechanism in CNT along with the oversimplified comparisons between CNT and experiment may be the reason that the significance of the inaccuracy of the Gibbs–Thomson equation has not been previously identified. Moreover, these comparisons have been partly responsible for echoing the view that CNT exhibits a strong temperature dependence without considering the other variables (e.g., see refs (15), (20), (23), and (24)). For instance, it appears in Figure 1c that CNT underpredicts and overpredicts the nucleation rates for temperatures below and above ∼250 K, respectively. On the basis of this view, Wölk and Strey^14^ proposed an empirical correction to *J*_CNT_ in the form of exp(*A* + *B*/*T*), where *A* and *B* are constants. This correction was later claimed^25^ to be the experimental substantiation of the function *D*(*T*) envisaged by McGraw and Laaksonen.^13^ However, a similar correction can be easily assumed in the form of exp(*A*′ + *B*′*T*)/*S*, which not only is equally successful in improving CNT’s prediction but also includes the 1/*S* factor, which as explained in ref (26) brings CNT in line with both the law of mass action and the first nucleation theorem (see Figure S1). This accidental success requires restraint before attaching a physical significance or insight to such corrections. We do not dismiss the role of a wrong temperature dependence in CNT’s failure but stress that each variable needs to be studied in isolation. Here, we aim to include the cluster size effect in our examination for two reasons. First, the deviation of *i*_GT_^*^ from *i*_exp_^*^ indicates that the error in nucleation barriers in CNT is dependent on size. Second, following the recommendation of Gibbs^27^ and Tolman,^28^ we consider the curvature/size dependence of surface tension. It is noted that, in the case of microscopic cluster sizes, the surface tension is reduced from a physical property to a mathematical quantity.

Questioning the validity of Gibbs–Thomson equation opens a new pathway for evaluating CNT in terms of the cluster size. This path requires an approach, immune from CNT’s assumptions in calculating *ΔG*_*i*_^θ^, for extracting the cluster free energy from experiments. We employ eq 1 to probe experiments and solve this equation for *ΔG*_*i*_^s^. Because *S*, *i*_exp_^*^, and *J*_exp_ are known, only Zeldovich factor *Z* and monomer attachment frequency *C*_*i*_ are needed to solve eq 1. For *C*_*i*_, the standard formulation^29^ reads
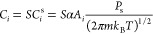
5where *C*_*i*_^s^ is the monomer attachment frequency at saturation pressure, α is the sticking probability coefficient, *A*_*i*_ is the cluster surface area, and *m* is the molecular mass. When *i* is treated as a continuous variable, the Zeldovich factor is given as .^4^ As Δ*G*(*i*) is unknown, we do not resort to the thermodynamics of nucleation to calculate *Z* and obtain this factor kinetically without any assumption about the form of Δ*G*(*i*). In a saturated vapor, the principle of detailed balance dictates
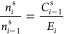
6where *E*_*i*_ is the frequency of detachment of the monomer from an *i*-mer. Using eq 6, *n*_*i*_^s^ is defined in a recursive fashion
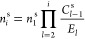
7

Considering *l* as a continuous variable and using eq 2, eq 7 is represented as
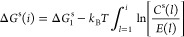
8

Taking derivatives from both sides of eq 8 with respect to *i* at *i* = i* yields *S* = *E*(i*)/*C*^s^(i*), where *S* defines the supersaturation under a fictitious equilibrium condition, where i* would amount to the critical size. We emphasize that *S* = *E*(i*)/*C*^s^(i*) derived here in a saturated vapor is equivalent to *E*(i*) = *C*(i*) for a supersaturated vapor. The latter as Kashchiev^30^ stated is the kinetic definition of the critical cluster and was determined previously (see refs (31) and (32)). By setting *E*(i*) = *C*^s^(i*)*S* and using eq 5, the right-hand side of eq 6 is recast as
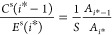
9

Moreover, Δ*G*^s^(i* – 1) is expanded as a Taylor series about i*
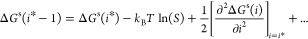
10

Truncating the above series after the second-order term and using eq 2, the left-hand side of eq 6 is approximated as
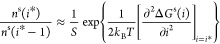
11

Rewriting both sides of eq 6 by using eqs 9 and 11 leads to [∂^2^Δ*G*(*i*)/*∂i*^2^]_*i*=i*_ ≈ −2*k*_B_*T* ln(*A*_i*_/*A*_i*–1_), which when inserted into the relation for *Z* provides an approximation for this factor as
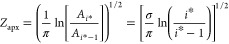
12where the second equality is obtained by recalling that in CNT *A*_i*_ = *A*_1_i^*σ^ (σ = ^2^/_3_). Finally, according to eq 1, we can deduce Δ*G*^s^(*i*) from the experiment as
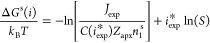
13

The calculation of *Z* by eq 12 is nearly independent of the model, and to the best of our knowledge, it is the first expression of the Zeldovich factor from the kinetic standpoint. This is because i* is given by the first nucleation theorem, which is model independent, and eq 12 is derived depending on just *C*(*i*) ∝ *A*_*i*_ ∝ *i*^*σ*^. Although the first proportionality is reasonable, the second one with σ = ^2^/_3_ invokes the spherical assumption about the cluster shape. This assumption was shown to be inaccurate in the molecular dynamics simulations of simple fluids, resulting in nonspherical shapes and consequently larger surface areas for clusters as large as a 100-mer.^33,34^ Also, ref (35) proposed a temperature dependency for σ changing its value a few percent from ^2^/_3_. Nonetheless, Δ*G*^s^(*i*) given by eq 13 is not sensitive to σ; e.g., setting σ = ^3^/_4_ increases Δ*G*^s^(*i*) by only 0.06 *k*_B_*T*. The important point is eq 13 requires no information about Δ*G*^s^(*i*) and thus provides an independent check on Δ*G*^*θ*^(*i*) in CNT.

Before discussing Δ*G*^s^(*i*) obtained from experiments, let us conduct a thought experiment to reveal the behavior of Δ*G*^s^(*i*)/Δ*G*^*θ*^(*i*) versus *i*. We expect that by increasing *i* and surpassing the size threshold one can calculate the actual cluster free energy as Δ*G*^s^(*i*) = Δ*G*^*θ*^(*i*) – *D*(*T*), which leads to the asymptotic behavior . We impose two conditions to refine the scenarios under which  can approach unity. The first condition requires that Δ*G*_1_^s^ must be close to zero because at temperatures below the critical point the vapor is mainly monomers. The second condition relies on the average free energy of dimers calculated from the second virial coefficient in the water vapor virial equation of state. Below the critical point where the cluster–cluster interactions are insignificant, direct relations can be established between the number density of the smallest clusters in the vapor and the coefficients in the virial series. To calculate *n*_2_^s^, we follow the relations developed by Saltz^9^ that can also be observed in Monte Carlo calculations^36^ (see ref (37) for a similar application of these relations). Knowing *n*_2_^s^, we calculated the free energy of dimers using eq 2 and replacing *n*_1_^s^ with *n*^s^ (see the related discussion in the Supporting Information). For temperatures from 200 to 375 K, the experimental^38^ and correlational^39−42^ values of the second virial coefficient suggest that Δ*G*_2_^s^/Δ*G*_2_^θ^ is much less than one (∼0.5) (see Figure 2). The first and second conditions together state that in any scenario Δ*G*^s^(*i*)/Δ*G*^*θ*^(*i*) increases from ∼0 at *i* = 1 to ∼0.5 at *i* = 2. Therefore, one can envisage two general scenarios under which Δ*G*^s^(*i*)/Δ*G*^*θ*^(*i*) starts by an increase between *i* = 1 and *i* = 2 and then approaches one. In the first scenario, Δ*G*^s^(*i*)/Δ*G*^*θ*^(*i*) crosses unity at least once and manifests at least one maximum point, while in the second scenario, Δ*G*^s^(*i*) always remains below Δ*G*^*θ*^(*i*) and Δ*G*^s^(*i*)/Δ*G*^*θ*^(*i*) may have zero to an infinite number of extrema. In the simplest case fulfilling the first scenario, Δ*G*^s^(*i*)/Δ*G*^*θ*^(*i*) exhibits a single maximum after crossing unity and before surpassing the size threshold. The simplest case fulfilling the second scenario is a curve monotonically increasing from ∼0 and approaching unity from below, similar to ICCT.

**Figure 2:**
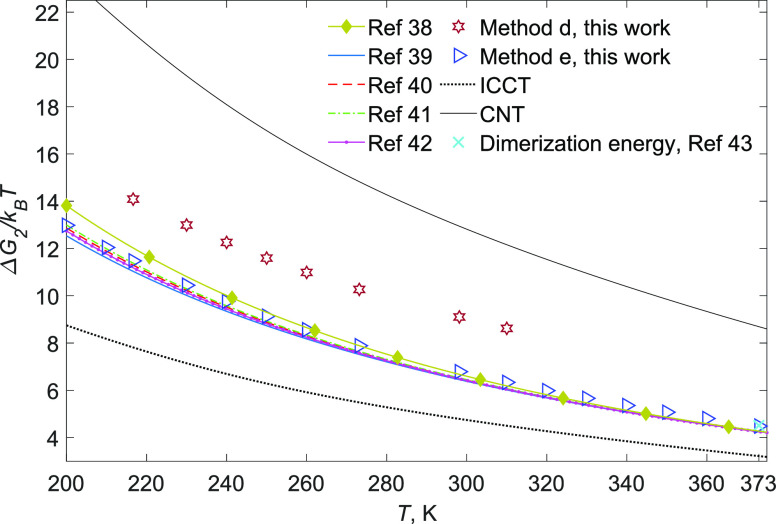
*ΔG*_2_^s^ as a function of temperature from the second virial coefficients,^38−42^ experimental water dimerization free energy at 373 K,^43^ the simulation results from methods d and e of this work, and predictions by CNT and ICCT.

With regard to the aforementioned conditions, in comparison to CNT, ICCT predicts the free energies of monomers and dimers closer to those obtained on the basis of monomer concentration and the second virial coefficient. Figure 2 indicates that all of the data of the second virial coefficient of water, and the experimental water dimerization free energy along with the quantum mechanical simulations for Δ*G*_2_^s^ (which are discussed below), show that CNT, in contrast to ICCT, greatly overpredicts the free energy of dimers. It is noted that near the triple point the vapor compressibility factor is quite close to one and the contributions of the higher-order virial coefficients to vapor pressure become relatively unnoticeable. Thus, the data on the second virial coefficient for the temperature range of interest in nucleation experiments (210–320 K) either are not available or cover only the higher end of the temperature range. As a remedy to this problem, the relations for the second virial coefficient are extrapolated down to 200 K in Figure 2; these relations were claimed to be valid down to 273.15 K. It is acknowledged that by extrapolation toward lower temperatures the accuracy of these relations deteriorates. However, all of these relations are in close agreement with one another, and they, along with simulation results, clearly lie below CNT throughout the extrapolated temperature range. This agreement restores our confidence that these relations are accurate enough to show that CNT overpredicts the free energy of dimers. In other words, Δ*G*_2_^s^/Δ*G*_2_^θ^ is evidently smaller than one.

The Δ*G*^s^(*i*) from eq 13 is compared with Δ*G*^*θ*^(*i*) by CNT versus *i* and *T* in panels a and b, respectively, of Figure 3. Although a formal uncertainty analysis is inapplicable for the obtained Δ*G*^s^(*i*), because it is derived by combining modeling and fitting to the different data sets, we attempt to provide reasonable estimate envelopes to consider the uncertainties. The error bars in panels a and b of Figure 3 correspond to the uncertainty in *i*_exp_^*^ and the estimate envelopes for Δ*G*^s^(*i*). To calculate the envelope bounds, the uncertainties in *i*_exp_^*^ and *C*(*i*) are considered conservatively to achieve the largest bounds. For *i*_exp_^*^, from the reported uncertainties in refs (14) and (20−22) the largest is selected (±15% from ref (21)). Due to the direct relation between *J*_exp_ and *i*_exp_^*^, the large uncertainty considered for *i*_exp_^*^ should incorporate the uncertainties into *J*_exp_. For *C*(*i*), the main source of uncertainty is the sticking probability coefficient. Historically, a broad range of values (0.01–1.0) for α were observed in experiments.^44^ However, recent reviews^45−47^ and experiments^48−50^ reported larger values for α (0.2–1.0). In particular, ref (51) showed that, by taking into account the inaccuracies in the thermophysical and experimental parameters, α should be >0.5. Moreover, it has been argued that *C*(*i*) is underestimated by eq 5 by a factor of ≤2 due to the neglect of the attractive potential between the droplet and monomers.^52,53^ Therefore, we assume *C*(*i*) may deviate from eq 5 with α = 1 by a factor of 0.5–2.

**Figure 3:**
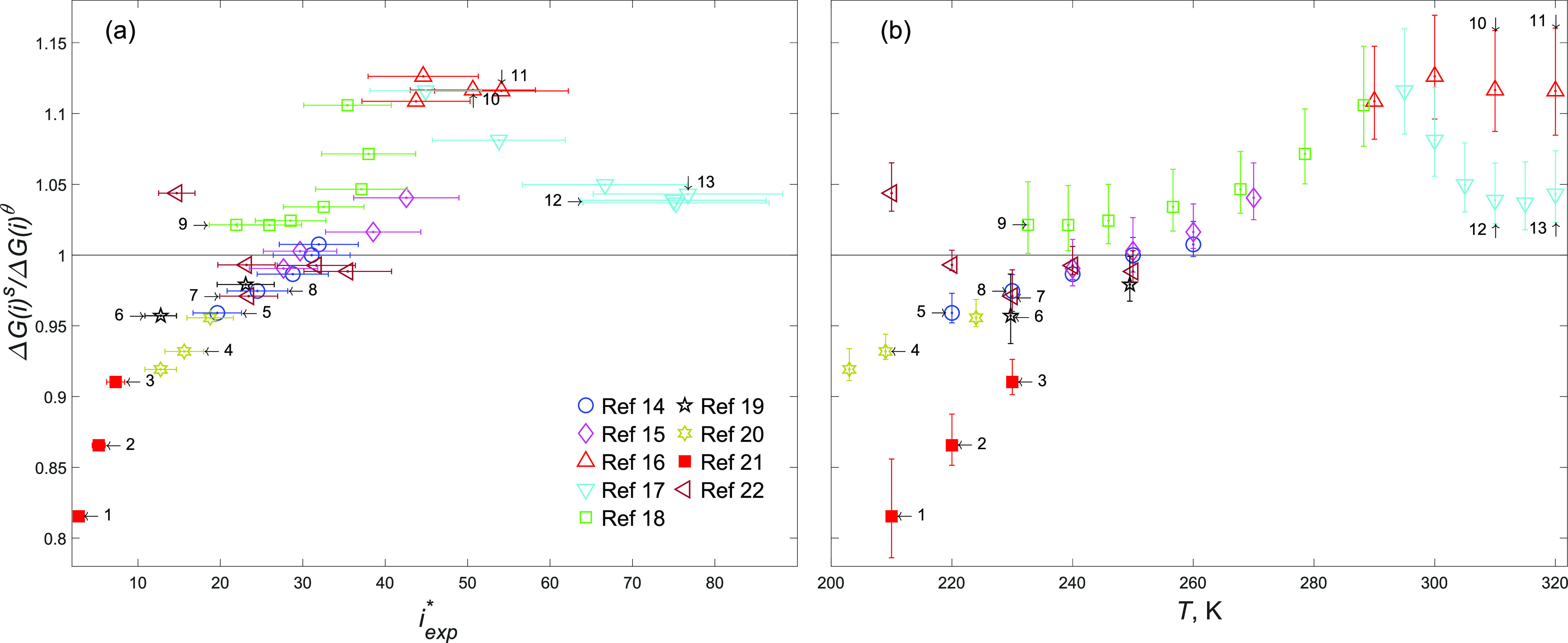
Comparison of Δ*G*^s^(*i*) from eq 13 with Δ*G*^*θ*^(*i*) from CNT vs (a) *i*_exp_^*^ and (b) temperature (both panels share the same vertical axis).

Even upon consideration of these uncertainties, many data points lie above one in Figure 3b, which is clearly indicative of the first scenario, because a crossover (from below one to above one) must have happened before *i*_exp_^*^. The points below one all belong to the smaller clusters observed at temperatures lower than ∼250 K. Leaving aside temperature, for these points as *i*_exp_^*^ increases Δ*G*^s^(*i*)/Δ*G*^*θ*^(*i*) also increases. This pattern is also observed for the points on similar isotherms of 210, 220, and ∼230 K whose *i*_exp_^*^ and Δ*G*^s^(*i*) error bars do not overlap. Comparing point 1 with point 4, point 2 with point 5, point 3 with points 6–8, and point 6 with point 9 shows the increase in Δ*G*^s^(*i*)/Δ*G*^*θ*^(*i*) on the aforementioned isotherms as the cluster size increases. The fact that Δ*G*^s^(*i*)/Δ*G*^*θ*^(*i*) always remains below one suggests that the second scenario may be true in the case of the lower temperatures. However, due to the lack of data for larger clusters, the existence of a crossover cannot be completely dismissed at these temperatures. For instance, we may conjecture that a crossover occurs between points 6 and 9, which both approximately are at 230 K. Interestingly, the opposite pattern is observed for the data points above one. Comparing point 10 with point 12 (310 K) and point 11 with point 13 (320 K) reveals that as the size increases, Δ*G*^s^(*i*)/Δ*G*^*θ*^(*i*) decreases; recalling the first scenario, this suggests that these points are located after a maximum.

Moreover, we are interested in cross-checking our observation of the behavior of water cluster energies with the help of computational chemistry. It is acknowledged that simulations based on classical mechanics can provide rigorous approaches for dealing with vapor nucleation and assessment of CNT (see, for example, refs (56) and (57)). However, motivated by findings in previous quantum mechanical simulations by Dunn et al.^54^ and Du et al.^55^ (which showed the nonmonotonic behavior of ΔΔ*G*_*i*_ for *i* = 2–6 and *i* = 2–10, respectively), we also apply state-of-the-art quantum mechanics models to investigate the free energy of water clusters. After extensive conformational searching using Ogolem,^58^ geometry optimizations and frequency calculations were computed with the ωb97xd density functional^59^ and electronic energies were corrected with the DLPNO-CCSD(T) method using the cc-pVNZ basis sets.^60,61^ Single-point calculations with N = D, T, and Q were extrapolated to the complete basis set (CBS) limit and used to determine Gibbs free energies.^62−64^ We determined the DLPNO-CCSD(T)/CBS//ωB97xD/6-31++G** average free energy change in cluster formation from monomers to clusters, *i*H_2_O > (H_2_O)_*i*_, using the lowest-Gibbs free energy (H_2_O)_*i*_ clusters for eight different temperatures ranging from 216.65 to 310 K. In addition to determining the overall Δ*G*° values for cluster formation, we calculated the stepwise monomer addition (ΔΔ*G*°) for each successive addition of a water monomer to the proceeding cluster for *i* = 2–10 [H_2_O + (H_2_O)_*i*−1_ > (H_2_O)_*i*_]. We note that the minimum energy structures change as a function of temperature for *i* = 4, 6, 8, 9, and 10 and that using the lowest-energy structure instead of Botzmann averaging the ensemble of low-energy structures is less important than obtaining the most accurate possible CBS electronic energy (see the Supporting Information and refs (62−64) for details). The simulation results were obtained at a standard state of 1 atm and converted to values at saturation pressure ΔΔ*G*_*i*_^s^ and Δ*G*_*i*_^s^ (see the Supporting Information). We then used the G3 method to determine the G3 free energies for every structure within 1 kcal mol^–1^ from the DLPNO-CCSD(T)/CBS//ωB97xD/6-31++G** results. The Gibbs free energy changes in the stepwise monomer addition ΔΔ*G*°, using five different quantum mechanical simulation methods at 298.15 and a standard state of 1 atm, are listed in Table 1. For methods d and e in the table, we determined the DLPNO-CCSD(T)/CBS//ωB97xD/6-31++G** and G3 average free energy changes in cluster formation from monomers to clusters [*i*H_2_O > (H_2_O)_*i*_] using the lowest-Gibbs free energy (H_2_O)_*i*_ clusters. On the basis of the data in Table 1 and Figure 2, which show that the G3 energies match experiment better than the DLPNO-CCSD(T)/CBS//ωB97xD/6-31++G** energies, we repeated the methodology using Ogolem and then computed the low-lying DLPNO-CCSD(T)/CBS//ωB97xD/6-31++G** free energies for the (H_2_O)_*i*=11–14_ clusters. We then computed the G3 energies for the lowest-lying DLPNO-CCSD(T)/CBS//ωB97xD/6-31++G** clusters for (H_2_O)_*i*=11–14_. For method d, simulations were performed for eight different temperatures ranging from 216.65 to 310 K. For method e, to reveal the effect of temperature, we covered an extensive range including 16 temperatures from 200 to 373 K. All of the details about methods e and d along with values of ΔΔ*G*° and overall free energy change Δ*G*°, and their corresponding values at saturation pressure (denoted as ΔΔ*G*_*i*_^s^ and Δ*G*_*i*_^s^, respectively), are given in the Supporting Information. Table 1 shows that methods a–c and to a large extent e are consistent with one another in contrast to method d. This consistency is also reflected in panels a and b of Figure 4, which compares the simulation data in Table 1 (which are converted to the values at saturation pressure) with CNT’s prediction. We note that the differences between the G3 results for method a and method e exist because we found lower energy minima with the more comprehensive search routine, using the combination of Ogolem and DLPNO-CCSD(T)/CBS//ωB97xD/6-31++G** to locate more minima, prior to the G3 optimizations and energy calculations. More interestingly, as shown in Figure 2, the prediction by method e is in an excellent agreement with *ΔG*_2_^s^ derived from the second virial coefficient and also the experimental measurement of the water free energy change in dimerization. In addition, a similar comparison between the free energies derived from the third virial coefficients and the results from methods d and e is presented in Figure S2. This comparison also shows a better agreement (although not as clear as for Δ*G*_2_^s^ in Figure 2) between Δ*G*_3_^s^ from the third virial coefficient and the simulation results from method e. Therefore, we conclude that method e is more accurate and select it for assessing CNT’s prediction. However, for the sake of completeness, we still briefly present the comparison between the results of method d and CNT.

**Table 1:** Gibbs Free Energy Changes for the Stepwise Addition of Water Molecules at 298.15 K and 1 atm Determined by High-Level Quantum Chemical Calculations^a^

	ΔΔ*G*° (kcal mol^–1^)				
reaction	method a^54^	method b^54^	method c^55^	method d from this work	method e from this work
2H_2_O → (H_2_O)_2_	1.94	2.08		3.34	1.97
(H_2_O)_2_ + H_2_O → (H_2_O)_3_	1.83	1.43		2.18	1.62
(H_2_O)_3_ + H_2_O → (H_2_O)_4_	–1.66	–1.07		0.60	–1.50
(H_2_O)_4_ + H_2_O → (H_2_O)_5_	–0.46	–0.28		1.36	–0.40
(H_2_O)_5_ + H_2_O → (H_2_O)_6_	1.77	1.98		1.81	–0.78
(H_2_O)_6_ + H_2_O → (H_2_O)_7_			2.00	2.43	0.60
(H_2_O)_7_ + H_2_O → (H_2_O)_8_			–0.60	2.25	–2.54
(H_2_O)_8_ + H_2_O → (H_2_O)_9_			0.69	1.84	3.32
(H_2_O)_9_ + H_2_O → (H_2_O)_10_			0.66	1.86	–0.23

a The results of four methods are tabulated here. Method a using G3 from ref (54), method b using CBS-APNO from ref (54), method c using G3MP2 from ref (55), and methods d and e using DLPNO-CCSD(T)/CBS//ωb97xd/6-31++G** and G3, respectively, from this work.

**Figure 4:**
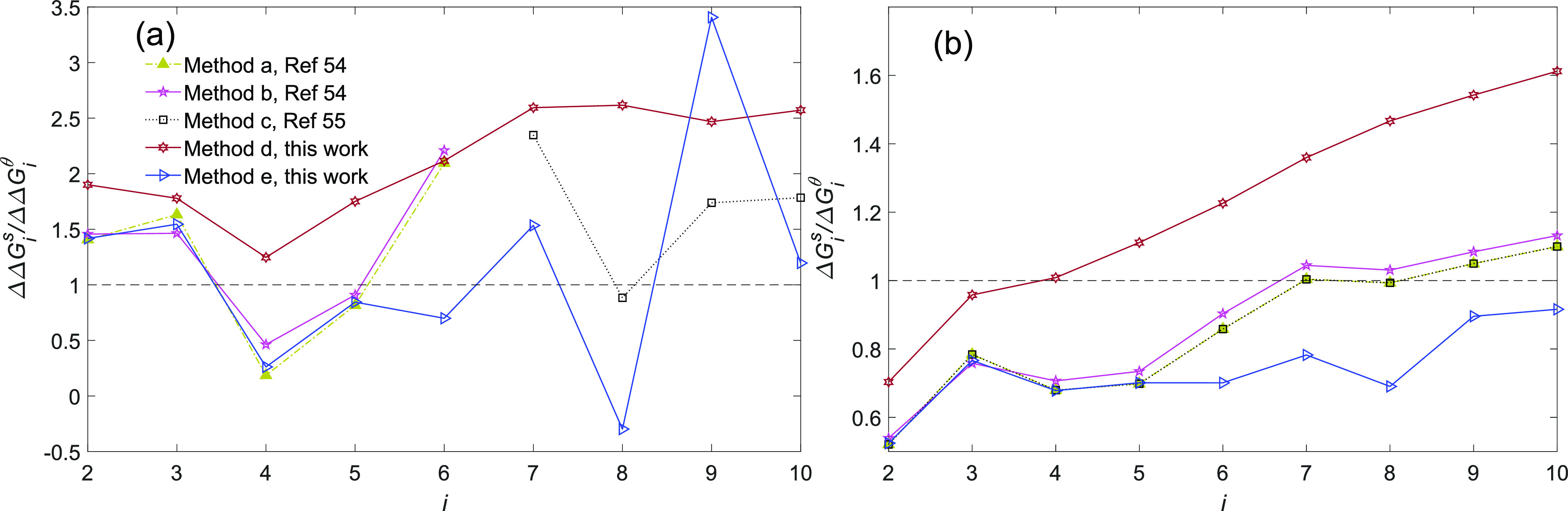
(a) Change in the free energy at saturation pressure upon stepwise monomer addition from the simulation methods, ΔΔ*G*_*i*_^s^ (see Table 1), compared to CNT’s prediction ΔΔ*G*_*i*_^θ^. (b) Overall free energy change at saturation pressure in cluster formation given from the simulation methods, Δ*G*_*i*_^s^, compared to CNT’s prediction Δ*G*_*i*_^θ^. In panel b, all of the curves extend over the entire range of *i*; in the case of methods a and b for *i* = 7–10, the data from method c are used, and in the case of method c for *i* = 1–6, the data from method a are used.

On the basis of the results from method d shown in panels a and b of Figure 5, except for tetramers at 216.65 and 230 K, CNT underpredicts the free energy change in all of the sequential cluster growth, and its departure from simulation monotonically increases with temperature. The cumulative impact of this departure is reflected in the free energy of cluster formation (see Figure 3b, where the departure of CNT from simulation increases with both temperature and cluster size). On the basis of method d, for a given temperature CNT tends to progressively underpredict the free energy compared to simulation as the cluster becomes larger, while for smaller clusters, CNT overpredicts the cluster free energies. Moreover, the crossover between overprediction and underprediction shifts toward smaller clusters with an increase in temperature. In the case of method e, in contrast to method d, no clear pattern can be observed in the behavior of ΔΔ*G*_*i*_^s^/ΔΔ*G*_*i*_^θ^ as shown in Figure 5c. Considering the overall energy change in cluster formation shown in Figure 5d, for temperatures higher than 298.15 K, the gap between CNT and simulation by method e is closing as the cluster size increases. At the higher temperatures (298.15 K < *T*), for the current cluster size range Δ*G*_*i*_^s^/Δ*G*_*i*_^θ^ exhibits a crossover for all temperatures, which is the evidence for the first scenario. For temperatures of <298.15 K, although Δ*G*_*i*_^s^/Δ*G*_*i*_^θ^ still generally increases with cluster size up to 13-mer, the rate of this increase decreases as the temperature decreases and the cluster size increases. Therefore, for the lower temperatures, it seems that the second scenario may be true. If by approaching the macroscopic sizes, Δ*G*_*i*_^s^/Δ*G*_*i*_^θ^ remains entirely below one at lower temperatures (here *T* < 298.15 K, based on the simulated cluster size range), this suggests a shift from the second scenario to the first that is governed by only temperature.

**Figure 5:**
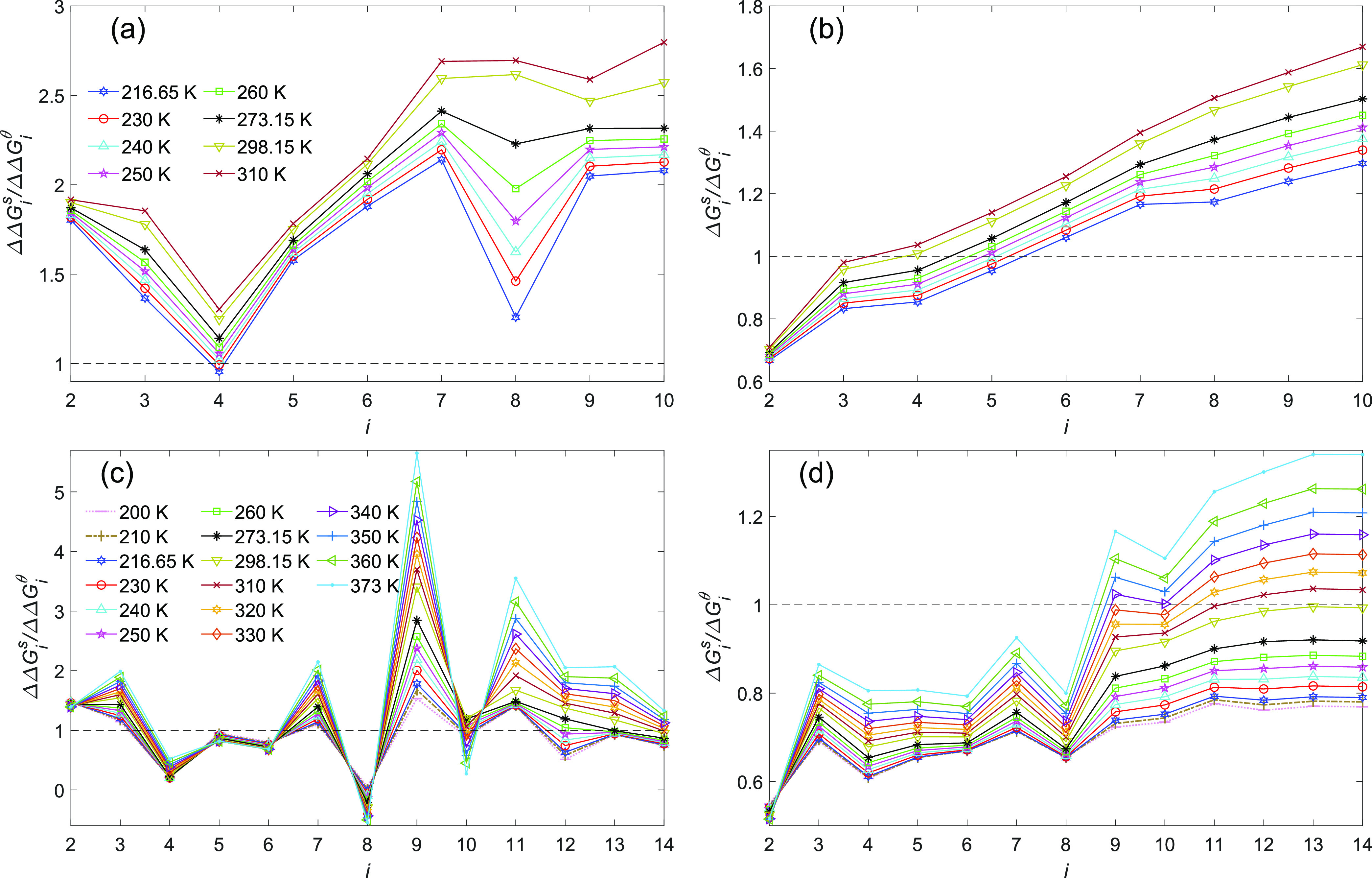
(a and b) ΔΔ*G*_*i*_^s^ and Δ*G*_*i*_^s^ from method d compared with ΔΔ*G*_*i*_^θ^ and Δ*G*_*i*_^θ^ from CNT. (c and d) ΔΔ*G*_*i*_^s^ and Δ*G*_*i*_^s^ from method e compared with ΔΔ*G*_*i*_^θ^ and Δ*G*_*i*_^θ^ from CNT.

In summary, we developed an approach for extracting the cluster free energy from nucleation experiments independent of the form of the cluster free energy. We observed that for water at temperatures above ∼250 K the extracted cluster free energy behaves nonmonotonically relative to CNT’s prediction: toward the lower end of the size range, the extracted cluster free energy is much smaller than CNT’s prediction, while an increase in size it increases it above that of CNT and the ratio between them passes through at least one maximum before approaching the macroscopic prediction. For temperatures lower than ∼250 K, the ratio of extracted free energies versus CNT’s prediction behaves differently. For these lower temperatures, almost all of the extracted free energies from experiments lie below CNT’s prediction, although the gap between them is closing as the cluster size increases. We also calculated free energies of small water clusters using state-of-the-art G3 model chemistry. We showed that G3 results are more in line with other quantum mechanical simulations and also in excellent agreement with dimers’ energy derived from the second virial coefficients of water for a wide range of temperature and also with an experimental dimerization energy measurement. G3 quantum mechanical simulations confirmed our observation of the behavior change of CNT’s prediction based on temperature, however, around a different temperature (∼298 K). It is noteworthy that, if the surface work assumes the entire free energy, our observation for higher temperatures qualitatively supports the emerging consensus between density functional theory calculations^65−67^ and molecular simulations^68−70^ that surface tension shows a nonmonotonic dependence on curvature.

## Supporting Information Available

Assumed correction to CNT in the form of exp(*A*′ + *B*′*T*)/*S* and how it compares to the correction proposed in ref (14) with respect to experimental nucleation rates of water (Figure S1), calculation of Δ*G*_2_^s^ and Δ*G*_3_^s^ from the second and third virial coefficients along with comparison between CNT and ICCT in predicting Δ*G*_3_^s^ (Figure S2), DFT structures and thermodynamic corrections, DLPNO-CCSD(T))/pVNZ (N = D < T < Q) electronic energies, Gibbs free energy values for all (H_2_O)_*i*=11–14_ clusters used in these simulations, Δ*G*° values for reactions of water monomers to make water clusters, ΔΔ*G*° values for the sequential addition of one water monomer to each proceeding water cluster, and ωb97xd xyz coordinates for each water cluster, conversion of ΔΔ*G*° and Δ*G*° (Table S6) to ΔΔ*G*^s^ (Table S7) and Δ*G*^s^ (Table S8), and all of the MP2 geometries and G3 energies for all (H_2_O)_*i*=11–14_ structures used in this work, over the temperature range of 200–373 K (PDF)
