# Supplementary material for: Probing the Free Energy of Small Water Clusters: Revisiting Classical Nucleation Theory
Source: J Phys Chem Lett. 2022 Aug 22;13(34):8038–46. doi: 10.1021/acs.jpclett.2c01361 (PMC9442792; doi:10.1021/acs.jpclett.2c01361)
Supplement: Supplementary file 1 — jz2c01361_si_001.pdf [file jz2c01361_si_001.pdf]

Supporting Information
Probing Free Energy of Small Water Clusters: Revisiting Classical Nucleation Theory

Ali Afzalifar,[1,*] George C. Shields,[2] Vance R. Fowler[2] and Robin H. A. Ras[1,3]

[1]Department of Applied Physics, Aalto University School of Science, Puumiehenkuja 2, 02150 Espoo, P.O. Box 15100, Aalto FI-00076, Finland
[2]Department of Chemistry, Furman University, Greenville, South Carolina 29613, United States
[3]Department of Bioproducts and Biosystems, Aalto University School of Chemical Engineering, P.O. Box 16000, Aalto FI-00076, Finland

*Corresponding author.

Email: ali.afzalifar@aalto.fi

1 CORRECTION TO CNT AS A FUNCTION OF ONLY SUPERSATURATION

Wölk and Strey[S1] proposed an empirical correction as a function of only temperature to bring CNT prediction in line with the results of their nucleation rate experiment on water. This correction reads

$$\exp(A + B/T) \qquad \text{(S1)}$$

where $A = -27.56$ and $B = 6.5 \times 10^3$. Following a similiar procedure as described by Wölk and Strey (i.e. all data points on $J_{exp}/(J_{CNT}/S)$ for each isotherm collpases into a single point to derive the correction), a correction with the follwoing form also can be assumed

$$\exp(A' + B'T)/S \qquad \text{(S2)}$$

where $A' = 30.33$ and $B' = -0.12$. Based on the comparison with the nucleation rate experiments of water in Figure S1, it seems that both corrections are (in genral) equally successful to improve the prediction of nucleation rate by CNT, given by Eq. (1) in the main text. However, the correction defined by Eq. (S2) has the advantage of being compatibale with both the law of mass action and the first nucleation theorem. See

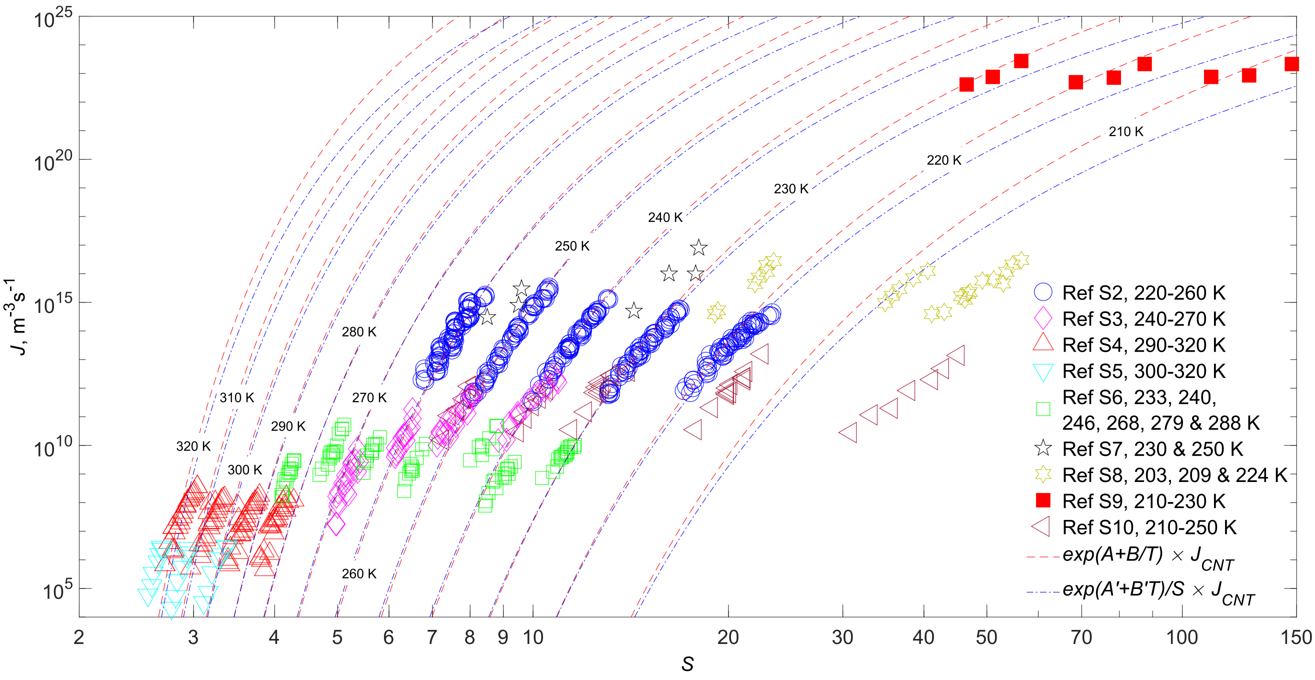

Figure S1. Comparison of nucleation rate J versus supersaturation S, given by corrections to CNT defined by Eq. (S1) and Eq. (S2) with respect to experimental rates reported in Refs S2-S10.

2 CALCULATION OF ΔG_2^s FROM THE SECOND VIRIAL COEFFICIENT AND THE ASSOCIATED UNCERTAINTY

Neglecting the cluster-cluster interactions and using Dalton's law, the total vapor pressure can be computed as a function of partial pressures of all cluster sizes

$$P_v = k_B T \sum_{i=1}^{\infty} n_i \quad \text{(S3)}$$

where n_i is the steady-state number density of an i-mer. In addition, the virial equation of the state defines the pressure of an imperfect vapor in form of series of virial coefficients as

$$P_v = k_B T (n_t + \sum_{i=2}^{\infty} B_i n_t^i) \quad \text{(S4)}$$

where B_i is the ith virial coefficient and n_t is the total number density which reads as $n_t = \sum_{i=1}^{\infty} i n_i$. By equating Eq. (S3) to Eq. (S4) a set of direct relations in form of $n_i = f(B_2, B_3, ...)$ between the population of clusters and the virial series coefficients is obtained. The derivation of $n_i = f(B_1, B_2, ...)$ was explained in detail in Ref S11 and is not repeated here. We only re-present the relations for n_1, n_2 and n_2 at the saturation pressure based on the virial series truncated up to B_3

$$n_i = \frac{P_s}{k_B T} K_i (\beta_1)^i \quad \text{(S5)}$$

where

$$K_1 = 1, \tag{S6}$$

$$K_2 = B_2 \frac{P_s}{-k_B T} \tag{S7}$$

$$K_3 = \frac{-1}{2}\left(B_3\left(\frac{P_s}{k_B T}\right)^2 - 4K_2^2\right) \tag{S8}$$

and $\beta_{\mathrm{i}} = \sum_{j=1}^{i} a_j$ in which $a_1 = K_1$, $a_2 = -K_2$ and $a_3 = 2{K_2}^2 - K_3$. Then, knowing n_i^s the cluster free energy is calculated (by rewriting Eq. (2) in the main text and replacing n_1^s with n^s) as

$$\frac{\Delta G_i^s}{k_B T} = \ln\left(\frac{P_s}{n_i^s k_B T}\right). \tag{S9}$$

Figure S2 shows ΔG_3^θ by CNT, ICCT and the ones calculated by Eq. (S9) using relations for the third virial coefficients[S12-S14].

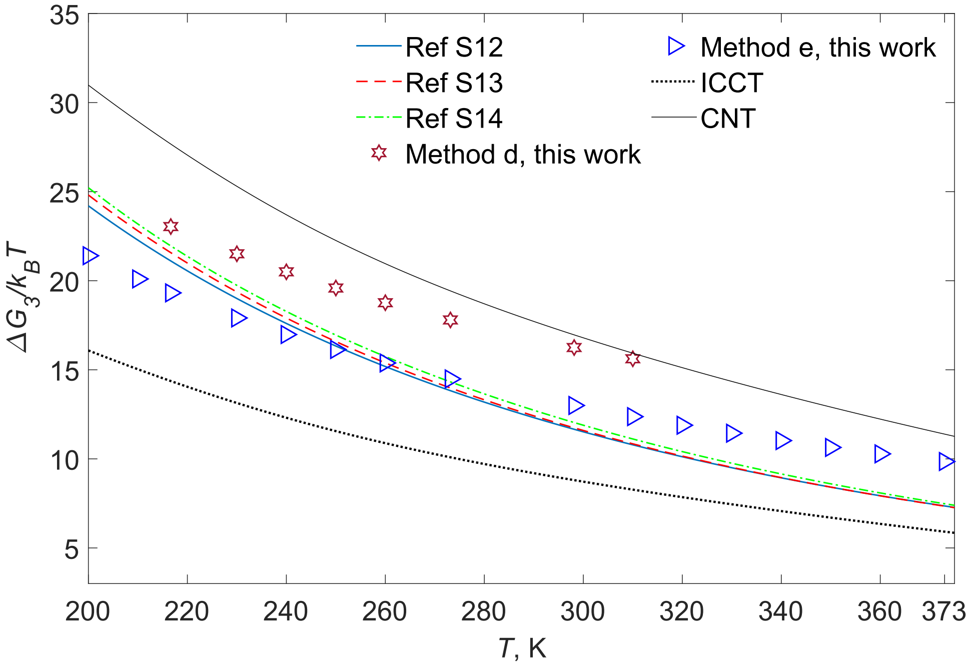

Figure S2. ΔG_3^s as a function of temperature form virial coefficients in Refs S12-S14, and simulation methods of d and e from this work, and predictions of by CNT and ICCT.

3 SIMULATION DETAILS

ωB97XD/6-31++G** coordinate files, G correction values at each Temperature, DLPNO-CCSD(T) electronic energies with the cc-pVNZ basis sets (N=D,T,Q), calculation of $\Delta\Delta G^{\circ}$, ΔG° and their conversion to $\Delta\Delta G^s$ and ΔG^s for each reaction, and xyz coordinates of all structures used in this work are presented in this section.

3.1 CCSD(T) Energies and Structure ID label for coordinates and conversion to ΔG^S and $\Delta\Delta G^S$

Note that G corrections include zero-point vibrational energy, and the minimum ΔG° structure often changes with temperature, and is often different from the Electronic Energy Minimum.

Table S1. ωB97Xd G correction values at various temperatures (kcal/mol).

ωB97XD Geometry										
Molecular Formula	Structure ID	Symmetry	G (216.56)	G (230)	G (240)	G (250)	G (260)	G (273.15)	G (298.15)	G (310)
H_2O	6	C2V	5.753	5.182	4.751	4.316	3.878	3.297	2.178	1.643
$(H_2O)_2$	102	C1	17.88	17.031	16.385	15.733	15.074	14.197	12.5	11.682
$(H_2O)_3$	211	C1	33.23	32.269	31.538	30.797	30.046	29.045	27.097	26.155
$(H_2O)_4$	92	S4	48.144	47.051	46.216	45.369	44.509	43.361	41.12	40.034
$(H_2O)_4$	531	Ci	47.479	46.358	45.503	44.635	43.755	42.578	40.284	39.171
$(H_2O)_5$	410	C1	61.34	60.038	59.043	58.032	57.005	55.63	52.946	51.642
$(H_2O)_6$	448		74.866	73.374	72.232	71.07	69.889	68.307	65.211	63.706
	1043		74.137	72.606	71.436	70.245	69.035	67.415	64.246	62.705
$(H_2O)_7$	494		88.974	87.314	86.042	84.748	83.431	81.666	78.208	76.524
$(H_2O)_8$	725	S4	109.944	108.443	107.289	106.111	104.909	103.293	100.112	98.556
	392	D2D	109.918	108.414	107.258	106.078	104.874	103.256	100.071	98.513
	1040	C1	105.083	103.347	102.015	100.657	99.274	97.418	93.774	91.997
	346		103.447	101.622	100.224	98.8	97.35	95.405	91.593	89.735
$(H_2O)_9$	225		123.922	122.257	120.977	119.668	118.333	116.537	112.998	111.266
	299		119.077	117.165	115.697	114.201	112.676	110.628	106.605	104.642
$(H_2O)_{10}$	286		137.72	135.879	134.463	133.016	131.538	129.549	125.63	123.71
	172		139.776	138.028	136.683	135.307	133.902	132.009	128.273	126.442

Table S2. DLPNO-CCSD(T)/cc-pVNZ//ωb97xd Electronic Energy (Hartrees)

Molecular Formula	Structure ID	cc-pVDZ	cc-pVTZ	cc-pVQZ	TDQ Complete Basis Set (CBS) Extrapolation
H_2O	6	-76.24080669	-76.33195511	-76.35953756	-76.380
$(H_2O)_2$	102	-152.4927068	-152.6730858	-152.7274598	-152.7686059
$(H_2O)_3$	211	-228.7572709	-229.0249742	-229.1051057	-229.1653976
$(H_2O)_4$	92	-305.02251	-305.3777635	-305.4839693	-305.5637989
$(H_2O)_4$	531	-305.0205211	-305.3760523	-305.4823964	-305.5623639
$(H_2O)_5$	410	-381.2804455	-381.7247512	-381.8576027	-381.9574744
$(H_2O)_6$	448	-457.538292	-458.0715399	-458.2310559	-458.3510159
	1043	-457.5362543	-458.0697107	-458.2294187	-458.349603
$(H_2O)_7$	494	-533.7998731	-534.4202868	-534.6056857	-534.744992
$(H_2O)_8$	725	-610.0879533	-610.7895663	-610.9976236	-611.1529657
	392	-610.0880357	-610.7895884	-610.9975118	-611.1526818
	1040	-610.0677892	-610.7740415	-610.9847655	-611.1428999
	346	-610.0612704	-610.7698464	-610.9811870	-611.1397369
$(H_2O)_9$	225	-686.346923	-687.1376873	-687.3723255	-687.5476029
	299	-686.3277075	-687.122767	-687.3598704	-687.5377282
$(H_2O)_{10}$	286	-762.60474	-763.48493	-763.74648	-763.94209
	172	-762.61311	-763.49047	-763.7507	-763.94502

1H= 4.359 744 722 2071(85) × 10^{-18} J			
Hartree	4.36 E-18		Hartree to kcal/mol
Nav	6.02 E+23	6.28 E+02	627.5095

Table S3. Monomer and Cluster Minimum Energy G Values for each Temperature at DLPNO-CCSD(T)/CBS/ωB97XD level of theory (kcal/mol)

Molecular Formula	Structure ID	Symmetry	G (216.56)	G (230)	G (240)	G (250)	G (260)	G (273.15)	G (298.15)	G (310)

H_2O	6	C2V	-47923.719	-47924.29	-47924.721	-47925.156	-47925.594	-47926.175	-47927.294	-47927.829
$(H_2O)_2$	102	C1	-95845.869	-95846.718	-95847.364	-95848.016	-95848.675	-95849.552	-95851.249	-95852.067
$(H_2O)_3$	211	C1	-143770.23	-143771.19	-143771.92	-143772.66	-143773.41	-143774.42	-143776.36	-143777.31
$(H_2O)_4$	92	S4	-191696.04	-191697.13	-191697.97	-191698.81	-191699.67	-191700.82	-191703.06	-191704.15
$(H_2O)_4$	531	Ci	-191695.8	-191696.92	-191697.78	-191698.65	-191699.53	-191700.7	-191703	-191704.11
$(H_2O)_5$	410	C1	-239620.6	-239621.9	-239622.89	-239623.91	-239624.93	-239626.31	-239628.99	-239630.3
$(H_2O)_6$	448		-287544.74	-287546.24	-287547.38	-287548.54	-287549.72	-287551.3	-287554.4	-287555.9
$(H_2O)_6$	1043		-287544.59	-287546.12	-287547.29	-287548.48	-287549.69	-287551.31	-287554.48	-287556.02
$(H_2O)_7$	494		-335468.58	-335470.24	-335471.51	-335472.81	-335474.12	-335475.89	-335479.35	-335481.03
$(H_2O)_8$	725	S4	-383394.34	-383395.84	-383396.99	-383398.17	-383399.37	-383400.99	-383404.17	-383405.73
$(H_2O)_8$	392	D2D	-383394.19	-383395.69	-383396.85	-383398.03	-383399.23	-383400.85	-383404.03	-383405.59
$(H_2O)_8$	1040	C1	-383392.88	-383394.62	-383395.95	-383397.31	-383398.69	-383400.55	-383404.19	-383405.97
$(H_2O)_8$	346		-383392.53	-383394.36	-383395.76	-383397.18	-383398.63	-383400.58	-383404.39	-383406.25
$(H_2O)_9$	225		-431318.72	-431320.38	-431321.66	-431322.97	-431324.31	-431326.1	-431329.64	-431331.38
$(H_2O)_9$	299		-431317.37	-431319.28	-431320.75	-431322.24	-431323.77	-431325.82	-431329.84	-431331.8
$(H_2O)_{10}$	286		-479243.19	-479245.03	-479246.44	-479247.89	-479249.37	-479251.36	-479255.28	-479257.2
$(H_2O)_{10}$	172		-479242.97	-479244.72	-479246.06	-479247.44	-479248.84	-479250.74	-479254.47	-479256.3

Table S4. ΔG° values for reactions to make $(H_2O)_i$ clusters at the DLPNO-CCSD(T)/CBS//ωb97xd/6-31++G** level of theory (kcal/mol).

Reaction	Cluster Structure ID and Symmetry	ΔG (216.65)	ΔG (230)	ΔG (240)	ΔG (250)	ΔG (260)	ΔG (273.15)	ΔG (298.15)	ΔG (310)
2 H_2O > $(H_2O)_2$	102	1.57	1.86	2.08	2.30	2.51	2.80	3.34	3.59
3 H_2O > (H2O)3	211	0.93	1.68	2.24	2.81	3.37	4.11	5.52	6.18
4 H_2O > (H_2O)4	92 S4	-1.16	0.03	0.92	1.81	2.70	3.88	6.12	7.17

4 H_2O > $(H_2O)_4$	531 Ci	-0.92	0.24	1.11	1.98	2.85	4.00	6.18	7.21
5 H_2O > $(H_2O)_5$	410	-2.00	-0.45	0.71	1.88	3.04	4.57	7.48	8.85
6 H_2O > $(H_2O)_6$	448	-2.43	-0.49	0.95	2.40	3.85	5.75	9.37	11.07
6 H_2O > $(H_2O)_6$	1043	-2.27	-0.37	1.04	2.46	3.88	5.74	9.29	10.96
7 H_2O > $(H_2O)_7$	494	-2.54	-0.21	1.54	3.29	5.04	7.34	11.71	13.78
8 H_2O > $(H_2O)_8$	725 S4	-4.58	-1.52	0.78	3.08	5.38	8.41	14.18	16.91
8 H_2O > $(H_2O)_8$	392 D2D	-4.43	-1.37	0.92	3.22	5.52	8.55	14.32	17.04
8 H_2O > $(H_2O)_8$	1040 C1	-3.13	-0.30	1.82	3.94	6.06	8.86	14.16	16.67
8 H_2O > $(H_2O)_8$	346	-2.78	-0.04	2.01	4.07	6.12	8.83	13.97	16.39
9 H_2O > $(H_2O)_9$	225	-5.25	-1.77	0.83	3.43	6.04	9.47	16.01	19.09
9 H_2O > $(H_2O)_9$	299	-3.89	-0.67	1.74	4.16	6.58	9.76	15.81	18.66
10 H_2O > $(H_2O)_{10}$	286	-5.99	-2.12	0.77	3.67	6.57	10.40	17.67	21.10
10 H_2O > $(H_2O)_{10}$	172	-5.78	-1.81	1.15	4.13	7.10	11.02	18.47	21.99

Table S5. Free energy change for sequential reactions ($\Delta\Delta G^{\circ}$) $H_2O + (H_2O)_{i-1} > (H_2O)_i$ using the lowest energy ΔG° for each structure (varies with Temperature) at the DLPNO-CCSD(T)/CBS/cc-pVnZ//ωb97xd/6-31++G** level.

Sequential Hydration reaction (kcal/mol)									
Reaction	i	216.65 K	230 K	240 K	250 K	260 K	273.15K	298.15 K	310 K
2 H_2O > $(H_2O)_2$	2	1.57	1.86	2.08	2.30	2,51	2,80	3.34	3.59
$H_2O + (H_2O)_2 > (H_2O)_3$	3	-0.64	-0.18	0.16	0.51	0.86	1.31	2.18	2.59
$H_2O + (H_2O)_3 > (H_2O)_4$	4	-2.09	-1.65	-1.32	-0.99	-0.66	-0.23	0.60	0.99
$H_2O + (H_2O)_4 > (H_2O)_5$	5	-0.84	-0.48	-0.21	0,06	0.33	0.69	1.36	1.68
$H_2O + (H_2O)_5 > (H_2O)_6$	6	-0.43	-0.05	0.24	0.52	0.81	1.18	1.81	2.11
$H_2O + (H_2O)_6 > (H_2O)_7$	7	-0.12	0.29	0.59	0.89	1.19	1.60	2.43	2.82
$H_2O + (H_2O)_7 > (H_2O)_8$	8	-2.04	-1.31	-0.76	-0.21	0.34	1.07	2.25	2.61
$H_2O + (H_2O)_8 > (H_2O)_9$	9	-0.66	-0.26	0.05	0.35	0.66	1.06	1.84	2.27
$H_2O + (H_2O)_9 > (H_2O)_{10}$	10	-0.75	-0.35	-0.06	0.24	0.53	0.92	1.86	2.44

Table S6. ΔG° for reactions i $H_2O > (H_2O)_i$ using the lowest energy ΔG° for each structure (varies with Temperature) at the DLPNO-CCSD(T)/CBS/cc-pVnZ//ωb97xd/6-31++G** level.

Overall hydration reactions (kcal/mol)									
Reaction	i	216.65 K	230 K	240 K	250 K	260 K	273.15K	298.15 K	310 K
2 H_2O > $(H_2O)_2$	2	1.57	1.86	2.08	2.30	2.51	2.80	3.34	3.59
3 H_2O > $(H_2O)_3$	3	0.93	1.68	2.24	2.81	3.37	4.11	5.52	6.18
4 H_2O > $(H_2O)_4$	4	-1.16	0.03	0.92	1.81	2.70	3.88	6.12	7.17
5 H_2O > $(H_2O)_5$	5	-2.00	-0.45	0.71	1.88	3.04	4.57	7.48	8.85
6 H_2O > $(H_2O)_6$	6	-2.43	-0.49	0.95	2.40	3.85	5.74	9.29	10.96
7 H_2O > $(H_2O)_7$	7	-2.54	-0.21	1.54	3.29	5.04	7.34	11.71	13.78
8 H_2O > $(H_2O)_8$	8	-4.58	-1.52	0.78	3.08	5.38	8.41	13.97	16.39
9 H_2O > $(H_2O)_9$	9	-5.25	-1.77	0.83	3.43	6.04	9.47	15.81	18.66
10 H_2O > $(H_2O)_{10}$	10	-5.99	-2.12	0.77	3.67	6.57	10.40	17.67	21.10

The data in Table S5 (which are at a standard state of 1 atm) is converted by the following relations to the corresponding values at saturation pressure

$$\Delta\Delta G_i^s = (\Delta\Delta G_i^{\circ}/k_B T_v) \times (4.184 \times 10^3) - \ln(P_s), \qquad (S9)$$

$$\Delta G_i^s = \sum_{l=2}^{i} \Delta\Delta G_l^s \qquad (S10)$$

where P_s is in atm. The converted values are tabulated in Table S7 and Table S8. Alternatively, ΔG_i^s can be also calculated directly from the simulation results in Table S6 as

$$\Delta G_i^s = (\Delta G_i^{\circ}/k_B T_v) \times (4.184 \times 10^3) - (i-1)\ln(P_s). \qquad (S11)$$

Table S7. Free energy change for sequential reactions $H_2O + (H_2O)_{i-1} > (H_2O)_i$ at saturation pressure ($\Delta\Delta G^S$), obtained from the data in Table S5 using Eq. (S9).

Sequential Hydration reaction at saturation pressure (k_BT)									
Reaction	i	216.65 K	230 K	240 K	250 K	260 K	273.15K	298.15 K	310 K
$2\ H_2O > (H_2O)_2$	2	14.09	12.99	12.26	11.59	10.99	10.27	9.10	8.62
$H_2O + (H_2O)_2 > (H_2O)_3$	3	8.95	8.52	8.24	8.00	7.78	7.53	7.15	7.00
$H_2O + (H_2O)_3 > (H_2O)_4$	4	5.59	5.31	5.13	4.97	4.84	4.69	4.47	4.39
$H_2O + (H_2O)_4 > (H_2O)_5$	5	8.49	7.87	7.46	7.10	6.77	6.38	5.77	5.52
$H_2O + (H_2O)_5 > (H_2O)_6$	6	9.45	8.82	8.40	8.02	7.68	7.28	6.52	6.21
$H_2O + (H_2O)_6 > (H_2O)_7$	7	10.17	9.54	9.13	8.76	8.43	8.05	7.56	7.36
$H_2O + (H_2O)_7 > (H_2O)_8$	8	5.71	6.05	6.31	6.55	6.79	7.09	7.27	7.03
$H_2O + (H_2O)_8 > (H_2O)_9$	9	8.90	8.36	8.00	7.68	7.40	7.06	6.58	6.48
$H_2O + (H_2O)_9 > (H_2O)_{10}$	10	8.71	8.15	7.78	7.45	7.16	6.81	6.60	6.74

Table S8. Free energy change for reactions i $H_2O > (H_2O)_i$ at saturation pressure (ΔG^S), obtained from the data in Table S5 using Eq. (S10), or from the data in Table S6 using Eq. (S11).

Overall hydration reactions at saturation pressure (k_BT)									
Reaction	i	216.65 K	230 K	240 K	250 K	260 K	273.15K	298.15 K	310 K
$2\ H_2O > (H_2O)_2$	2	14.09	12.99	12.26	11.59	10.99	10.27	9.10	8.62
$3\ H_2O > (H_2O)_3$	3	23.04	21.51	20.50	19.59	18.76	17.80	16.25	15.62
$4\ H_2O > (H_2O)_4$	4	28.63	26.82	25.63	24.56	23.60	22.49	20.72	20.01
$5\ H_2O > (H_2O)_5$	5	37.12	34.69	33.09	31.66	30.37	28.87	26.49	25.53
$6\ H_2O > (H_2O)_6$	6	46.57	43.51	41.49	39.68	38.06	36.14	33.01	31.74
$7\ H_2O > (H_2O)_7$	7	56.75	53.06	50.62	48.45	46.48	44.19	40.57	39.11
$8\ H_2O > (H_2O)_8$	8	62.45	59.11	56.93	55.00	53.27	51.29	47.84	46.14
$9\ H_2O > (H_2O)_9$	9	71.36	67.47	64.93	62.68	60.67	58.35	54.41	52.62
$10\ H_2O > (H_2O)_{10}$	10	80.06	75.62	72.71	70.13	67.83	65.16	61.02	59.36

3.2 ωB97XD/6-31++G** coordinate files for all structures

```
Molecular Formula        Structure
H2O                              1
1
3
wb97xd.log
O      0.00000     0.00000     0.11570
H     -0.00000     0.76652    -0.46279
H     -0.00000    -0.76652    -0.46279


(H2O)2                           102
6
Ogolem-102-17087-wb97xd.log
O     -1.49422    -0.00001     0.12069
H     -1.90654     0.00008    -0.74566
H     -0.53805    -0.00002    -0.03859
O      1.36652     0.00001    -0.10723
H      1.73308    -0.76877     0.33822
H      1.73306     0.76872     0.33834
```

(H2O)3 211
9
Ogolem-211-1468-wb97xd.log

O	1.45412	-0.65932	-0.08268
H	0.58679	-1.10438	-0.06842
H	2.00188	-1.08793	0.57821
O	-0.14610	1.58378	0.10899
H	-0.07808	2.22772	-0.59947
H	0.65850	1.03336	0.06678
O	-1.30532	-0.91580	-0.08985
H	-1.96560	-1.19216	0.54892
H	-1.22507	0.05411	-0.01773

(H2O)4 92
12
Ogolem-92-39644-wb97xd.log

O	0.13397	-1.91304	-0.02800
H	0.88131	-1.27456	0.00651
H	0.29078	-2.48046	-0.78605
O	-1.91306	-0.13398	0.02800
H	-2.48042	-0.29076	0.78610
H	-1.27456	-0.88130	-0.00650
O	1.91305	0.13398	0.02800
H	1.27456	0.88131	-0.00649
H	2.48049	0.29079	0.78604
O	-0.13398	1.91303	-0.02800
H	-0.29078	2.48048	-0.78603
H	-0.88132	1.27454	0.00647

(H2O)4 531
12
Ogolem-531-13894-wb97xd.log

O	1.33987	1.37966	-0.09043
H	1.70312	1.93294	0.60444
H	0.36832	1.52528	-0.09144
O	1.38665	-1.32836	0.09301
H	1.50437	-0.35385	0.03624
H	1.96744	-1.71512	-0.56558
O	-1.33988	-1.37966	0.09039
H	-0.36833	-1.52527	0.09148
H	-1.70308	-1.93300	-0.60445
O	-1.38663	1.32837	-0.09297
H	-1.50438	0.35386	-0.03628
H	-1.96748	1.71511	0.56556

(H2O)5 410
15
Ogolem-410-39738-wb97xd.log

O	-1.75797	1.47114	-0.03795
H	-0.81095	1.74052	-0.07132

H	-2.19100	2.07199	0.57180
O	0.86287	2.12162	-0.09346
H	1.19925	2.55064	-0.88358
H	1.41588	1.31709	0.03844
O	2.28433	-0.15618	0.18103
H	1.69405	-0.93404	0.04957
H	2.78230	-0.32388	0.98408
O	0.56641	-2.21091	-0.14661
H	0.60869	-2.71735	-0.96091
H	-0.36152	-1.89015	-0.06739
O	-1.94411	-1.23438	0.03881
H	-1.94460	-0.25055	0.01685
H	-2.48426	-1.49461	0.78793

(H2O)6 448
18
Ogolem-448-33346-wb97xd.log

O	-2.65011	0.38595	0.14699
H	-3.15481	0.58252	0.93922
H	-2.03376	1.14350	0.01786
O	1.65916	2.10170	0.14701
H	2.08140	2.44038	0.93948
H	2.00728	1.18926	0.01778
O	-0.99064	2.48751	-0.14702
H	-1.07257	3.02249	-0.93955
H	-0.02642	2.33261	-0.01771
O	-1.65916	-2.10171	-0.14700
H	-2.08143	-2.44042	-0.93944
H	-2.00729	-1.18927	-0.01778
O	2.65011	-0.38595	-0.14700
H	3.15478	-0.58250	-0.93926
H	2.03374	-1.14348	-0.01787
O	0.99064	-2.48750	0.14703
H	0.02642	-2.33258	0.01775
H	1.07264	-3.02250	0.93953

(H2O)6 1043
18
Ogolem-1043-16870-wb97xd.log

O	0.82056	-2.35319	0.65761
H	1.04495	-2.31796	1.59000
H	1.46113	-1.76101	0.19960
O	-1.70656	-1.95578	-0.20214
H	-2.23865	-2.69391	0.10102
H	-0.79123	-2.10866	0.12823
O	-0.80307	2.45506	0.40954
H	-1.08833	2.73317	1.28271
H	-1.45085	1.77389	0.11449
O	2.52008	-0.64905	-0.56801
H	2.58137	-0.72016	-1.52305
H	2.29273	0.28691	-0.36881

O	-2.59576	0.58508	-0.34475
H	-2.29015	-0.34681	-0.26015
H	-2.95387	0.66953	-1.23127
O	1.81102	1.88393	0.05819
H	0.85533	2.07965	0.19124
H	2.20749	2.67696	-0.30750

(H2O)6 346
18
Ogolem-346-37087-wb97xd.log

O	-0.46198	0.24310	-0.01501
H	-1.07057	0.50469	0.71029
H	0.15215	0.99295	-0.19098
O	1.32772	2.20678	-0.48002
H	1.33614	2.63142	-1.34076
H	2.23955	1.86627	-0.32899
O	3.51843	-1.52160	0.16515
H	2.57248	-1.75727	0.30598
H	3.83886	-2.09139	-0.53761
O	-4.29896	-0.10671	-0.03943
H	-4.98506	0.43979	-0.42824
H	-3.67751	-0.33906	-0.76575
O	0.88626	-2.04596	0.48585
H	0.58764	-2.39110	1.33068
H	0.38101	-1.21330	0.33293
O	-2.31004	-0.60783	-1.81513
H	-2.09877	-1.51379	-2.05328
H	-1.55533	-0.28224	-1.27493
O	3.78576	1.15400	-0.09808
H	3.72966	0.17382	-0.01912
H	4.31213	1.45587	0.64558
O	-2.43560	0.63541	1.80583
H	-3.22024	0.42498	1.25374
H	-2.63484	1.44091	2.28720

(H2O)7 494
21
Ogolem-494-38873-wb97xd.log

O	1.83434	2.20596	0.01742
H	0.93652	1.94679	0.34623
H	1.70402	2.89546	-0.63655
O	-2.96092	1.48737	-0.47359
H	-3.02485	1.84388	-1.36200
H	-3.10739	0.52378	-0.54804
O	3.31469	0.03740	-0.47687
H	4.08079	0.10770	0.09732
H	2.78891	0.86292	-0.33769
O	-0.53110	1.33553	0.89539
H	-1.36906	1.54660	0.44128
H	-0.51651	0.36679	0.96657
O	-2.94469	-1.26217	-0.40589

H	-2.08630	-1.47694	0.01042
H	-3.15623	-1.97169	-1.01499
O	1.78445	-2.14092	-0.26987
H	2.37652	-1.35121	-0.33926
H	1.82886	-2.59068	-1.11641
O	-0.52857	-1.53385	0.87835
H	-0.51667	-1.94587	1.74607
H	0.31584	-1.79211	0.42752

(H2O)8 725
24
Ogolem-725-36695-wb97xd.log

O	-0.59883	1.81042	1.31834
H	-1.16127	1.04134	1.52042
H	0.31133	1.52853	1.52033
O	1.95661	0.64701	1.32551
H	2.79711	0.92471	1.69629
H	2.03367	0.67265	0.33389
O	-1.95639	-0.64744	1.32562
H	-2.03359	-0.67279	0.33401
H	-2.79683	-0.92526	1.69644
O	0.59902	-1.81079	1.31780
H	1.16149	-1.04176	1.52000
H	-0.31111	-1.52896	1.52001
O	-1.81071	-0.59876	-1.31804
H	-1.04166	-1.16119	-1.52027
H	-1.52885	0.31142	-1.52003
O	-0.64735	1.95667	-1.32526
H	-0.67277	2.03367	-0.33363
H	-0.92517	2.79718	-1.69591
O	1.81050	0.59915	-1.31811
H	1.04141	1.16164	-1.52006
H	1.52861	-0.31097	-1.52033
O	0.64715	-1.95626	-1.32587
H	0.67271	-2.03355	-0.33427
H	0.92494	-2.79665	-1.69682

(H2O)8 392
24
Ogolem-392-39440-wb97xd.log

O	1.34629	-1.85859	-0.29780
H	0.40743	-2.10392	-0.38397
H	1.50717	-1.20851	-1.00516
O	1.46019	-0.43555	1.92512
H	1.51932	-1.04905	1.14370
H	2.08107	-0.74155	2.58991
O	-1.34608	0.29731	-1.85885
H	-1.50730	1.00462	-1.20882
H	-0.40720	0.38381	-2.10400
O	-1.45937	-1.92567	-0.43580
H	-2.08000	-2.59069	-0.74182

H	-1.51873	-1.14428	-1.04930
O	-1.34551	-0.29802	1.85909
H	-0.40654	-0.38403	2.10406
H	-1.50650	-1.00544	1.20911
O	1.34525	1.85934	0.29755
H	1.50663	1.20939	1.00490
H	0.40624	2.10405	0.38383
O	-1.46036	1.92487	0.43605
H	-2.08120	2.58964	0.74219
H	-1.51922	1.14349	1.04960
O	1.45960	0.43631	-1.92536
H	2.08025	0.74261	-2.59024
H	1.51854	1.04984	-1.14396

(H2O)8 1040
24
Ogolem-1040-29759-wb97xd.log

O	0.18863	-0.11404	-1.27993
H	0.12545	-0.03358	-0.30017
H	1.15281	-0.14536	-1.45841
O	-1.30266	-2.40008	-1.31230
H	-0.85881	-3.15166	-1.71107
H	-0.72452	-1.62001	-1.46168
O	-1.36977	2.49327	1.09036
H	-0.98384	3.32670	1.36847
H	-1.38895	2.50305	0.10818
O	2.60311	0.01389	1.50290
H	2.94462	-0.59508	2.16089
H	1.62918	0.04877	1.62304
O	-0.10761	0.12087	1.43170
H	-0.59459	0.97254	1.48938
H	-0.73568	-0.61220	1.60256
O	-1.23023	2.19892	-1.62059
H	-2.03722	2.03261	-2.11354
H	-0.69744	1.37610	-1.67299
O	2.92122	-0.22239	-1.19420
H	3.47547	0.48645	-1.52842
H	2.95365	-0.16349	-0.21466
O	-1.78745	-2.00754	1.35626
H	-1.88949	-2.79570	1.89237
H	-1.69267	-2.29237	0.42249

(H2O)9 225
27
Ogolem-225-38694-wb97xd.log

O	1.39200	-2.01315	0.14994
H	0.81394	-1.97928	-0.63387
H	0.81200	-1.80954	0.90596
O	1.69857	1.96930	-0.14744
H	2.36875	1.23852	-0.09173
H	2.17717	2.78663	-0.30335

O	-0.45442	-1.50862	-1.89957
H	-0.47061	-1.89791	-2.77674
H	-0.35745	-0.52417	-2.00707
O	-0.42034	-1.12695	2.10264
H	-0.34145	-0.13572	2.05697
H	-0.44747	-1.37593	3.02916
O	3.26986	-0.15116	-0.06410
H	2.62475	-0.90241	0.01284
H	3.97771	-0.32320	0.56012
O	-2.45364	1.38128	-0.10518
H	-2.57343	0.39619	-0.02496
H	-3.32304	1.78535	-0.14561
O	-0.32610	1.13526	-1.91956
H	-1.11895	1.39860	-1.41945
H	0.43143	1.46976	-1.40540
O	-0.33366	1.49067	1.72815
H	-1.12635	1.65190	1.18562
H	0.42347	1.75103	1.17241
O	-2.39539	-1.25187	0.11039
H	-1.85451	-1.53510	-0.64882
H	-1.83101	-1.39264	0.89179

(H2O)9 299
27
Ogolem-299-38111-wb97xd.log

O	1.97998	-2.68827	0.10679
H	2.08472	-2.29702	-0.78702
H	1.62278	-3.57027	-0.01767
O	-2.03652	-0.94166	2.10127
H	-2.07825	-0.46472	2.93326
H	-1.10224	-0.88743	1.79435
O	1.44738	2.86868	-0.11431
H	1.01770	3.69939	0.09970
H	1.69506	2.45047	0.73980
O	0.29581	0.67924	-1.25764
H	-0.62950	0.79461	-1.56963
H	0.64931	1.55646	-0.99588
O	0.46700	-0.65712	1.11152
H	0.32997	-0.20898	0.24422
H	0.97846	-1.46776	0.89381
O	-3.70981	-0.27269	0.08528
H	-4.22803	-1.06034	-0.09501
H	-3.12989	-0.49569	0.84949
O	2.01207	-1.20731	-2.17021
H	1.39098	-0.46971	-1.98388
H	1.99796	-1.36566	-3.11555
O	1.94587	1.40693	2.13110
H	1.46068	0.58740	1.88940
H	2.85086	1.14273	2.31363
O	-2.31420	0.81420	-1.96084
H	-2.76010	1.62336	-2.21971

H	-2.85112	0.41719	-1.23696

(H2O)10 286
30
Ogolem-286-39443-wb97xd.log

O	-0.82614	-0.75058	2.31918
H	-1.00729	0.19990	2.20034
H	0.13425	-0.85338	2.19523
O	-1.12350	1.94553	1.62531
H	-1.65696	2.60297	2.07725
H	-1.40642	1.92941	0.67048
O	1.92662	-0.86045	1.73426
H	2.61798	-1.08718	2.36058
H	2.08790	-1.39254	0.91215
O	1.12322	1.94568	-1.62532
H	1.40614	1.92961	-0.67049
H	1.65659	2.60319	-2.07727
O	-2.29096	-2.10851	0.56805
H	-2.20581	-3.04862	0.73747
H	-1.75483	-1.63795	1.25885
O	2.29127	-2.10818	-0.56803
H	1.75507	-1.63770	-1.25884
H	2.20625	-3.04831	-0.73744
O	-1.59983	1.81958	-0.96846
H	-1.82596	0.91543	-1.25546
H	-0.72760	2.00501	-1.36041
O	-1.92649	-0.86074	-1.73426
H	-2.61782	-1.08757	-2.36058
H	-2.08770	-1.39285	-0.91214
O	0.82625	-0.75048	-2.31917
H	1.00727	0.20002	-2.20034
H	-0.13412	-0.85341	-2.19523
O	1.59956	1.81981	0.96845
H	1.82583	0.91570	1.25545
H	0.72730	2.00512	1.36040

(H2O)10 172
30
Ogolem-172-36523-wb97xd.log

O	-1.71305	1.41539	1.36464
H	-0.82760	1.82100	1.36002
H	-2.02299	1.44849	0.44055
O	2.05740	-0.12695	-1.67301
H	2.44290	0.04728	-0.79939
H	1.46870	0.64303	-1.84242
O	0.36702	2.01863	-1.73849
H	0.52668	2.37127	-0.84903
H	-0.56190	1.72668	-1.73858
O	0.69428	-2.33313	-1.32354
H	1.22181	-1.51542	-1.55659

H	0.99123	-3.04488	-1.89494
O	-1.94766	-1.56056	-0.90910
H	-1.06931	-1.89731	-1.17011
H	-1.97054	-1.61816	0.06195
O	-1.64555	-1.16979	1.87434
H	-2.20749	-1.42153	2.61055
H	-1.69000	-0.17852	1.78321
O	0.89999	2.40260	1.05222
H	1.54674	1.67042	1.17357
H	1.24754	3.17517	1.50363
O	0.99612	-1.89908	1.42034
H	0.08514	-1.70107	1.71014
H	0.91220	-2.25992	0.51816
O	2.50328	0.22181	1.14588
H	3.32177	0.11566	1.63637
H	1.94210	-0.58756	1.32726
O	-2.23514	1.02424	-1.35107
H	-3.00511	1.22735	-1.88687
H	-2.16546	0.03283	-1.28512

3.3 MP2(full)/GTBas1 coordinate files and G3 Energies for all structures

Molecular Formula Structure
H2O Monomer
3
G3-Monomer.log Energy: -47725.8456460

O	-0.00000	0.00000	0.11929
H	0.00000	0.76315	-0.47714
H	-0.00000	-0.76315	-0.47714

G3 Energies at Each Temperature for H2O Monomer

```
Temperature=           200.000000 Pressure=               1.000000
 E(ZPE)=               0.020511 E(Thermal)=              0.022411
 E(QCISD(T))=           -76.207892 E(Empiric)=             -0.025544
 DE(Plus)=             -0.012978 DE(2DF)=               -0.074488
 E(Delta-G3)=           -0.081653 E(G3-Empiric)=           -0.025544
 G3(0 K)=             -76.382044 G3 Energy=              -76.380144
 G3 Enthalpy=           -76.379511 G3 Free Energy=          -76.392837

Temperature=           210.000000 Pressure=               1.000000
 E(ZPE)=               0.020511 E(Thermal)=              0.022506
 E(QCISD(T))=           -76.207892 E(Empiric)=             -0.025544
 DE(Plus)=             -0.012978 DE(2DF)=               -0.074488
 E(Delta-G3)=           -0.081653 E(G3-Empiric)=           -0.025544
 G3(0 K)=             -76.382044 G3 Energy=              -76.380049
 G3 Enthalpy=           -76.379384 G3 Free Energy=          -76.393506

Temperature=           216.650000 Pressure=               1.000000
```

E(ZPE)= 0.020511 E(Thermal)= 0.022569
E(QCISD(T))= -76.207892 E(Empiric)= -0.025544
DE(Plus)= -0.012978 DE(2DF)= -0.074488
E(Delta-G3)= -0.081653 E(G3-Empiric)= -0.025544
G3(0 K)= -76.382044 G3 Energy= -76.379986
G3 Enthalpy= -76.379300 G3 Free Energy= -76.393955

Temperature= 230.000000 Pressure= 1.000000
E(ZPE)= 0.020511 E(Thermal)= 0.022696
E(QCISD(T))= -76.207892 E(Empiric)= -0.025544
DE(Plus)= -0.012978 DE(2DF)= -0.074488
E(Delta-G3)= -0.081653 E(G3-Empiric)= -0.025544
G3(0 K)= -76.382044 G3 Energy= -76.379859
G3 Enthalpy= -76.379131 G3 Free Energy= -76.394863

Temperature= 240.000000 Pressure= 1.000000
E(ZPE)= 0.020511 E(Thermal)= 0.022791
E(QCISD(T))= -76.207892 E(Empiric)= -0.025544
DE(Plus)= -0.012978 DE(2DF)= -0.074488
E(Delta-G3)= -0.081653 E(G3-Empiric)= -0.025544
G3(0 K)= -76.382044 G3 Energy= -76.379764
G3 Enthalpy= -76.379004 G3 Free Energy= -76.395550

Temperature= 250.000000 Pressure= 1.000000
E(ZPE)= 0.020511 E(Thermal)= 0.022887
E(QCISD(T))= -76.207892 E(Empiric)= -0.025544
DE(Plus)= -0.012978 DE(2DF)= -0.074488
E(Delta-G3)= -0.081653 E(G3-Empiric)= -0.025544
G3(0 K)= -76.382044 G3 Energy= -76.379669
G3 Enthalpy= -76.378877 G3 Free Energy= -76.396242

Temperature= 260.000000 Pressure= 1.000000
E(ZPE)= 0.020511 E(Thermal)= 0.022982
E(QCISD(T))= -76.207892 E(Empiric)= -0.025544
DE(Plus)= -0.012978 DE(2DF)= -0.074488
E(Delta-G3)= -0.081653 E(G3-Empiric)= -0.025544
G3(0 K)= -76.382044 G3 Energy= -76.379573
G3 Enthalpy= -76.378750 G3 Free Energy= -76.396939

Temperature= 273.150000 Pressure= 1.000000
E(ZPE)= 0.020511 E(Thermal)= 0.023107
E(QCISD(T))= -76.207892 E(Empiric)= -0.025544
DE(Plus)= -0.012978 DE(2DF)= -0.074488
E(Delta-G3)= -0.081653 E(G3-Empiric)= -0.025544
G3(0 K)= -76.382044 G3 Energy= -76.379448
G3 Enthalpy= -76.378583 G3 Free Energy= -76.397863

Temperature= 298.150000 Pressure= 1.000000
E(ZPE)= 0.020511 E(Thermal)= 0.023346

E(QCISD(T))= -76.207892 E(Empiric)= -0.025544
DE(Plus)= -0.012978 DE(2DF)= -0.074488
E(Delta-G3)= -0.081653 E(G3-Empiric)= -0.025544
G3(0 K)= -76.382044 G3 Energy= -76.379209
G3 Enthalpy= -76.378265 G3 Free Energy= -76.399642

Temperature= 310.000000 Pressure= 1.000000
E(ZPE)= 0.020511 E(Thermal)= 0.023460
E(QCISD(T))= -76.207892 E(Empiric)= -0.025544
DE(Plus)= -0.012978 DE(2DF)= -0.074488
E(Delta-G3)= -0.081653 E(G3-Empiric)= -0.025544
G3(0 K)= -76.382044 G3 Energy= -76.379096
G3 Enthalpy= -76.378114 G3 Free Energy= -76.400494

Temperature= 320.000000 Pressure= 1.000000
E(ZPE)= 0.020511 E(Thermal)= 0.023556
E(QCISD(T))= -76.207892 E(Empiric)= -0.025544
DE(Plus)= -0.012978 DE(2DF)= -0.074488
E(Delta-G3)= -0.081653 E(G3-Empiric)= -0.025544
G3(0 K)= -76.382044 G3 Energy= -76.378999
G3 Enthalpy= -76.377986 G3 Free Energy= -76.401218
Temperature= 330.000000 Pressure= 1.000000
E(ZPE)= 0.020511 E(Thermal)= 0.023652
E(QCISD(T))= -76.207892 E(Empiric)= -0.025544
DE(Plus)= -0.012978 DE(2DF)= -0.074488
E(Delta-G3)= -0.081653 E(G3-Empiric)= -0.025544
G3(0 K)= -76.382044 G3 Energy= -76.378903
G3 Enthalpy= -76.377858 G3 Free Energy= -76.401946

Molecular Formula Structure
(H2O)2 Dimer
6
G3.log Energy: -95455.2061297

O	-1.49459	0.12175	0.00037
H	-1.94156	-0.73632	-0.00223
H	-0.54575	-0.10286	-0.00023
O	1.40815	-0.12702	-0.00039
H	1.58960	0.43834	0.76742
H	1.58928	0.44299	-0.76483

G3 Energies at Each Temperature for (H2O)2 Dimer

```
Temperature=              200.000000 Pressure=                     1.000000
 E(ZPE)=                     0.044117 E(Thermal)=                   0.047759
 E(QCISD(T))=             -152.426941 E(Empiric)=                  -0.051088
 DE(Plus)=                  -0.025086 DE(2DF)=                     -0.148363
 E(Delta-G3)=               -0.161830 E(G3-Empiric)=               -0.051088
 G3(0 K)=                 -152.769190 G3 Energy=                 -152.765548
 G3 Enthalpy=             -152.764915 G3 Free Energy=            -152.785471

Temperature=              210.000000 Pressure=                     1.000000
```

```
 E(ZPE)=                     0.044117 E(Thermal)=                    0.048003
 E(QCISD(T))=             -152.426941 E(Empiric)=                   -0.051088
 DE(Plus)=                  -0.025086 DE(2DF)=                      -0.148363
 E(Delta-G3)=               -0.161830 E(G3-Empiric)=                -0.051088
 G3(0 K)=                 -152.769190 G3 Energy=                  -152.765305
 G3 Enthalpy=             -152.764640 G3 Free Energy=             -152.786506

Temperature=              216.650000 Pressure=                      1.000000
 E(ZPE)=                     0.044117 E(Thermal)=                    0.048166
 E(QCISD(T))=             -152.426941 E(Empiric)=                   -0.051088
 DE(Plus)=                  -0.025086 DE(2DF)=                      -0.148363
 E(Delta-G3)=               -0.161830 E(G3-Empiric)=                -0.051088
 G3(0 K)=                 -152.769190 G3 Energy=                  -152.765141
 G3 Enthalpy=             -152.764455 G3 Free Energy=             -152.787201

Temperature=              230.000000 Pressure=                      1.000000
 E(ZPE)=                     0.044117 E(Thermal)=                    0.048498
 E(QCISD(T))=             -152.426941 E(Empiric)=                   -0.051088
 DE(Plus)=                  -0.025086 DE(2DF)=                      -0.148363
 E(Delta-G3)=               -0.161830 E(G3-Empiric)=                -0.051088
 G3(0 K)=                 -152.769190 G3 Energy=                  -152.764810
 G3 Enthalpy=             -152.764081 G3 Free Energy=             -152.788614

Temperature=              240.000000 Pressure=                      1.000000
 E(ZPE)=                     0.044117 E(Thermal)=                    0.048749
 E(QCISD(T))=             -152.426941 E(Empiric)=                   -0.051088
 DE(Plus)=                  -0.025086 DE(2DF)=                      -0.148363
 E(Delta-G3)=               -0.161830 E(G3-Empiric)=                -0.051088
 G3(0 K)=                 -152.769190 G3 Energy=                  -152.764559
 G3 Enthalpy=             -152.763799 G3 Free Energy=             -152.789687

Temperature=              250.000000 Pressure=                      1.000000
 E(ZPE)=                     0.044117 E(Thermal)=                    0.049002
 E(QCISD(T))=             -152.426941 E(Empiric)=                   -0.051088
 DE(Plus)=                  -0.025086 DE(2DF)=                      -0.148363
 E(Delta-G3)=               -0.161830 E(G3-Empiric)=                -0.051088
 G3(0 K)=                 -152.769190 G3 Energy=                  -152.764306
 G3 Enthalpy=             -152.763514 G3 Free Energy=             -152.790771

Temperature=              260.000000 Pressure=                      1.000000
 E(ZPE)=                     0.044117 E(Thermal)=                    0.049257
 E(QCISD(T))=             -152.426941 E(Empiric)=                   -0.051088
 DE(Plus)=                  -0.025086 DE(2DF)=                      -0.148363
 E(Delta-G3)=               -0.161830 E(G3-Empiric)=                -0.051088
 G3(0 K)=                 -152.769190 G3 Energy=                  -152.764051
 G3 Enthalpy=             -152.763227 G3 Free Energy=             -152.791867

Temperature=              273.150000 Pressure=                      1.000000
 E(ZPE)=                     0.044117 E(Thermal)=                    0.049595
 E(QCISD(T))=             -152.426941 E(Empiric)=                   -0.051088
 DE(Plus)=                  -0.025086 DE(2DF)=                      -0.148363
 E(Delta-G3)=               -0.161830 E(G3-Empiric)=                -0.051088
 G3(0 K)=                 -152.769190 G3 Energy=                  -152.763712
 G3 Enthalpy=             -152.762847 G3 Free Energy=             -152.793325
```

```
Temperature=              298.150000 Pressure=                  1.000000
 E(ZPE)=                     0.044117 E(Thermal)=                 0.050246
 E(QCISD(T))=             -152.426941 E(Empiric)=                -0.051088
 DE(Plus)=                  -0.025086 DE(2DF)=                   -0.148363
 E(Delta-G3)=               -0.161830 E(G3-Empiric)=             -0.051088
 G3(0 K)=                 -152.769190 G3 Energy=               -152.763061
 G3 Enthalpy=             -152.762117 G3 Free Energy=          -152.796147

Temperature=              310.000000 Pressure=                  1.000000
 E(ZPE)=                     0.044117 E(Thermal)=                 0.050558
 E(QCISD(T))=             -152.426941 E(Empiric)=                -0.051088
 DE(Plus)=                  -0.025086 DE(2DF)=                   -0.148363
 E(Delta-G3)=               -0.161830 E(G3-Empiric)=             -0.051088
 G3(0 K)=                 -152.769190 G3 Energy=               -152.762749
 G3 Enthalpy=             -152.761767 G3 Free Energy=          -152.797507

Temperature=              320.000000 Pressure=                  1.000000
 E(ZPE)=                     0.044117 E(Thermal)=                 0.050823
 E(QCISD(T))=             -152.426941 E(Empiric)=                -0.051088
 DE(Plus)=                  -0.025086 DE(2DF)=                   -0.148363
 E(Delta-G3)=               -0.161830 E(G3-Empiric)=             -0.051088
 G3(0 K)=                 -152.769190 G3 Energy=               -152.762484
 G3 Enthalpy=             -152.761471 G3 Free Energy=          -152.798664
```

Molecular Formula	Structure
(H2O)3	Trimer-UUD

```
9
        Energy: -143107.8702988
H          0.57407        1.05566       -0.19783
O          1.46942        0.65008       -0.15427
H          1.87049        1.01553        0.64862
O         -1.31324        0.92343       -0.16297
H         -1.19406       -0.05276       -0.12082
H         -1.79125        1.14647        0.64994
O         -0.15505       -1.60165        0.05880
H          0.63450       -1.01414        0.03503
H         -0.09489       -2.12262       -0.75651
```

G3 Energies at Each Temperature for (H2O)3 Trimer-UUD

```
Temperature=              200.000000 Pressure=                  1.000000
 E(ZPE)=                     0.069970 E(Thermal)=                 0.074331
 E(QCISD(T))=             -228.659504 E(Empiric)=                -0.076632
 DE(Plus)=                  -0.033144 DE(2DF)=                   -0.222094
 E(Delta-G3)=               -0.241514 E(G3-Empiric)=             -0.076632
 G3(0 K)=                 -229.162918 G3 Energy=               -229.158557
 G3 Enthalpy=             -229.157924 G3 Free Energy=          -229.180990

Temperature=              210.000000 Pressure=                  1.000000
 E(ZPE)=                     0.069970 E(Thermal)=                 0.074678
 E(QCISD(T))=             -228.659504 E(Empiric)=                -0.076632
 DE(Plus)=                  -0.033144 DE(2DF)=                   -0.222094
 E(Delta-G3)=               -0.241514 E(G3-Empiric)=             -0.076632
 G3(0 K)=                 -229.162918 G3 Energy=               -229.158210
 G3 Enthalpy=             -229.157545 G3 Free Energy=          -229.182153
```

```
Temperature=              216.650000 Pressure=                    1.000000
 E(ZPE)=                     0.069970 E(Thermal)=                   0.074913
 E(QCISD(T))=              -228.659504 E(Empiric)=                  -0.076632
 DE(Plus)=                   -0.033144 DE(2DF)=                     -0.222094
 E(Delta-G3)=                -0.241514 E(G3-Empiric)=               -0.076632
 G3(0 K)=                  -229.162918 G3 Energy=                 -229.157976
 G3 Enthalpy=              -229.157290 G3 Free Energy=            -229.182936

Temperature=              230.000000 Pressure=                    1.000000
 E(ZPE)=                     0.069970 E(Thermal)=                   0.075392
 E(QCISD(T))=              -228.659504 E(Empiric)=                  -0.076632
 DE(Plus)=                   -0.033144 DE(2DF)=                     -0.222094
 E(Delta-G3)=                -0.241514 E(G3-Empiric)=               -0.076632
 G3(0 K)=                  -229.162918 G3 Energy=                 -229.157496
 G3 Enthalpy=              -229.156767 G3 Free Energy=            -229.184532

Temperature=              240.000000 Pressure=                    1.000000
 E(ZPE)=                     0.069970 E(Thermal)=                   0.075759
 E(QCISD(T))=              -228.659504 E(Empiric)=                  -0.076632
 DE(Plus)=                   -0.033144 DE(2DF)=                     -0.222094
 E(Delta-G3)=                -0.241514 E(G3-Empiric)=               -0.076632
 G3(0 K)=                  -229.162918 G3 Energy=                 -229.157129
 G3 Enthalpy=              -229.156369 G3 Free Energy=            -229.185748

Temperature=              250.000000 Pressure=                    1.000000
 E(ZPE)=                     0.069970 E(Thermal)=                   0.076131
 E(QCISD(T))=              -228.659504 E(Empiric)=                  -0.076632
 DE(Plus)=                   -0.033144 DE(2DF)=                     -0.222094
 E(Delta-G3)=                -0.241514 E(G3-Empiric)=               -0.076632
 G3(0 K)=                  -229.162918 G3 Energy=                 -229.156757
 G3 Enthalpy=              -229.155965 G3 Free Energy=            -229.186980

Temperature=              260.000000 Pressure=                    1.000000
 E(ZPE)=                     0.069970 E(Thermal)=                   0.076509
 E(QCISD(T))=              -228.659504 E(Empiric)=                  -0.076632
 DE(Plus)=                   -0.033144 DE(2DF)=                     -0.222094
 E(Delta-G3)=                -0.241514 E(G3-Empiric)=               -0.076632
 G3(0 K)=                  -229.162918 G3 Energy=                 -229.156379
 G3 Enthalpy=              -229.155556 G3 Free Energy=            -229.188229

Temperature=              273.150000 Pressure=                    1.000000
 E(ZPE)=                     0.069970 E(Thermal)=                   0.077014
 E(QCISD(T))=              -228.659504 E(Empiric)=                  -0.076632
 DE(Plus)=                   -0.033144 DE(2DF)=                     -0.222094
 E(Delta-G3)=                -0.241514 E(G3-Empiric)=               -0.076632
 G3(0 K)=                  -229.162918 G3 Energy=                 -229.155875
 G3 Enthalpy=              -229.155010 G3 Free Energy=            -229.189895

Temperature=              298.150000 Pressure=                    1.000000
 E(ZPE)=                     0.069970 E(Thermal)=                   0.077995
 E(QCISD(T))=              -228.659504 E(Empiric)=                  -0.076632
 DE(Plus)=                   -0.033144 DE(2DF)=                     -0.222094
 E(Delta-G3)=                -0.241514 E(G3-Empiric)=               -0.076632
 G3(0 K)=                  -229.162918 G3 Energy=                 -229.154893
```

```
 G3 Enthalpy=                -229.153949 G3 Free Energy=              -229.193134

Temperature=                 310.000000 Pressure=                       1.000000
 E(ZPE)=                        0.069970 E(Thermal)=                     0.078470
 E(QCISD(T))=                -228.659504 E(Empiric)=                    -0.076632
 DE(Plus)=                     -0.033144 DE(2DF)=                       -0.222094
 E(Delta-G3)=                  -0.241514 E(G3-Empiric)=                 -0.076632
 G3(0 K)=                    -229.162918 G3 Energy=                   -229.154418
 G3 Enthalpy=                -229.153437 G3 Free Energy=              -229.194702

Temperature=                 320.000000 Pressure=                       1.000000
 E(ZPE)=                        0.069970 E(Thermal)=                     0.078875
 E(QCISD(T))=                -228.659504 E(Empiric)=                    -0.076632
 DE(Plus)=                     -0.033144 DE(2DF)=                       -0.222094
 E(Delta-G3)=                  -0.241514 E(G3-Empiric)=                 -0.076632
 G3(0 K)=                    -229.162918 G3 Energy=                   -229.154013
 G3 Enthalpy=                -229.153000 G3 Free Energy=              -229.196040
```

Molecular Formula Structure
(H2O)3 Trimer-UUU

```
9
        Energy: -143106.5453314
H          1.03313        1.87143        0.54671
O          0.67509        1.46796       -0.25790
H         -1.02924        0.64414       -0.28883
H          1.07242        0.56909       -0.28911
O          0.93396       -1.31888       -0.25795
O         -1.60881       -0.14952       -0.25787
H         -2.13764       -0.04144        0.54652
H          1.10423       -1.82922        0.54762
H         -0.04314       -1.21356       -0.28919
```

G3 Energies at Each Temperature for (H2O)3 Trimer-UUU

```
Temperature=                 200.000000 Pressure=                       1.000000
 E(ZPE)=                        0.069288 E(Thermal)=                     0.073943
 E(QCISD(T))=                -228.657043 E(Empiric)=                    -0.076632
 DE(Plus)=                     -0.033927 DE(2DF)=                       -0.222348
 E(Delta-G3)=                  -0.241478 E(G3-Empiric)=                 -0.076632
 G3(0 K)=                    -229.162141 G3 Energy=                   -229.157485
 G3 Enthalpy=                -229.156852 G3 Free Energy=              -229.180666

Temperature=                 210.000000 Pressure=                       1.000000
 E(ZPE)=                        0.069288 E(Thermal)=                     0.074301
 E(QCISD(T))=                -228.657043 E(Empiric)=                    -0.076632
 DE(Plus)=                     -0.033927 DE(2DF)=                       -0.222348
 E(Delta-G3)=                  -0.241478 E(G3-Empiric)=                 -0.076632
 G3(0 K)=                    -229.162141 G3 Energy=                   -229.157127
 G3 Enthalpy=                -229.156462 G3 Free Energy=              -229.181867

Temperature=                 216.650000 Pressure=                       1.000000
 E(ZPE)=                        0.069288 E(Thermal)=                     0.074543
 E(QCISD(T))=                -228.657043 E(Empiric)=                    -0.076632
 DE(Plus)=                     -0.033927 DE(2DF)=                       -0.222348
 E(Delta-G3)=                  -0.241478 E(G3-Empiric)=                 -0.076632
```

```
 G3(0 K)=                    -229.162141 G3 Energy=                    -229.156885
 G3 Enthalpy=                -229.156199 G3 Free Energy=               -229.182675

Temperature=                 230.000000 Pressure=                        1.000000
 E(ZPE)=                        0.069288 E(Thermal)=                      0.075038
 E(QCISD(T))=                -228.657043 E(Empiric)=                     -0.076632
 DE(Plus)=                     -0.033927 DE(2DF)=                        -0.222348
 E(Delta-G3)=                  -0.241478 E(G3-Empiric)=                  -0.076632
 G3(0 K)=                    -229.162141 G3 Energy=                    -229.156390
 G3 Enthalpy=                -229.155662 G3 Free Energy=               -229.184323

Temperature=                 240.000000 Pressure=                        1.000000
 E(ZPE)=                        0.069288 E(Thermal)=                      0.075415
 E(QCISD(T))=                -228.657043 E(Empiric)=                     -0.076632
 DE(Plus)=                     -0.033927 DE(2DF)=                        -0.222348
 E(Delta-G3)=                  -0.241478 E(G3-Empiric)=                  -0.076632
 G3(0 K)=                    -229.162141 G3 Energy=                    -229.156013
 G3 Enthalpy=                -229.155253 G3 Free Energy=               -229.185578

Temperature=                 250.000000 Pressure=                        1.000000
 E(ZPE)=                        0.069288 E(Thermal)=                      0.075798
 E(QCISD(T))=                -228.657043 E(Empiric)=                     -0.076632
 DE(Plus)=                     -0.033927 DE(2DF)=                        -0.222348
 E(Delta-G3)=                  -0.241478 E(G3-Empiric)=                  -0.076632
 G3(0 K)=                    -229.162141 G3 Energy=                    -229.155631
 G3 Enthalpy=                -229.154839 G3 Free Energy=               -229.186850

 Temperature=                 260.000000 Pressure=                        1.000000
 E(ZPE)=                        0.069288 E(Thermal)=                      0.076185
 E(QCISD(T))=                -228.657043 E(Empiric)=                     -0.076632
 DE(Plus)=                     -0.033927 DE(2DF)=                        -0.222348
 E(Delta-G3)=                  -0.241478 E(G3-Empiric)=                  -0.076632
 G3(0 K)=                    -229.162141 G3 Energy=                    -229.155243
 G3 Enthalpy=                -229.154419 G3 Free Energy=               -229.188139

Temperature=                 273.150000 Pressure=                        1.000000
 E(ZPE)=                        0.069288 E(Thermal)=                      0.076703
 E(QCISD(T))=                -228.657043 E(Empiric)=                     -0.076632
 DE(Plus)=                     -0.033927 DE(2DF)=                        -0.222348
 E(Delta-G3)=                  -0.241478 E(G3-Empiric)=                  -0.076632
 G3(0 K)=                    -229.162141 G3 Energy=                    -229.154726
 G3 Enthalpy=                -229.153861 G3 Free Energy=               -229.189858

Temperature=                 298.150000 Pressure=                        1.000000
 E(ZPE)=                        0.069288 E(Thermal)=                      0.077707
 E(QCISD(T))=                -228.657043 E(Empiric)=                     -0.076632
 DE(Plus)=                     -0.033927 DE(2DF)=                        -0.222348
 E(Delta-G3)=                  -0.241478 E(G3-Empiric)=                  -0.076632
 G3(0 K)=                    -229.162141 G3 Energy=                    -229.153721
 G3 Enthalpy=                -229.152777 G3 Free Energy=               -229.193200

Temperature=                 310.000000 Pressure=                        1.000000
 E(ZPE)=                        0.069288 E(Thermal)=                      0.078192
 E(QCISD(T))=                -228.657043 E(Empiric)=                     -0.076632
 DE(Plus)=                     -0.033927 DE(2DF)=                        -0.222348
```

```
 E(Delta-G3)=                 -0.241478 E(G3-Empiric)=              -0.076632
 G3(0 K)=                   -229.162141 G3 Energy=                -229.153237
 G3 Enthalpy=               -229.152255 G3 Free Energy=           -229.194817

Temperature=                320.000000 Pressure=                    1.000000
 E(ZPE)=                       0.069288 E(Thermal)=                  0.078605
 E(QCISD(T))=               -228.657043 E(Empiric)=                 -0.076632
 DE(Plus)=                    -0.033927 DE(2DF)=                    -0.222348
 E(Delta-G3)=                 -0.241478 E(G3-Empiric)=              -0.076632
 G3(0 K)=                   -229.162141 G3 Energy=                -229.152824
 G3 Enthalpy=               -229.151810 G3 Free Energy=           -229.196197
```

Molecular Formula Structure
(H2O)4 Tetramer

```
12
G3_92.log       Energy: -190921.0286197
O         -1.94230       -0.02089        0.03655
H         -1.32274       -0.79378        0.04718
H         -2.45202       -0.12949       -0.78060
O         -0.02089        1.94228       -0.03650
H         -0.12952        2.45208        0.78060
H         -0.79378        1.32273       -0.04714
O          0.02089       -1.94228       -0.03660
H          0.79378       -1.32273       -0.04723
H          0.12950       -2.45203        0.78053
O          1.94230        0.02089        0.03655
H          2.45203        0.12953       -0.78059
H          1.32275        0.79378        0.04722
```

G3 Energies at Each Temperature for (H2O)4 Tetramer

```
Temperature=                200.000000 Pressure=                    1.000000
 E(ZPE)=                       0.094636 E(Thermal)=                  0.100381
 E(QCISD(T))=               -304.891071 E(Empiric)=                 -0.102176
 DE(Plus)=                    -0.046056 DE(2DF)=                    -0.295338
 E(Delta-G3)=                 -0.320540 E(G3-Empiric)=              -0.102176
 G3(0 K)=                   -305.560545 G3 Energy=                -305.554800
 G3 Enthalpy=               -305.554167 G3 Free Energy=           -305.580981

Temperature=                210.000000 Pressure=                    1.000000
 E(ZPE)=                       0.094636 E(Thermal)=                  0.100848
 E(QCISD(T))=               -304.891071 E(Empiric)=                 -0.102176
 DE(Plus)=                    -0.046056 DE(2DF)=                    -0.295338
 E(Delta-G3)=                 -0.320540 E(G3-Empiric)=              -0.102176
 G3(0 K)=                   -305.560545 G3 Energy=                -305.554333
 G3 Enthalpy=               -305.553668 G3 Free Energy=           -305.582334

Temperature=                216.650000 Pressure=                    1.000000
 E(ZPE)=                       0.094636 E(Thermal)=                  0.101164
 E(QCISD(T))=               -304.891071 E(Empiric)=                 -0.102176
 DE(Plus)=                    -0.046056 DE(2DF)=                    -0.295338
 E(Delta-G3)=                 -0.320540 E(G3-Empiric)=              -0.102176
 G3(0 K)=                   -305.560545 G3 Energy=                -305.554017
 G3 Enthalpy=               -305.553331 G3 Free Energy=           -305.583247
```

```
Temperature=             230.000000 Pressure=                  1.000000
 E(ZPE)=                    0.094636 E(Thermal)=                 0.101811
 E(QCISD(T))=            -304.891071 E(Empiric)=                -0.102176
 DE(Plus)=                 -0.046056 DE(2DF)=                   -0.295338
 E(Delta-G3)=              -0.320540 E(G3-Empiric)=             -0.102176
 G3(0 K)=                -305.560545 G3 Energy=               -305.553369
 G3 Enthalpy=            -305.552641 G3 Free Energy=          -305.585111

Temperature=             240.000000 Pressure=                  1.000000
 E(ZPE)=                    0.094636 E(Thermal)=                 0.102307
 E(QCISD(T))=            -304.891071 E(Empiric)=                -0.102176
 DE(Plus)=                 -0.046056 DE(2DF)=                   -0.295338
 E(Delta-G3)=              -0.320540 E(G3-Empiric)=             -0.102176
 G3(0 K)=                -305.560545 G3 Energy=               -305.552874
 G3 Enthalpy=            -305.552114 G3 Free Energy=          -305.586534

Temperature=             250.000000 Pressure=                  1.000000
 E(ZPE)=                    0.094636 E(Thermal)=                 0.102811
 E(QCISD(T))=            -304.891071 E(Empiric)=                -0.102176
 DE(Plus)=                 -0.046056 DE(2DF)=                   -0.295338
 E(Delta-G3)=              -0.320540 E(G3-Empiric)=             -0.102176
 G3(0 K)=                -305.560545 G3 Energy=               -305.552370
 G3 Enthalpy=            -305.551578 G3 Free Energy=          -305.587979

Temperature=             260.000000 Pressure=                  1.000000
 E(ZPE)=                    0.094636 E(Thermal)=                 0.103323
 E(QCISD(T))=            -304.891071 E(Empiric)=                -0.102176
 DE(Plus)=                 -0.046056 DE(2DF)=                   -0.295338
 E(Delta-G3)=              -0.320540 E(G3-Empiric)=             -0.102176
 G3(0 K)=                -305.560545 G3 Energy=               -305.551858
 G3 Enthalpy=            -305.551035 G3 Free Energy=          -305.589446

Temperature=             273.150000 Pressure=                  1.000000
 E(ZPE)=                    0.094636 E(Thermal)=                 0.104007
 E(QCISD(T))=            -304.891071 E(Empiric)=                -0.102176
 DE(Plus)=                 -0.046056 DE(2DF)=                   -0.295338
 E(Delta-G3)=              -0.320540 E(G3-Empiric)=             -0.102176
 G3(0 K)=                -305.560545 G3 Energy=               -305.551173
 G3 Enthalpy=            -305.550308 G3 Free Energy=          -305.591406

Temperature=             298.150000 Pressure=                  1.000000
 E(ZPE)=                    0.094636 E(Thermal)=                 0.105342
 E(QCISD(T))=            -304.891071 E(Empiric)=                -0.102176
 DE(Plus)=                 -0.046056 DE(2DF)=                   -0.295338
 E(Delta-G3)=              -0.320540 E(G3-Empiric)=             -0.102176
 G3(0 K)=                -305.560545 G3 Energy=               -305.549839
 G3 Enthalpy=            -305.548895 G3 Free Energy=          -305.595230

Temperature=             310.000000 Pressure=                  1.000000
 E(ZPE)=                    0.094636 E(Thermal)=                 0.105989
 E(QCISD(T))=            -304.891071 E(Empiric)=                -0.102176
 DE(Plus)=                 -0.046056 DE(2DF)=                   -0.295338
 E(Delta-G3)=              -0.320540 E(G3-Empiric)=             -0.102176
 G3(0 K)=                -305.560545 G3 Energy=               -305.549192
 G3 Enthalpy=            -305.548211 G3 Free Energy=          -305.597085
```

```
Temperature=                320.000000 Pressure=                    1.000000
 E(ZPE)=                       0.094636 E(Thermal)=                    0.106541
 E(QCISD(T))=               -304.891071 E(Empiric)=                   -0.102176
 DE(Plus)=                    -0.046056 DE(2DF)=                      -0.295338
 E(Delta-G3)=                 -0.320540 E(G3-Empiric)=                -0.102176
 G3(0 K)=                   -305.560545 G3 Energy=                  -305.548640
 G3 Enthalpy=               -305.547627 G3 Free Energy=             -305.598671
```

Molecular Formula　　Structure
(H2O)5　　Pentamer

```
15
        Energy: -238522.1587958
H          0.95770        1.68259       -0.00684
H         -2.59851        1.22017        0.76685
O          0.19661        2.31755       -0.02210
O          2.27843        0.53995       -0.06607
H          1.93467       -0.38778       -0.11634
H          2.81570        0.56318        0.73951
O          1.19640       -1.98700       -0.12391
H          1.40255       -2.58622        0.60859
H          0.20850       -1.90782       -0.10502
O         -1.52935       -1.75237        0.01403
H         -1.77351       -0.79153        0.03497
H         -1.95628       -2.08872       -0.78834
O         -2.15186        0.91205       -0.03589
H         -1.31059        1.43609       -0.06559
H          0.32953        2.82987       -0.83384
```

G3 Energies at Each Temperature for (H2O)5 Pentamer

```
Temperature=                200.000000 Pressure=                    1.000000
 E(ZPE)=                       0.118064 E(Thermal)=                    0.125765
 E(QCISD(T))=               -381.115485 E(Empiric)=                   -0.127720
 DE(Plus)=                    -0.059965 DE(2DF)=                      -0.368653
 E(Delta-G3)=                 -0.400332 E(G3-Empiric)=                -0.127720
 G3(0 K)=                   -381.954091 G3 Energy=                  -381.946390
 G3 Enthalpy=               -381.945757 G3 Free Energy=             -381.978197

Temperature=                210.000000 Pressure=                    1.000000
 E(ZPE)=                       0.118064 E(Thermal)=                    0.126374
 E(QCISD(T))=               -381.115485 E(Empiric)=                   -0.127720
 DE(Plus)=                    -0.059965 DE(2DF)=                      -0.368653
 E(Delta-G3)=                 -0.400332 E(G3-Empiric)=                -0.127720
 G3(0 K)=                   -381.954091 G3 Energy=                  -381.945781
 G3 Enthalpy=               -381.945116 G3 Free Energy=             -381.979835

Temperature=                216.650000 Pressure=                    1.000000
 E(ZPE)=                       0.118064 E(Thermal)=                    0.126787
 E(QCISD(T))=               -381.115485 E(Empiric)=                   -0.127720
 DE(Plus)=                    -0.059965 DE(2DF)=                      -0.368653
 E(Delta-G3)=                 -0.400332 E(G3-Empiric)=                -0.127720
 G3(0 K)=                   -381.954091 G3 Energy=                  -381.945368
 G3 Enthalpy=               -381.944682 G3 Free Energy=             -381.980941
```

```
Temperature=             230.000000 Pressure=                  1.000000
 E(ZPE)=                    0.118064 E(Thermal)=                 0.127630
 E(QCISD(T))=            -381.115485 E(Empiric)=                -0.127720
 DE(Plus)=                 -0.059965 DE(2DF)=                   -0.368653
 E(Delta-G3)=              -0.400332 E(G3-Empiric)=             -0.127720
 G3(0 K)=                -381.954091 G3 Energy=               -381.944525
 G3 Enthalpy=            -381.943796 G3 Free Energy=          -381.983202

Temperature=             240.000000 Pressure=                  1.000000
 E(ZPE)=                    0.118064 E(Thermal)=                 0.128275
 E(QCISD(T))=            -381.115485 E(Empiric)=                -0.127720
 DE(Plus)=                 -0.059965 DE(2DF)=                   -0.368653
 E(Delta-G3)=              -0.400332 E(G3-Empiric)=             -0.127720
 G3(0 K)=                -381.954091 G3 Energy=               -381.943880
 G3 Enthalpy=            -381.943120 G3 Free Energy=          -381.984930

Temperature=             250.000000 Pressure=                  1.000000
 E(ZPE)=                    0.118064 E(Thermal)=                 0.128931
 E(QCISD(T))=            -381.115485 E(Empiric)=                -0.127720
 DE(Plus)=                 -0.059965 DE(2DF)=                   -0.368653
 E(Delta-G3)=              -0.400332 E(G3-Empiric)=             -0.127720
 G3(0 K)=                -381.954091 G3 Energy=               -381.943224
 G3 Enthalpy=            -381.942432 G3 Free Energy=          -381.986686

Temperature=             260.000000 Pressure=                  1.000000
 E(ZPE)=                    0.118064 E(Thermal)=                 0.129596
 E(QCISD(T))=            -381.115485 E(Empiric)=                -0.127720
 DE(Plus)=                 -0.059965 DE(2DF)=                   -0.368653
 E(Delta-G3)=              -0.400332 E(G3-Empiric)=             -0.127720
 G3(0 K)=                -381.954091 G3 Energy=               -381.942559
 G3 Enthalpy=            -381.941735 G3 Free Energy=          -381.988470

Temperature=             273.150000 Pressure=                  1.000000
 E(ZPE)=                    0.118064 E(Thermal)=                 0.130486
 E(QCISD(T))=            -381.115485 E(Empiric)=                -0.127720
 DE(Plus)=                 -0.059965 DE(2DF)=                   -0.368653
 E(Delta-G3)=              -0.400332 E(G3-Empiric)=             -0.127720
 G3(0 K)=                -381.954091 G3 Energy=               -381.941669
 G3 Enthalpy=            -381.940804 G3 Free Energy=          -381.990856

Temperature=             298.150000 Pressure=                  1.000000
 E(ZPE)=                    0.118064 E(Thermal)=                 0.132219
 E(QCISD(T))=            -381.115485 E(Empiric)=                -0.127720
 DE(Plus)=                 -0.059965 DE(2DF)=                   -0.368653
 E(Delta-G3)=              -0.400332 E(G3-Empiric)=             -0.127720
 G3(0 K)=                -381.954091 G3 Energy=               -381.939936
 G3 Enthalpy=            -381.938992 G3 Free Energy=          -381.995517

Temperature=             310.000000 Pressure=                  1.000000
 E(ZPE)=                    0.118064 E(Thermal)=                 0.133057
 E(QCISD(T))=            -381.115485 E(Empiric)=                -0.127720
 DE(Plus)=                 -0.059965 DE(2DF)=                   -0.368653
 E(Delta-G3)=              -0.400332 E(G3-Empiric)=             -0.127720
 G3(0 K)=                -381.954091 G3 Energy=               -381.939098
 G3 Enthalpy=            -381.938116 G3 Free Energy=          -381.997781
```

```
Temperature=                320.000000 Pressure=                     1.000000
 E(ZPE)=                       0.118064 E(Thermal)=                    0.133774
 E(QCISD(T))=               -381.115485 E(Empiric)=                   -0.127720
 DE(Plus)=                    -0.059965 DE(2DF)=                      -0.368653
 E(Delta-G3)=                 -0.400332 E(G3-Empiric)=                -0.127720
 G3(0 K)=                   -381.954091 G3 Energy=                  -381.938381
 G3 Enthalpy=               -381.937368 G3 Free Energy=             -381.999718
```

Molecular Formula	Structure
(H2O)6	Hexamer

```
18
        Energy: -286227.0179945
O          2.45549         1.28670        -0.39329
H          2.38207         1.70505        -1.26417
H          1.58834         1.45203         0.04120
O         -2.32556        -1.46814        -0.47347
H         -2.95568        -1.94383         0.08856
H         -1.44936        -1.57502        -0.01009
O         -2.40504         1.23492        -0.45226
H         -2.49939         0.24326        -0.45925
H         -2.43399         1.48611        -1.38801
O         -0.01867        -1.43737         0.93856
H          0.02205        -0.47569         1.11068
H          0.79916        -1.61122         0.41697
O         -0.05100         1.45441         0.83733
H         -0.90332         1.45599         0.31745
H         -0.22139         2.01068         1.61299
O          2.39378        -1.49686        -0.48593
H          2.50775        -0.51781        -0.52192
H          3.11475        -1.80322         0.08463
```

G3 Energies at Each Temperature for (H2O)6 Hexamer

```
Temperature=                200.000000 Pressure=                     1.000000
 E(ZPE)=                       0.141511 E(Thermal)=                    0.151175
 E(QCISD(T))=               -457.345641 E(Empiric)=                   -0.153264
 DE(Plus)=                    -0.068822 DE(2DF)=                      -0.442990
 E(Delta-G3)=                 -0.479595 E(G3-Empiric)=                -0.153264
 G3(0 K)=                   -458.348801 G3 Energy=                  -458.339137
 G3 Enthalpy=               -458.338504 G3 Free Energy=             -458.376188

Temperature=                210.000000 Pressure=                     1.000000
 E(ZPE)=                       0.141511 E(Thermal)=                    0.151931
 E(QCISD(T))=               -457.345641 E(Empiric)=                   -0.153264
 DE(Plus)=                    -0.068822 DE(2DF)=                      -0.442990
 E(Delta-G3)=                 -0.479595 E(G3-Empiric)=                -0.153264
 G3(0 K)=                   -458.348801 G3 Energy=                  -458.338381
 G3 Enthalpy=               -458.337716 G3 Free Energy=             -458.378091

Temperature=                216.650000 Pressure=                     1.000000
 E(ZPE)=                       0.141511 E(Thermal)=                    0.152441
 E(QCISD(T))=               -457.345641 E(Empiric)=                   -0.153264
 DE(Plus)=                    -0.068822 DE(2DF)=                      -0.442990
 E(Delta-G3)=                 -0.479595 E(G3-Empiric)=                -0.153264
 G3(0 K)=                   -458.348801 G3 Energy=                  -458.337871
```

```
 G3 Enthalpy=                -458.337185 G3 Free Energy=             -458.379378

Temperature=                 230.000000 Pressure=                       1.000000
 E(ZPE)=                        0.141511 E(Thermal)=                    0.153486
 E(QCISD(T))=                -457.345641 E(Empiric)=                   -0.153264
 DE(Plus)=                     -0.068822 DE(2DF)=                      -0.442990
 E(Delta-G3)=                  -0.479595 E(G3-Empiric)=                -0.153264
 G3(0 K)=                    -458.348801 G3 Energy=                  -458.336826
 G3 Enthalpy=                -458.336098 G3 Free Energy=             -458.382011

Temperature=                 240.000000 Pressure=                       1.000000
 E(ZPE)=                        0.141511 E(Thermal)=                    0.154283
 E(QCISD(T))=                -457.345641 E(Empiric)=                   -0.153264
 DE(Plus)=                     -0.068822 DE(2DF)=                      -0.442990
 E(Delta-G3)=                  -0.479595 E(G3-Empiric)=                -0.153264
 G3(0 K)=                    -458.348801 G3 Energy=                  -458.336029
 G3 Enthalpy=                -458.335269 G3 Free Energy=             -458.384025

Temperature=                 250.000000 Pressure=                       1.000000
 E(ZPE)=                        0.141511 E(Thermal)=                    0.155094
 E(QCISD(T))=                -457.345641 E(Empiric)=                   -0.153264
 DE(Plus)=                     -0.068822 DE(2DF)=                      -0.442990
 E(Delta-G3)=                  -0.479595 E(G3-Empiric)=                -0.153264
 G3(0 K)=                    -458.348801 G3 Energy=                  -458.335218
 G3 Enthalpy=                -458.334427 G3 Free Energy=             -458.386073

Temperature=                 260.000000 Pressure=                       1.000000
 E(ZPE)=                        0.141511 E(Thermal)=                    0.155916
 E(QCISD(T))=                -457.345641 E(Empiric)=                   -0.153264
 DE(Plus)=                     -0.068822 DE(2DF)=                      -0.442990
 E(Delta-G3)=                  -0.479595 E(G3-Empiric)=                -0.153264
 G3(0 K)=                    -458.348801 G3 Energy=                  -458.334396
 G3 Enthalpy=                -458.333573 G3 Free Energy=             -458.388156

Temperature=                 273.150000 Pressure=                       1.000000
 E(ZPE)=                        0.141511 E(Thermal)=                    0.157013
 E(QCISD(T))=                -457.345641 E(Empiric)=                   -0.153264
 DE(Plus)=                     -0.068822 DE(2DF)=                      -0.442990
 E(Delta-G3)=                  -0.479595 E(G3-Empiric)=                -0.153264
 G3(0 K)=                    -458.348801 G3 Energy=                  -458.333299
 G3 Enthalpy=                -458.332434 G3 Free Energy=             -458.390945

Temperature=                 298.150000 Pressure=                       1.000000
 E(ZPE)=                        0.141511 E(Thermal)=                    0.159150
 E(QCISD(T))=                -457.345641 E(Empiric)=                   -0.153264
 DE(Plus)=                     -0.068822 DE(2DF)=                      -0.442990
 E(Delta-G3)=                  -0.479595 E(G3-Empiric)=                -0.153264
 G3(0 K)=                    -458.348801 G3 Energy=                  -458.331162
 G3 Enthalpy=                -458.330218 G3 Free Energy=             -458.396398

Temperature=                 310.000000 Pressure=                       1.000000
 E(ZPE)=                        0.141511 E(Thermal)=                    0.160183
 E(QCISD(T))=                -457.345641 E(Empiric)=                   -0.153264
 DE(Plus)=                     -0.068822 DE(2DF)=                      -0.442990
 E(Delta-G3)=                  -0.479595 E(G3-Empiric)=                -0.153264
 G3(0 K)=                    -458.348801 G3 Energy=                  -458.330129
```

```
 G3 Enthalpy=               -458.329147 G3 Free Energy=             -458.399049

Temperature=                320.000000 Pressure=                    1.000000
 E(ZPE)=                       0.141511 E(Thermal)=                  0.161064
 E(QCISD(T))=               -457.345641 E(Empiric)=                 -0.153264
 DE(Plus)=                    -0.068822 DE(2DF)=                    -0.442990
 E(Delta-G3)=                 -0.479595 E(G3-Empiric)=              -0.153264
 G3(0 K)=                   -458.348801 G3 Energy=                -458.329248
 G3 Enthalpy=               -458.328234 G3 Free Energy=             -458.401319
```

Molecular Formula Structure
(H2O)7 Heptamer

```
21
        Energy: -333933.9004313
O          2.84423        0.31164        0.62585
H          2.19411        1.01218        0.33021
H          3.63349        0.47757        0.08769
O          0.05318       -0.10026       -2.06251
H          0.50197       -0.82565       -1.58435
H         -0.88341       -0.18312       -1.79304
O          1.30787       -1.76938       -0.09385
H          1.79497       -2.60732       -0.07299
H          1.95597       -1.06561        0.19760
O          0.96226        1.94270       -0.38652
H          0.65941        1.34376       -1.11133
H          0.20414        1.94731        0.23145
O         -2.52796       -0.18317       -0.77096
H         -2.33562        0.56671       -0.16392
H         -3.36134        0.03335       -1.21621
O         -1.02089       -1.33548        1.40203
H         -0.17358       -1.58576        0.96740
H         -1.68695       -1.43270        0.69640
O         -1.33922        1.32469        1.21932
H         -1.67920        1.74087        2.02649
H         -1.10342        0.38771        1.47125
```

G3 Energies at Each Temperature for (H2O)7 Heptamer

```
Temperature=                200.000000 Pressure=                    1.000000
 E(ZPE)=                       0.166733 E(Thermal)=                  0.177534
 E(QCISD(T))=               -533.579405 E(Empiric)=                 -0.178808
 DE(Plus)=                    -0.077020 DE(2DF)=                    -0.517576
 E(Delta-G3)=                 -0.558415 E(G3-Empiric)=              -0.178808
 G3(0 K)=                   -534.744490 G3 Energy=                -534.733690
 G3 Enthalpy=               -534.733057 G3 Free Energy=             -534.773350

Temperature=                210.000000 Pressure=                    1.000000
 E(ZPE)=                       0.166733 E(Thermal)=                  0.178399
 E(QCISD(T))=               -533.579405 E(Empiric)=                 -0.178808
 DE(Plus)=                    -0.077020 DE(2DF)=                    -0.517576
 E(Delta-G3)=                 -0.558415 E(G3-Empiric)=              -0.178808
 G3(0 K)=                   -534.744490 G3 Energy=                -534.732825
 G3 Enthalpy=               -534.732160 G3 Free Energy=             -534.775387

Temperature=                216.650000 Pressure=                    1.000000
```

```
 E(ZPE)=                        0.166733 E(Thermal)=                     0.178985
 E(QCISD(T))=                -533.579405 E(Empiric)=                    -0.178808
 DE(Plus)=                     -0.077020 DE(2DF)=                       -0.517576
 E(Delta-G3)=                  -0.558415 E(G3-Empiric)=                 -0.178808
 G3(0 K)=                    -534.744490 G3 Energy=                   -534.732239
 G3 Enthalpy=                -534.731553 G3 Free Energy=              -534.776765

Temperature=                 230.000000 Pressure=                       1.000000
 E(ZPE)=                        0.166733 E(Thermal)=                     0.180184
 E(QCISD(T))=                -533.579405 E(Empiric)=                    -0.178808
 DE(Plus)=                     -0.077020 DE(2DF)=                       -0.517576
 E(Delta-G3)=                  -0.558415 E(G3-Empiric)=                 -0.178808
 G3(0 K)=                    -534.744490 G3 Energy=                   -534.731039
 G3 Enthalpy=                -534.730311 G3 Free Energy=              -534.779589

Temperature=                 240.000000 Pressure=                       1.000000
 E(ZPE)=                        0.166733 E(Thermal)=                     0.181103
 E(QCISD(T))=                -533.579405 E(Empiric)=                    -0.178808
 DE(Plus)=                     -0.077020 DE(2DF)=                       -0.517576
 E(Delta-G3)=                  -0.558415 E(G3-Empiric)=                 -0.178808
 G3(0 K)=                    -534.744490 G3 Energy=                   -534.730121
 G3 Enthalpy=                -534.729361 G3 Free Energy=              -534.781752

Temperature=                 250.000000 Pressure=                       1.000000
 E(ZPE)=                        0.166733 E(Thermal)=                     0.182037
 E(QCISD(T))=                -533.579405 E(Empiric)=                    -0.178808
 DE(Plus)=                     -0.077020 DE(2DF)=                       -0.517576
 E(Delta-G3)=                  -0.558415 E(G3-Empiric)=                 -0.178808
 G3(0 K)=                    -534.744490 G3 Energy=                   -534.729186
 G3 Enthalpy=                -534.728395 G3 Free Energy=              -534.783954

Temperature=                 260.000000 Pressure=                       1.000000
 E(ZPE)=                        0.166733 E(Thermal)=                     0.182987
 E(QCISD(T))=                -533.579405 E(Empiric)=                    -0.178808
 DE(Plus)=                     -0.077020 DE(2DF)=                       -0.517576
 E(Delta-G3)=                  -0.558415 E(G3-Empiric)=                 -0.178808
 G3(0 K)=                    -534.744490 G3 Energy=                   -534.728237
 G3 Enthalpy=                -534.727414 G3 Free Energy=              -534.786196

Temperature=                 273.150000 Pressure=                       1.000000
 E(ZPE)=                        0.166733 E(Thermal)=                     0.184257
 E(QCISD(T))=                -533.579405 E(Empiric)=                    -0.178808
 DE(Plus)=                     -0.077020 DE(2DF)=                       -0.517576
 E(Delta-G3)=                  -0.558415 E(G3-Empiric)=                 -0.178808
 G3(0 K)=                    -534.744490 G3 Energy=                   -534.726967
 G3 Enthalpy=                -534.726102 G3 Free Energy=              -534.789202

Temperature=                 298.150000 Pressure=                       1.000000
 E(ZPE)=                        0.166733 E(Thermal)=                     0.186733
 E(QCISD(T))=                -533.579405 E(Empiric)=                    -0.178808
 DE(Plus)=                     -0.077020 DE(2DF)=                       -0.517576
 E(Delta-G3)=                  -0.558415 E(G3-Empiric)=                 -0.178808
 G3(0 K)=                    -534.744490 G3 Energy=                   -534.724491
 G3 Enthalpy=                -534.723546 G3 Free Energy=              -534.795090

Temperature=                 310.000000 Pressure=                       1.000000
```

```
 E(ZPE)=                        0.166733 E(Thermal)=                        0.187933
 E(QCISD(T))=                -533.579405 E(Empiric)=                       -0.178808
 DE(Plus)=                     -0.077020 DE(2DF)=                          -0.517576
 E(Delta-G3)=                  -0.558415 E(G3-Empiric)=                    -0.178808
 G3(0 K)=                    -534.744490 G3 Energy=                      -534.723290
 G3 Enthalpy=                -534.722309 G3 Free Energy=                 -534.797957

Temperature=                 320.000000 Pressure=                         1.000000
 E(ZPE)=                        0.166733 E(Thermal)=                        0.188958
 E(QCISD(T))=                -533.579405 E(Empiric)=                       -0.178808
 DE(Plus)=                     -0.077020 DE(2DF)=                          -0.517576
 E(Delta-G3)=                  -0.558415 E(G3-Empiric)=                    -0.178808
 G3(0 K)=                    -534.744490 G3 Energy=                      -534.722265
 G3 Enthalpy=                -534.721252 G3 Free Energy=                 -534.800415
```

Molecular Formula Structure
(H2O)8 Octamer

```
24
        Energy: -381640.0585289
O          0.91833       -1.74285       -0.77539
H          0.68604       -0.86138       -1.19518
H          0.75688       -2.41250       -1.45816
O          3.30558       -0.87423        0.30840
H          3.93979       -0.89431       -0.42478
H          2.52447       -1.37453       -0.02851
O          2.02647        1.53210        0.47537
H          2.57237        0.70309        0.50934
H          2.60798        2.24951        0.76988
O         -3.20241       -0.94032       -0.20290
H         -2.44377       -1.29599        0.34630
H         -3.95668       -0.94072        0.40637
O          0.44973        0.71615       -1.72338
H          1.03355        1.21942       -1.11877
H         -0.45805        0.99994       -1.47167
O         -1.07395       -1.58122        1.29635
H         -0.35260       -1.79042        0.66866
H         -0.84681       -0.67670        1.62283
O         -2.04159        1.44012       -0.65015
H         -2.64434        2.07043       -1.07308
H         -2.56127        0.59402       -0.53446
O         -0.52495        1.11455        1.76215
H          0.38311        1.29233        1.44410
H         -1.09787        1.45351        1.04667
```

G3 Energies at Each Temperature for (H2O)8 Octamer

```
Temperature=                 200.000000 Pressure=                         1.000000
 E(ZPE)=                        0.190407 E(Thermal)=                        0.203100
 E(QCISD(T))=                -609.809288 E(Empiric)=                       -0.204352
 DE(Plus)=                     -0.089735 DE(2DF)=                          -0.591426
 E(Delta-G3)=                  -0.637178 E(G3-Empiric)=                    -0.204352
 G3(0 K)=                    -611.141571 G3 Energy=                      -611.128878
 G3 Enthalpy=                -611.128245 G3 Free Energy=                 -611.174042

Temperature=                 210.000000 Pressure=                         1.000000
```

```
 E(ZPE)=                      0.190407 E(Thermal)=                   0.204105
 E(QCISD(T))=              -609.809288 E(Empiric)=                  -0.204352
 DE(Plus)=                   -0.089735 DE(2DF)=                     -0.591426
 E(Delta-G3)=                -0.637178 E(G3-Empiric)=               -0.204352
 G3(0 K)=                  -611.141571 G3 Energy=                 -611.127874
 G3 Enthalpy=              -611.127209 G3 Free Energy=            -611.176357

Temperature=               216.650000 Pressure=                   1.000000
 E(ZPE)=                      0.190407 E(Thermal)=                   0.204785
 E(QCISD(T))=              -609.809288 E(Empiric)=                  -0.204352
 DE(Plus)=                   -0.089735 DE(2DF)=                     -0.591426
 E(Delta-G3)=                -0.637178 E(G3-Empiric)=               -0.204352
 G3(0 K)=                  -611.141571 G3 Energy=                 -611.127194
 G3 Enthalpy=              -611.126508 G3 Free Energy=            -611.177925

Temperature=               230.000000 Pressure=                   1.000000
 E(ZPE)=                      0.190407 E(Thermal)=                   0.206177
 E(QCISD(T))=              -609.809288 E(Empiric)=                  -0.204352
 DE(Plus)=                   -0.089735 DE(2DF)=                     -0.591426
 E(Delta-G3)=                -0.637178 E(G3-Empiric)=               -0.204352
 G3(0 K)=                  -611.141571 G3 Energy=                 -611.125802
 G3 Enthalpy=              -611.125073 G3 Free Energy=            -611.181136

Temperature=               240.000000 Pressure=                   1.000000
 E(ZPE)=                      0.190407 E(Thermal)=                   0.207242
 E(QCISD(T))=              -609.809288 E(Empiric)=                  -0.204352
 DE(Plus)=                   -0.089735 DE(2DF)=                     -0.591426
 E(Delta-G3)=                -0.637178 E(G3-Empiric)=               -0.204352
 G3(0 K)=                  -611.141571 G3 Energy=                 -611.124737
 G3 Enthalpy=              -611.123977 G3 Free Energy=            -611.183597

Temperature=               250.000000 Pressure=                   1.000000
 E(ZPE)=                      0.190407 E(Thermal)=                   0.208324
 E(QCISD(T))=              -609.809288 E(Empiric)=                  -0.204352
 DE(Plus)=                   -0.089735 DE(2DF)=                     -0.591426
 E(Delta-G3)=                -0.637178 E(G3-Empiric)=               -0.204352
 G3(0 K)=                  -611.141571 G3 Energy=                 -611.123654
 G3 Enthalpy=              -611.122862 G3 Free Energy=            -611.186104

Temperature=               260.000000 Pressure=                   1.000000
 E(ZPE)=                      0.190407 E(Thermal)=                   0.209424
 E(QCISD(T))=              -609.809288 E(Empiric)=                  -0.204352
 DE(Plus)=                   -0.089735 DE(2DF)=                     -0.591426
 E(Delta-G3)=                -0.637178 E(G3-Empiric)=               -0.204352
 G3(0 K)=                  -611.141571 G3 Energy=                 -611.122554
 G3 Enthalpy=              -611.121731 G3 Free Energy=            -611.188656

Temperature=               273.150000 Pressure=                   1.000000
 E(ZPE)=                      0.190407 E(Thermal)=                   0.210895
 E(QCISD(T))=              -609.809288 E(Empiric)=                  -0.204352
 DE(Plus)=                   -0.089735 DE(2DF)=                     -0.591426
 E(Delta-G3)=                -0.637178 E(G3-Empiric)=               -0.204352
 G3(0 K)=                  -611.141571 G3 Energy=                 -611.121083
 G3 Enthalpy=              -611.120218 G3 Free Energy=            -611.192078

Temperature=               298.150000 Pressure=                   1.000000
```

```
 E(ZPE)=                          0.190407 E(Thermal)=                         0.213761
 E(QCISD(T))=                  -609.809288 E(Empiric)=                        -0.204352
 DE(Plus)=                       -0.089735 DE(2DF)=                           -0.591426
 E(Delta-G3)=                    -0.637178 E(G3-Empiric)=                     -0.204352
 G3(0 K)=                      -611.141571 G3 Energy=                       -611.118217
 G3 Enthalpy=                  -611.117273 G3 Free Energy=                  -611.198786

Temperature=                   310.000000 Pressure=                          1.000000
 E(ZPE)=                          0.190407 E(Thermal)=                         0.215150
 E(QCISD(T))=                  -609.809288 E(Empiric)=                        -0.204352
 DE(Plus)=                       -0.089735 DE(2DF)=                           -0.591426
 E(Delta-G3)=                    -0.637178 E(G3-Empiric)=                     -0.204352
 G3(0 K)=                      -611.141571 G3 Energy=                       -611.116828
 G3 Enthalpy=                  -611.115847 G3 Free Energy=                  -611.202053

Temperature=                   320.000000 Pressure=                          1.000000
 E(ZPE)=                          0.190407 E(Thermal)=                         0.216336
 E(QCISD(T))=                  -609.809288 E(Empiric)=                        -0.204352
 DE(Plus)=                       -0.089735 DE(2DF)=                           -0.591426
 E(Delta-G3)=                    -0.637178 E(G3-Empiric)=                     -0.204352
 G3(0 K)=                      -611.141571 G3 Energy=                       -611.115643
 G3 Enthalpy=                  -611.114629 G3 Free Energy=                  -611.204853
```

Molecular Formula Structure
(H2O)9 Nonamer

```
27
        Energy: -429381.6113075
O          1.57759       -2.02405        0.17943
H          0.97291       -2.01086       -0.59320
H          0.99187       -1.79121        0.93090
O          1.53471        2.00870       -0.21957
H          2.24044        1.30250       -0.14983
H          2.00529        2.82903       -0.43363
O         -0.40337       -1.58837       -1.79899
H         -0.43727       -1.99206       -2.67989
H         -0.36163       -0.59899       -1.95548
O         -0.32085       -1.08272        2.07366
H         -0.30655       -0.08143        2.02258
H         -0.35853       -1.29682        3.01852
O          3.31473        0.01565       -0.08277
H          2.72588       -0.79005       -0.02645
H          3.87669       -0.03780        0.70494
O         -2.46980        1.29219       -0.11852
H         -2.54296        0.29757       -0.00692
H         -3.37985        1.62427       -0.15754
O         -0.42545        1.07992       -2.04922
H         -1.22510        1.30982       -1.52912
H          0.31317        1.44897       -1.51734
O         -0.41243        1.58061        1.78424
H         -1.21717        1.66632        1.22845
H          0.31704        1.84720        1.18376
O         -2.39454       -1.36419        0.18070
H         -1.83295       -1.63151       -0.57962
H         -1.78598       -1.43690        0.94821
```

G3 Energies at Each Temperature for (H2O)9 Nonamer

```
Temperature=              200.000000 Pressure=                      1.000000
 E(ZPE)=                     0.218878 E(Thermal)=                    0.231282
 E(QCISD(T))=             -686.048600 E(Empiric)=                   -0.229896
 DE(Plus)=                  -0.100673 DE(2DF)=                      -0.664559
 E(Delta-G3)=               -0.716494 E(G3-Empiric)=                -0.229896
 G3(0 K)=                 -687.541344 G3 Energy=                  -687.528940
 G3 Enthalpy=             -687.528307 G3 Free Energy=             -687.570175

Temperature=              210.000000 Pressure=                      1.000000
 E(ZPE)=                     0.218878 E(Thermal)=                    0.232340
 E(QCISD(T))=             -686.048600 E(Empiric)=                   -0.229896
 DE(Plus)=                  -0.100673 DE(2DF)=                      -0.664559
 E(Delta-G3)=               -0.716494 E(G3-Empiric)=                -0.229896
 G3(0 K)=                 -687.541344 G3 Energy=                  -687.527882
 G3 Enthalpy=             -687.527217 G3 Free Energy=             -687.572295

Temperature=              216.650000 Pressure=                      1.000000
 E(ZPE)=                     0.218878 E(Thermal)=                    0.233059
 E(QCISD(T))=             -686.048600 E(Empiric)=                   -0.229896
 DE(Plus)=                  -0.100673 DE(2DF)=                      -0.664559
 E(Delta-G3)=               -0.716494 E(G3-Empiric)=                -0.229896
 G3(0 K)=                 -687.541344 G3 Energy=                  -687.527163
 G3 Enthalpy=             -687.526477 G3 Free Energy=             -687.573734

Temperature=              230.000000 Pressure=                      1.000000
 E(ZPE)=                     0.218878 E(Thermal)=                    0.234536
 E(QCISD(T))=             -686.048600 E(Empiric)=                   -0.229896
 DE(Plus)=                  -0.100673 DE(2DF)=                      -0.664559
 E(Delta-G3)=               -0.716494 E(G3-Empiric)=                -0.229896
 G3(0 K)=                 -687.541344 G3 Energy=                  -687.525686
 G3 Enthalpy=             -687.524958 G3 Free Energy=             -687.576691

Temperature=              240.000000 Pressure=                      1.000000
 E(ZPE)=                     0.218878 E(Thermal)=                    0.235671
 E(QCISD(T))=             -686.048600 E(Empiric)=                   -0.229896
 DE(Plus)=                  -0.100673 DE(2DF)=                      -0.664559
 E(Delta-G3)=               -0.716494 E(G3-Empiric)=                -0.229896
 G3(0 K)=                 -687.541344 G3 Energy=                  -687.524551
 G3 Enthalpy=             -687.523791 G3 Free Energy=             -687.578966

Temperature=              250.000000 Pressure=                      1.000000
 E(ZPE)=                     0.218878 E(Thermal)=                    0.236829
 E(QCISD(T))=             -686.048600 E(Empiric)=                   -0.229896
 DE(Plus)=                  -0.100673 DE(2DF)=                      -0.664559
 E(Delta-G3)=               -0.716494 E(G3-Empiric)=                -0.229896
 G3(0 K)=                 -687.541344 G3 Energy=                  -687.523393
 G3 Enthalpy=             -687.522602 G3 Free Energy=             -687.581289

Temperature=              260.000000 Pressure=                      1.000000
 E(ZPE)=                     0.218878 E(Thermal)=                    0.238008
 E(QCISD(T))=             -686.048600 E(Empiric)=                   -0.229896
 DE(Plus)=                  -0.100673 DE(2DF)=                      -0.664559
 E(Delta-G3)=               -0.716494 E(G3-Empiric)=                -0.229896
```

```
 G3(0 K)=                    -687.541344 G3 Energy=                   -687.522214
 G3 Enthalpy=                -687.521390 G3 Free Energy=              -687.583660

Temperature=                 273.150000 Pressure=                       1.000000
 E(ZPE)=                        0.218878 E(Thermal)=                    0.239591
 E(QCISD(T))=                -686.048600 E(Empiric)=                   -0.229896
 DE(Plus)=                     -0.100673 DE(2DF)=                      -0.664559
 E(Delta-G3)=                  -0.716494 E(G3-Empiric)=                -0.229896
 G3(0 K)=                    -687.541344 G3 Energy=                   -687.520631
 G3 Enthalpy=                -687.519766 G3 Free Energy=              -687.586850

Temperature=                 298.150000 Pressure=                       1.000000
 E(ZPE)=                        0.218878 E(Thermal)=                    0.242690
 E(QCISD(T))=                -686.048600 E(Empiric)=                   -0.229896
 DE(Plus)=                     -0.100673 DE(2DF)=                      -0.664559
 E(Delta-G3)=                  -0.716494 E(G3-Empiric)=                -0.229896
 G3(0 K)=                    -687.541344 G3 Energy=                   -687.517532
 G3 Enthalpy=                -687.516588 G3 Free Energy=              -687.593130

Temperature=                 310.000000 Pressure=                       1.000000
 E(ZPE)=                        0.218878 E(Thermal)=                    0.244197
 E(QCISD(T))=                -686.048600 E(Empiric)=                   -0.229896
 DE(Plus)=                     -0.100673 DE(2DF)=                      -0.664559
 E(Delta-G3)=                  -0.716494 E(G3-Empiric)=                -0.229896
 G3(0 K)=                    -687.541344 G3 Energy=                   -687.516025
 G3 Enthalpy=                -687.515043 G3 Free Energy=              -687.596202

Temperature=                 320.000000 Pressure=                       1.000000
 E(ZPE)=                        0.218878 E(Thermal)=                    0.245487
 E(QCISD(T))=                -686.048600 E(Empiric)=                   -0.229896
 DE(Plus)=                     -0.100673 DE(2DF)=                      -0.664559
 E(Delta-G3)=                  -0.716494 E(G3-Empiric)=                -0.229896
 G3(0 K)=                    -687.541344 G3 Energy=                   -687.514735
 G3 Enthalpy=                -687.513721 G3 Free Energy=              -687.598842
```

Molecular Formula Structure
(H2O)10 Decamer-172

```
30
G3_172.log      Energy: -477316.6536821
O          -1.88465        1.36261        1.42070
H          -0.99817        1.77974        1.46660
H          -2.11981        1.41815        0.46788
O           2.19006       -0.04185       -1.61747
H           2.50697        0.08823       -0.70108
H           1.61600        0.75040       -1.77923
O           0.50615        2.13889       -1.68302
H           0.60698        2.42617       -0.75428
H          -0.41792        1.81350       -1.72256
O           0.73290       -2.23722       -1.34749
H           1.26756       -1.40173       -1.52764
H           1.09214       -2.91005       -1.94630
O          -1.98445       -1.56456       -1.10991
H          -1.07584       -1.86850       -1.33054
H          -2.02230       -1.64188       -0.13265
O          -1.67800       -1.28546        1.69615
```

H	-2.29102	-1.57132	2.39088
H	-1.74760	-0.28441	1.66835
O	0.76202	2.36652	1.19019
H	1.40858	1.62406	1.29971
H	1.12885	3.11775	1.68079
O	1.01742	-2.03135	1.43672
H	0.08414	-1.82934	1.67089
H	0.96499	-2.31556	0.49695
O	2.43311	0.19803	1.23858
H	3.22917	0.08125	1.78007
H	1.89157	-0.64242	1.37309
O	-2.11736	1.09022	-1.36373
H	-2.86895	1.31184	-1.93480
H	-2.07300	0.08757	-1.35188

G3 Energies at Each Temperature for (H2O)10 Decamer-172

```
Temperature=                200.000000 Pressure=                       1.000000
 E(ZPE)=                       0.244007 E(Thermal)=                      0.257616
 E(QCISD(T))=               -762.281183 E(Empiric)=                     -0.255440
 DE(Plus)=                    -0.112169 DE(2DF)=                        -0.738316
 E(Delta-G3)=                 -0.795304 E(G3-Empiric)=                  -0.255440
 G3(0 K)=                   -763.938405 G3 Energy=                    -763.924796
 G3 Enthalpy=               -763.924163 G3 Free Energy=               -763.968381

Temperature=                210.000000 Pressure=                       1.000000
 E(ZPE)=                       0.244007 E(Thermal)=                      0.258788
 E(QCISD(T))=               -762.281183 E(Empiric)=                     -0.255440
 DE(Plus)=                    -0.112169 DE(2DF)=                        -0.738316
 E(Delta-G3)=                 -0.795304 E(G3-Empiric)=                  -0.255440
 G3(0 K)=                   -763.938405 G3 Energy=                    -763.923625
 G3 Enthalpy=               -763.922960 G3 Free Energy=               -763.970621

Temperature=                216.650000 Pressure=                       1.000000
 E(ZPE)=                       0.244007 E(Thermal)=                      0.259584
 E(QCISD(T))=               -762.281183 E(Empiric)=                     -0.255440
 DE(Plus)=                    -0.112169 DE(2DF)=                        -0.738316
 E(Delta-G3)=                 -0.795304 E(G3-Empiric)=                  -0.255440
 G3(0 K)=                   -763.938405 G3 Energy=                    -763.922828
 G3 Enthalpy=               -763.922142 G3 Free Energy=               -763.972143

Temperature=                230.000000 Pressure=                       1.000000
 E(ZPE)=                       0.244007 E(Thermal)=                      0.261221
 E(QCISD(T))=               -762.281183 E(Empiric)=                     -0.255440
 DE(Plus)=                    -0.112169 DE(2DF)=                        -0.738316
 E(Delta-G3)=                 -0.795304 E(G3-Empiric)=                  -0.255440
 G3(0 K)=                   -763.938405 G3 Energy=                    -763.921191
 G3 Enthalpy=               -763.920463 G3 Free Energy=               -763.975275

Temperature=                240.000000 Pressure=                       1.000000
 E(ZPE)=                       0.244007 E(Thermal)=                      0.262479
 E(QCISD(T))=               -762.281183 E(Empiric)=                     -0.255440
 DE(Plus)=                    -0.112169 DE(2DF)=                        -0.738316
 E(Delta-G3)=                 -0.795304 E(G3-Empiric)=                  -0.255440
 G3(0 K)=                   -763.938405 G3 Energy=                    -763.919933
```

```
 G3 Enthalpy=              -763.919173 G3 Free Energy=             -763.977686

Temperature=               250.000000 Pressure=                       1.000000
 E(ZPE)=                      0.244007 E(Thermal)=                     0.263764
 E(QCISD(T))=              -762.281183 E(Empiric)=                    -0.255440
 DE(Plus)=                   -0.112169 DE(2DF)=                       -0.738316
 E(Delta-G3)=                -0.795304 E(G3-Empiric)=                 -0.255440
 G3(0 K)=                  -763.938405 G3 Energy=                  -763.918649
 G3 Enthalpy=              -763.917857 G3 Free Energy=             -763.980151

Temperature=               260.000000 Pressure=                       1.000000
 E(ZPE)=                      0.244007 E(Thermal)=                     0.265073
 E(QCISD(T))=              -762.281183 E(Empiric)=                    -0.255440
 DE(Plus)=                   -0.112169 DE(2DF)=                       -0.738316
 E(Delta-G3)=                -0.795304 E(G3-Empiric)=                 -0.255440
 G3(0 K)=                  -763.938405 G3 Energy=                  -763.917340
 G3 Enthalpy=              -763.916516 G3 Free Energy=             -763.982669

Temperature=               273.150000 Pressure=                       1.000000
 E(ZPE)=                      0.244007 E(Thermal)=                     0.266829
 E(QCISD(T))=              -762.281183 E(Empiric)=                    -0.255440
 DE(Plus)=                   -0.112169 DE(2DF)=                       -0.738316
 E(Delta-G3)=                -0.795304 E(G3-Empiric)=                 -0.255440
 G3(0 K)=                  -763.938405 G3 Energy=                  -763.915583
 G3 Enthalpy=              -763.914718 G3 Free Energy=             -763.986059

Temperature=               298.150000 Pressure=                       1.000000
 E(ZPE)=                      0.244007 E(Thermal)=                     0.270272
 E(QCISD(T))=              -762.281183 E(Empiric)=                    -0.255440
 DE(Plus)=                   -0.112169 DE(2DF)=                       -0.738316
 E(Delta-G3)=                -0.795304 E(G3-Empiric)=                 -0.255440
 G3(0 K)=                  -763.938405 G3 Energy=                  -763.912140
 G3 Enthalpy=              -763.911196 G3 Free Energy=             -763.992744

Temperature=               310.000000 Pressure=                       1.000000
 E(ZPE)=                      0.244007 E(Thermal)=                     0.271948
 E(QCISD(T))=              -762.281183 E(Empiric)=                    -0.255440
 DE(Plus)=                   -0.112169 DE(2DF)=                       -0.738316
 E(Delta-G3)=                -0.795304 E(G3-Empiric)=                 -0.255440
 G3(0 K)=                  -763.938405 G3 Energy=                  -763.910465
 G3 Enthalpy=              -763.909483 G3 Free Energy=             -763.996019

Temperature=               320.000000 Pressure=                       1.000000
 E(ZPE)=                      0.244007 E(Thermal)=                     0.273382
 E(QCISD(T))=              -762.281183 E(Empiric)=                    -0.255440
 DE(Plus)=                   -0.112169 DE(2DF)=                       -0.738316
 E(Delta-G3)=                -0.795304 E(G3-Empiric)=                 -0.255440
 G3(0 K)=                  -763.938405 G3 Energy=                  -763.909030
 G3 Enthalpy=              -763.908017 G3 Free Energy=             -763.998834
```

Molecular Formula Structure
(H2O)10 Decamer-272

```
30
        Energy: -477055.7619373
O           0.22765       -1.49807       -0.40453
```

H	0.29310	-0.52159	-0.23119
H	-0.15284	-1.85075	0.44242
O	-2.44274	-1.70276	-1.06843
H	-1.45577	-1.63486	-1.06397
H	-2.66876	-2.29321	-1.80300
O	-1.46425	-2.15938	1.64921
H	-2.14369	-2.27686	0.95933
H	-1.63995	-1.25365	1.98392
O	2.65381	2.14919	0.80561
H	3.27479	1.42806	0.52626
H	2.91834	2.91626	0.27574
O	-3.36121	0.92804	-0.44709
H	-3.07114	0.90303	0.48783
H	-3.18546	0.01628	-0.76065
O	4.32201	0.15304	-0.04963
H	4.89791	-0.16937	0.66004
H	3.80941	-0.64760	-0.32805
O	0.18973	1.15232	0.25046
H	1.06015	1.57982	0.47067
H	-0.25237	1.70465	-0.44631
O	-1.39276	2.49341	-1.49929
H	-2.20325	2.01061	-1.18313
H	-1.33710	2.29128	-2.44524
O	-1.83634	0.63124	1.96285
H	-1.84666	1.18340	2.75988
H	-1.02804	0.90322	1.44982
O	2.90598	-2.10270	-0.71890
H	2.99446	-2.36682	-1.64672
H	1.93499	-1.96623	-0.58793

G3 Energies at Each Temperature for (H2O)10 Decamer-272

```
Temperature=              200.000000 Pressure=                  1.000000
 E(ZPE)=                     0.240063 E(Thermal)=                 0.255624
 E(QCISD(T))=             -762.266789 E(Empiric)=                -0.255440
 DE(Plus)=                  -0.111677 DE(2DF)=                   -0.737915
 E(Delta-G3)=               -0.797063 E(G3-Empiric)=             -0.255440
 G3(0 K)=                 -763.928821 G3 Energy=               -763.913261
 G3 Enthalpy=             -763.912627 G3 Free Energy=          -763.964764

Temperature=              210.000000 Pressure=                  1.000000
 E(ZPE)=                     0.240063 E(Thermal)=                 0.256867
 E(QCISD(T))=             -762.266789 E(Empiric)=                -0.255440
 DE(Plus)=                  -0.111677 DE(2DF)=                   -0.737915
 E(Delta-G3)=               -0.797063 E(G3-Empiric)=             -0.255440
 G3(0 K)=                 -763.928821 G3 Energy=               -763.912018
 G3 Enthalpy=             -763.911352 G3 Free Energy=          -763.967402

Temperature=              216.650000 Pressure=                  1.000000
 E(ZPE)=                     0.240063 E(Thermal)=                 0.257709
 E(QCISD(T))=             -762.266789 E(Empiric)=                -0.255440
 DE(Plus)=                  -0.111677 DE(2DF)=                   -0.737915
 E(Delta-G3)=               -0.797063 E(G3-Empiric)=             -0.255440
 G3(0 K)=                 -763.928821 G3 Energy=               -763.911176
 G3 Enthalpy=             -763.910490 G3 Free Energy=          -763.969190
```

```
Temperature=              230.000000 Pressure=                      1.000000
 E(ZPE)=                     0.240063 E(Thermal)=                    0.259433
 E(QCISD(T))=             -762.266789 E(Empiric)=                   -0.255440
 DE(Plus)=                  -0.111677 DE(2DF)=                      -0.737915
 E(Delta-G3)=               -0.797063 E(G3-Empiric)=                -0.255440
 G3(0 K)=                 -763.928821 G3 Energy=                  -763.909451
 G3 Enthalpy=             -763.908723 G3 Free Energy=             -763.972861

Temperature=              240.000000 Pressure=                      1.000000
 E(ZPE)=                     0.240063 E(Thermal)=                    0.260753
 E(QCISD(T))=             -762.266789 E(Empiric)=                   -0.255440
 DE(Plus)=                  -0.111677 DE(2DF)=                      -0.737915
 E(Delta-G3)=               -0.797063 E(G3-Empiric)=                -0.255440
 G3(0 K)=                 -763.928821 G3 Energy=                  -763.908131
 G3 Enthalpy=             -763.907371 G3 Free Energy=             -763.975678

Temperature=              250.000000 Pressure=                      1.000000
 E(ZPE)=                     0.240063 E(Thermal)=                    0.262096
 E(QCISD(T))=             -762.266789 E(Empiric)=                   -0.255440
 DE(Plus)=                  -0.111677 DE(2DF)=                      -0.737915
 E(Delta-G3)=               -0.797063 E(G3-Empiric)=                -0.255440
 G3(0 K)=                 -763.928821 G3 Energy=                  -763.906788
 G3 Enthalpy=             -763.905997 G3 Free Energy=             -763.978552

Temperature=              260.000000 Pressure=                      1.000000
 E(ZPE)=                     0.240063 E(Thermal)=                    0.263461
 E(QCISD(T))=             -762.266789 E(Empiric)=                   -0.255440
 DE(Plus)=                  -0.111677 DE(2DF)=                      -0.737915
 E(Delta-G3)=               -0.797063 E(G3-Empiric)=                -0.255440
 G3(0 K)=                 -763.928821 G3 Energy=                  -763.905423
 G3 Enthalpy=             -763.904600 G3 Free Energy=             -763.981482

Temperature=              273.150000 Pressure=                      1.000000
 E(ZPE)=                     0.240063 E(Thermal)=                    0.265288
 E(QCISD(T))=             -762.266789 E(Empiric)=                   -0.255440
 DE(Plus)=                  -0.111677 DE(2DF)=                      -0.737915
 E(Delta-G3)=               -0.797063 E(G3-Empiric)=                -0.255440
 G3(0 K)=                 -763.928821 G3 Energy=                  -763.903597
 G3 Enthalpy=             -763.902732 G3 Free Energy=             -763.985417

Temperature=              298.150000 Pressure=                      1.000000
 E(ZPE)=                     0.240063 E(Thermal)=                    0.268852
 E(QCISD(T))=             -762.266789 E(Empiric)=                   -0.255440
 DE(Plus)=                  -0.111677 DE(2DF)=                      -0.737915
 E(Delta-G3)=               -0.797063 E(G3-Empiric)=                -0.255440
 G3(0 K)=                 -763.928821 G3 Energy=                  -763.900032
 G3 Enthalpy=             -763.899088 G3 Free Energy=             -763.993145

Temperature=              310.000000 Pressure=                      1.000000
 E(ZPE)=                     0.240063 E(Thermal)=                    0.270580
 E(QCISD(T))=             -762.266789 E(Empiric)=                   -0.255440
 DE(Plus)=                  -0.111677 DE(2DF)=                      -0.737915
 E(Delta-G3)=               -0.797063 E(G3-Empiric)=                -0.255440
 G3(0 K)=                 -763.928821 G3 Energy=                  -763.898304
 G3 Enthalpy=             -763.897322 G3 Free Energy=             -763.996918
```

Temperature=	320.000000	Pressure=	1.000000
E(ZPE)=	0.240063	E(Thermal)=	0.272057
E(QCISD(T))=	-762.266789	E(Empiric)=	-0.255440
DE(Plus)=	-0.111677	DE(2DF)=	-0.737915
E(Delta-G3)=	-0.797063	E(G3-Empiric)=	-0.255440
G3(0 K)=	-763.928821	G3 Energy=	-763.896827
G3 Enthalpy=	-763.895814	G3 Free Energy=	-764.000155

Molecular Formula   Structure
(H2O)11   Undecamer
33
Energy: -524800.3695788

O	-0.00614	-2.65865	-0.51479
H	0.79189	-2.52596	0.04443
H	-0.76960	-2.54390	0.09465
O	2.74129	-0.24979	-1.33900
H	2.73199	0.64586	-0.93150
H	1.87627	-0.31286	-1.79022
O	2.18565	2.05099	0.11929
H	1.92283	1.55126	0.95969
H	2.76510	2.77324	0.40671
O	-2.24874	2.07128	0.18941
H	-2.74396	2.90490	0.19299
H	-1.44062	2.23310	-0.37876
O	1.52688	0.59707	2.20733
H	1.78343	-0.30757	1.93458
H	0.54330	0.56491	2.32952
O	-2.84932	-0.30160	-1.14712
H	-2.83587	0.59359	-0.73897
H	-2.01334	-0.34308	-1.65249
O	-2.21263	-1.82614	0.97137
H	-2.55907	-1.30576	0.19079
H	-2.95071	-2.37911	1.27146
O	-1.22333	0.49797	2.35989
H	-1.56807	-0.36126	2.04324
H	-1.61424	1.14932	1.74306
O	-0.07847	-0.61062	-2.18229
H	-0.05223	-1.40660	-1.55432
H	-0.10663	-0.99347	-3.07499
O	2.28792	-1.79202	0.82013
H	3.04669	-2.35440	1.04090
H	2.56414	-1.26266	0.01800
O	-0.12237	2.22715	-1.41801
H	0.70437	2.31113	-0.89005
H	-0.08154	1.32430	-1.78843

G3 Energies at Each Temperature for (H2O)11 Undecamer

Temperature=	200.000000	Pressure=	1.000000
E(ZPE)=	0.267824	E(Thermal)=	0.283217
E(QCISD(T))=	-838.510169	E(Empiric)=	-0.280984
DE(Plus)=	-0.121390	DE(2DF)=	-0.812214
E(Delta-G3)=	-0.875404	E(G3-Empiric)=	-0.280984
G3(0 K)=	-840.332338	G3 Energy=	-840.316944

```
 G3 Enthalpy=                -840.316311 G3 Free Energy=            -840.365188

Temperature=                 210.000000 Pressure=                     1.000000
 E(ZPE)=                        0.267824 E(Thermal)=                  0.284526
 E(QCISD(T))=                -838.510169 E(Empiric)=                 -0.280984
 DE(Plus)=                     -0.121390 DE(2DF)=                    -0.812214
 E(Delta-G3)=                  -0.875404 E(G3-Empiric)=              -0.280984
 G3(0 K)=                    -840.332338 G3 Energy=                -840.315636
 G3 Enthalpy=                -840.314971 G3 Free Energy=            -840.367665

Temperature=                 216.650000 Pressure=                     1.000000
 E(ZPE)=                        0.267824 E(Thermal)=                  0.285414
 E(QCISD(T))=                -838.510169 E(Empiric)=                 -0.280984
 DE(Plus)=                     -0.121390 DE(2DF)=                    -0.812214
 E(Delta-G3)=                  -0.875404 E(G3-Empiric)=              -0.280984
 G3(0 K)=                    -840.332338 G3 Energy=                -840.314748
 G3 Enthalpy=                -840.314062 G3 Free Energy=            -840.369348

Temperature=                 230.000000 Pressure=                     1.000000
 E(ZPE)=                        0.267824 E(Thermal)=                  0.287239
 E(QCISD(T))=                -838.510169 E(Empiric)=                 -0.280984
 DE(Plus)=                     -0.121390 DE(2DF)=                    -0.812214
 E(Delta-G3)=                  -0.875404 E(G3-Empiric)=              -0.280984
 G3(0 K)=                    -840.332338 G3 Energy=                -840.312922
 G3 Enthalpy=                -840.312194 G3 Free Energy=            -840.372810

Temperature=                 240.000000 Pressure=                     1.000000
 E(ZPE)=                        0.267824 E(Thermal)=                  0.288642
 E(QCISD(T))=                -838.510169 E(Empiric)=                 -0.280984
 DE(Plus)=                     -0.121390 DE(2DF)=                    -0.812214
 E(Delta-G3)=                  -0.875404 E(G3-Empiric)=              -0.280984
 G3(0 K)=                    -840.332338 G3 Energy=                -840.311520
 G3 Enthalpy=                -840.310760 G3 Free Energy=            -840.375477

Temperature=                 250.000000 Pressure=                     1.000000
 E(ZPE)=                        0.267824 E(Thermal)=                  0.290072
 E(QCISD(T))=                -838.510169 E(Empiric)=                 -0.280984
 DE(Plus)=                     -0.121390 DE(2DF)=                    -0.812214
 E(Delta-G3)=                  -0.875404 E(G3-Empiric)=              -0.280984
 G3(0 K)=                    -840.332338 G3 Energy=                -840.310089
 G3 Enthalpy=                -840.309298 G3 Free Energy=            -840.378203

Temperature=                 260.000000 Pressure=                     1.000000
 E(ZPE)=                        0.267824 E(Thermal)=                  0.291529
 E(QCISD(T))=                -838.510169 E(Empiric)=                 -0.280984
 DE(Plus)=                     -0.121390 DE(2DF)=                    -0.812214
 E(Delta-G3)=                  -0.875404 E(G3-Empiric)=              -0.280984
 G3(0 K)=                    -840.332338 G3 Energy=                -840.308632
 G3 Enthalpy=                -840.307809 G3 Free Energy=            -840.380989

Temperature=                 273.150000 Pressure=                     1.000000
 E(ZPE)=                        0.267824 E(Thermal)=                  0.293484
 E(QCISD(T))=                -838.510169 E(Empiric)=                 -0.280984
 DE(Plus)=                     -0.121390 DE(2DF)=                    -0.812214
 E(Delta-G3)=                  -0.875404 E(G3-Empiric)=              -0.280984
 G3(0 K)=                    -840.332338 G3 Energy=                -840.306677
```

```
 G3 Enthalpy=              -840.305812 G3 Free Energy=              -840.384739

Temperature=               298.150000 Pressure=                       1.000000
 E(ZPE)=                      0.267824 E(Thermal)=                     0.297313
 E(QCISD(T))=              -838.510169 E(Empiric)=                    -0.280984
 DE(Plus)=                   -0.121390 DE(2DF)=                       -0.812214
 E(Delta-G3)=                -0.875404 E(G3-Empiric)=                 -0.280984
 G3(0 K)=                  -840.332338 G3 Energy=                   -840.302848
 G3 Enthalpy=              -840.301904 G3 Free Energy=              -840.392136

Temperature=               310.000000 Pressure=                       1.000000
 E(ZPE)=                      0.267824 E(Thermal)=                     0.299176
 E(QCISD(T))=              -838.510169 E(Empiric)=                    -0.280984
 DE(Plus)=                   -0.121390 DE(2DF)=                       -0.812214
 E(Delta-G3)=                -0.875404 E(G3-Empiric)=                 -0.280984
 G3(0 K)=                  -840.332338 G3 Energy=                   -840.300986
 G3 Enthalpy=              -840.300004 G3 Free Energy=              -840.395759

Temperature=               320.000000 Pressure=                       1.000000
 E(ZPE)=                      0.267824 E(Thermal)=                     0.300769
 E(QCISD(T))=              -838.510169 E(Empiric)=                    -0.280984
 DE(Plus)=                   -0.121390 DE(2DF)=                       -0.812214
 E(Delta-G3)=                -0.875404 E(G3-Empiric)=                 -0.280984
 G3(0 K)=                  -840.332338 G3 Energy=                   -840.299392
 G3 Enthalpy=              -840.298379 G3 Free Energy=              -840.398874
```

Molecular Formula (H2O)12

Structure Dodecamer-73

```
36
        Energy: -572598.9041391
O         -0.01154        1.30764       -1.43464
H         -0.02418        0.31722       -1.49310
H         -0.92944        1.57099       -1.66642
O          0.00777       -1.43475       -1.31163
H          0.92980       -1.65517       -1.57119
H          0.01671       -1.49078       -0.32118
O         -2.79599        1.43681        1.29208
H         -2.89945        0.48374        1.50407
H         -1.87234        1.63917        1.54991
O          0.00777        1.43475        1.31162
H          0.92980        1.65517        1.57119
H          0.01671        1.49078        0.32117
O         -2.78331        1.40647       -1.39660
H         -2.83479        1.49025       -0.39906
H         -3.49121        1.97027       -1.74525
O         -0.01154       -1.30764        1.43463
H         -0.02418       -0.31722        1.49309
H         -0.92944       -1.57099        1.66642
O          2.79630        1.41095       -1.31671
H          1.86859        1.60165       -1.56957
H          2.90697        0.45691       -1.52068
O          2.78680        1.43103        1.36862
H          2.84823        1.48846        0.36933
H          3.48089        2.01716        1.70833
O         -2.78331       -1.40647        1.39661
```

H	-2.83479	-1.49026	0.39907
H	-3.49121	-1.97026	1.74527
O	2.79630	-1.41095	1.31671
H	1.86859	-1.60165	1.56957
H	2.90697	-0.45691	1.52068
O	2.78680	-1.43103	-1.36862
H	3.48089	-2.01715	-1.70833
H	2.84822	-1.48847	-0.36933
O	-2.79600	-1.43680	-1.29208
H	-1.87235	-1.63917	-1.54992
H	-2.89946	-0.48373	-1.50406

G3 Energies at Each Temperature for (H2O)12 Dodecamer-73

```
Temperature=              200.000000 Pressure=                     1.000000
 E(ZPE)=                     0.295279 E(Thermal)=                   0.311062
 E(QCISD(T))=             -914.754612 E(Empiric)=                  -0.306528
 DE(Plus)=                  -0.127080 DE(2DF)=                     -0.885593
 E(Delta-G3)=               -0.953864 E(G3-Empiric)=               -0.306528
 G3(0 K)=                 -916.732398 G3 Energy=                 -916.716615
 G3 Enthalpy=             -916.715981 G3 Free Energy=            -916.764618

Temperature=              210.000000 Pressure=                     1.000000
 E(ZPE)=                     0.295279 E(Thermal)=                   0.312445
 E(QCISD(T))=             -914.754612 E(Empiric)=                  -0.306528
 DE(Plus)=                  -0.127080 DE(2DF)=                     -0.885593
 E(Delta-G3)=               -0.953864 E(G3-Empiric)=               -0.306528
 G3(0 K)=                 -916.732398 G3 Energy=                 -916.715233
 G3 Enthalpy=             -916.714568 G3 Free Energy=            -916.767085

Temperature=              216.650000 Pressure=                     1.000000
 E(ZPE)=                     0.295279 E(Thermal)=                   0.313385
 E(QCISD(T))=             -914.754612 E(Empiric)=                  -0.306528
 DE(Plus)=                  -0.127080 DE(2DF)=                     -0.885593
 E(Delta-G3)=               -0.953864 E(G3-Empiric)=               -0.306528
 G3(0 K)=                 -916.732398 G3 Energy=                 -916.714292
 G3 Enthalpy=             -916.713606 G3 Free Energy=            -916.768763

Temperature=              230.000000 Pressure=                     1.000000
 E(ZPE)=                     0.295279 E(Thermal)=                   0.315321
 E(QCISD(T))=             -914.754612 E(Empiric)=                  -0.306528
 DE(Plus)=                  -0.127080 DE(2DF)=                     -0.885593
 E(Delta-G3)=               -0.953864 E(G3-Empiric)=               -0.306528
 G3(0 K)=                 -916.732398 G3 Energy=                 -916.712356
 G3 Enthalpy=             -916.711628 G3 Free Energy=            -916.772221

Temperature=              240.000000 Pressure=                     1.000000
 E(ZPE)=                     0.295279 E(Thermal)=                   0.316811
 E(QCISD(T))=             -914.754612 E(Empiric)=                  -0.306528
 DE(Plus)=                  -0.127080 DE(2DF)=                     -0.885593
 E(Delta-G3)=               -0.953864 E(G3-Empiric)=               -0.306528
 G3(0 K)=                 -916.732398 G3 Energy=                 -916.710866
 G3 Enthalpy=             -916.710106 G3 Free Energy=            -916.774888

Temperature=              250.000000 Pressure=                     1.000000
```

```
 E(ZPE)=                          0.295279 E(Thermal)=                    0.318334
 E(QCISD(T))=                  -914.754612 E(Empiric)=                   -0.306528
 DE(Plus)=                       -0.127080 DE(2DF)=                      -0.885593
 E(Delta-G3)=                    -0.953864 E(G3-Empiric)=                -0.306528
 G3(0 K)=                      -916.732398 G3 Energy=                  -916.709343
 G3 Enthalpy=                  -916.708552 G3 Free Energy=             -916.777619

Temperature=                   260.000000 Pressure=                      1.000000
 E(ZPE)=                          0.295279 E(Thermal)=                    0.319888
 E(QCISD(T))=                  -914.754612 E(Empiric)=                   -0.306528
 DE(Plus)=                       -0.127080 DE(2DF)=                      -0.885593
 E(Delta-G3)=                    -0.953864 E(G3-Empiric)=                -0.306528
 G3(0 K)=                      -916.732398 G3 Energy=                  -916.707790
 G3 Enthalpy=                  -916.706966 G3 Free Energy=             -916.780413

Temperature=                   273.150000 Pressure=                      1.000000
 E(ZPE)=                          0.295279 E(Thermal)=                    0.321975
 E(QCISD(T))=                  -914.754612 E(Empiric)=                   -0.306528
 DE(Plus)=                       -0.127080 DE(2DF)=                      -0.885593
 E(Delta-G3)=                    -0.953864 E(G3-Empiric)=                -0.306528
 G3(0 K)=                      -916.732398 G3 Energy=                  -916.705702
 G3 Enthalpy=                  -916.704837 G3 Free Energy=             -916.784180

Temperature=                   298.150000 Pressure=                      1.000000
 E(ZPE)=                          0.295279 E(Thermal)=                    0.326075
 E(QCISD(T))=                  -914.754612 E(Empiric)=                   -0.306528
 DE(Plus)=                       -0.127080 DE(2DF)=                      -0.885593
 E(Delta-G3)=                    -0.953864 E(G3-Empiric)=                -0.306528
 G3(0 K)=                      -916.732398 G3 Energy=                  -916.701602
 G3 Enthalpy=                  -916.700658 G3 Free Energy=             -916.791627

Temperature=                   310.000000 Pressure=                      1.000000
 E(ZPE)=                          0.295279 E(Thermal)=                    0.328073
 E(QCISD(T))=                  -914.754612 E(Empiric)=                   -0.306528
 DE(Plus)=                       -0.127080 DE(2DF)=                      -0.885593
 E(Delta-G3)=                    -0.953864 E(G3-Empiric)=                -0.306528
 G3(0 K)=                      -916.732398 G3 Energy=                  -916.699604
 G3 Enthalpy=                  -916.698623 G3 Free Energy=             -916.795282

Temperature=                   320.000000 Pressure=                      1.000000
 E(ZPE)=                          0.295279 E(Thermal)=                    0.329785
 E(QCISD(T))=                  -914.754612 E(Empiric)=                   -0.306528
 DE(Plus)=                       -0.127080 DE(2DF)=                      -0.885593
 E(Delta-G3)=                    -0.953864 E(G3-Empiric)=                -0.306528
 G3(0 K)=                      -916.732398 G3 Energy=                  -916.697893
 G3 Enthalpy=                  -916.696879 G3 Free Energy=             -916.798428
```

Molecular Formula Structure
(H2O)12 Dodecamer-168

```
36
        Energy: -572480.4276361
O          -1.46133          0.08935        -1.36778
H          -2.43355          0.21768        -1.51466
H          -1.03381          0.97476        -1.43313
O           2.68403         -1.05575        -1.65258
```

H	3.21190	-1.47985	-2.34666
H	1.75975	-1.43963	-1.74314
O	-0.00772	2.42999	-1.08467
H	0.95814	2.17280	-1.19571
H	-0.11281	3.26351	-1.56915
O	2.62182	1.26808	1.60229
H	1.65949	1.52960	1.72637
H	3.11482	1.75517	2.28046
O	2.79013	-1.50152	1.13332
H	2.95809	-1.43134	0.16845
H	2.79399	-0.56668	1.43684
O	2.55701	1.71813	-1.18396
H	2.64778	0.78819	-1.48921
H	2.77474	1.66935	-0.22780
O	0.06119	1.99389	1.72752
H	-0.49935	1.18411	1.75878
H	-0.07635	2.33428	0.81779
O	-4.20528	-0.37539	1.33275
H	-3.23113	-0.46443	1.47671
H	-4.47361	0.33815	1.93156
O	-4.18178	0.34831	-1.33329
H	-4.29343	0.12440	-0.37519
H	-4.66357	-0.34913	-1.80337
O	0.31362	-2.48250	1.13663
H	1.24869	-2.12089	1.21090
H	0.32139	-3.32697	1.61326
O	-1.44689	-0.34294	1.34156
H	-1.43356	-0.20405	0.35811
H	-0.89621	-1.15104	1.46351
O	0.23524	-2.10739	-1.68951
H	-0.42189	-1.37758	-1.74337
H	0.16043	-2.42268	-0.76365

G3 Energies at Each Temperature for (H2O)12 Dodecamer-168

```
Temperature=              200.000000 Pressure=                      1.000000
 E(ZPE)=                     0.293506 E(Thermal)=                    0.310062
 E(QCISD(T))=             -914.743196 E(Empiric)=                   -0.306528
 DE(Plus)=                  -0.133705 DE(2DF)=                      -0.885228
 E(Delta-G3)=               -0.954116 E(G3-Empiric)=                -0.306528
 G3(0 K)=                 -916.729267 G3 Energy=                  -916.712711
 G3 Enthalpy=             -916.712078 G3 Free Energy=             -916.763497

Temperature=              210.000000 Pressure=                      1.000000
 E(ZPE)=                     0.293506 E(Thermal)=                    0.311474
 E(QCISD(T))=             -914.743196 E(Empiric)=                   -0.306528
 DE(Plus)=                  -0.133705 DE(2DF)=                      -0.885228
 E(Delta-G3)=               -0.954116 E(G3-Empiric)=                -0.306528
 G3(0 K)=                 -916.729267 G3 Energy=                  -916.711299
 G3 Enthalpy=             -916.710634 G3 Free Energy=             -916.766104

Temperature=              216.650000 Pressure=                      1.000000
 E(ZPE)=                     0.293506 E(Thermal)=                    0.312434
 E(QCISD(T))=             -914.743196 E(Empiric)=                   -0.306528
 DE(Plus)=                  -0.133705 DE(2DF)=                      -0.885228
```

```
 E(Delta-G3)=                 -0.954116 E(G3-Empiric)=              -0.306528
 G3(0 K)=                   -916.729267 G3 Energy=                -916.710339
 G3 Enthalpy=               -916.709653 G3 Free Energy=           -916.767875

Temperature=                230.000000 Pressure=                     1.000000
 E(ZPE)=                       0.293506 E(Thermal)=                  0.314406
 E(QCISD(T))=               -914.743196 E(Empiric)=                 -0.306528
 DE(Plus)=                    -0.133705 DE(2DF)=                    -0.885228
 E(Delta-G3)=                 -0.954116 E(G3-Empiric)=              -0.306528
 G3(0 K)=                   -916.729267 G3 Energy=                -916.708366
 G3 Enthalpy=               -916.707638 G3 Free Energy=           -916.771524

Temperature=                240.000000 Pressure=                     1.000000
 E(ZPE)=                       0.293506 E(Thermal)=                  0.315923
 E(QCISD(T))=               -914.743196 E(Empiric)=                 -0.306528
 DE(Plus)=                    -0.133705 DE(2DF)=                    -0.885228
 E(Delta-G3)=                 -0.954116 E(G3-Empiric)=              -0.306528
 G3(0 K)=                   -916.729267 G3 Energy=                -916.706850
 G3 Enthalpy=               -916.706090 G3 Free Energy=           -916.774334

Temperature=                250.000000 Pressure=                     1.000000
 E(ZPE)=                       0.293506 E(Thermal)=                  0.317470
 E(QCISD(T))=               -914.743196 E(Empiric)=                 -0.306528
 DE(Plus)=                    -0.133705 DE(2DF)=                    -0.885228
 E(Delta-G3)=                 -0.954116 E(G3-Empiric)=              -0.306528
 G3(0 K)=                   -916.729267 G3 Energy=                -916.705303
 G3 Enthalpy=               -916.704511 G3 Free Energy=           -916.777210

Temperature=                260.000000 Pressure=                     1.000000
 E(ZPE)=                       0.293506 E(Thermal)=                  0.319047
 E(QCISD(T))=               -914.743196 E(Empiric)=                 -0.306528
 DE(Plus)=                    -0.133705 DE(2DF)=                    -0.885228
 E(Delta-G3)=                 -0.954116 E(G3-Empiric)=              -0.306528
 G3(0 K)=                   -916.729267 G3 Energy=                -916.703726
 G3 Enthalpy=               -916.702903 G3 Free Energy=           -916.780150

Temperature=                273.150000 Pressure=                     1.000000
 E(ZPE)=                       0.293506 E(Thermal)=                  0.321163
 E(QCISD(T))=               -914.743196 E(Empiric)=                 -0.306528
 DE(Plus)=                    -0.133705 DE(2DF)=                    -0.885228
 E(Delta-G3)=                 -0.954116 E(G3-Empiric)=              -0.306528
 G3(0 K)=                   -916.729267 G3 Energy=                -916.701610
 G3 Enthalpy=               -916.700745 G3 Free Energy=           -916.784110

Temperature=                298.150000 Pressure=                     1.000000
 E(ZPE)=                       0.293506 E(Thermal)=                  0.325311
 E(QCISD(T))=               -914.743196 E(Empiric)=                 -0.306528
 DE(Plus)=                    -0.133705 DE(2DF)=                    -0.885228
 E(Delta-G3)=                 -0.954116 E(G3-Empiric)=              -0.306528
 G3(0 K)=                   -916.729267 G3 Energy=                -916.697462
 G3 Enthalpy=               -916.696518 G3 Free Energy=           -916.791927

Temperature=                310.000000 Pressure=                     1.000000
 E(ZPE)=                       0.293506 E(Thermal)=                  0.327330
 E(QCISD(T))=               -914.743196 E(Empiric)=                 -0.306528
 DE(Plus)=                    -0.133705 DE(2DF)=                    -0.885228
```

E(Delta-G3)=	-0.954116	E(G3-Empiric)=	-0.306528
G3(0 K)=	-916.729267	G3 Energy=	-916.695443
G3 Enthalpy=	-916.694461	G3 Free Energy=	-916.795759

Temperature=	320.000000	Pressure=	1.000000
E(ZPE)=	0.293506	E(Thermal)=	0.329058
E(QCISD(T))=	-914.743196	E(Empiric)=	-0.306528
DE(Plus)=	-0.133705	DE(2DF)=	-0.885228
E(Delta-G3)=	-0.954116	E(G3-Empiric)=	-0.306528
G3(0 K)=	-916.729267	G3 Energy=	-916.693714
G3 Enthalpy=	-916.692701	G3 Free Energy=	-916.799055

Molecular Formula Structure
(H2O)13 Tredecamer

39

Energy: -620190.2774140

O	3.06211	-0.77142	-1.70173
H	3.77346	-1.15100	-2.24070
H	2.95881	-1.38950	-0.91666
O	2.63976	-2.26243	0.48080
H	1.66772	-2.36637	0.37993
H	2.71189	-1.62669	1.22582
O	-2.46745	-0.25929	-2.15411
H	-2.61844	-1.10033	-1.67340
H	-1.50900	-0.24554	-2.35514
O	-3.21594	1.45096	-0.21409
H	-2.92116	0.88114	-0.98186
H	-4.02847	1.88919	-0.51043
O	-0.32521	0.08221	2.08089
H	0.57640	0.09635	2.46873
H	-0.23014	-0.57075	1.34323
O	-0.03189	-1.73652	0.03465
H	0.06907	-1.12935	-0.74072
H	-0.90008	-2.18324	-0.08594
O	2.48833	-0.03844	2.26358
H	3.13243	0.14464	2.96526
H	2.74590	0.56738	1.50856
O	3.22280	1.41318	0.11916
H	3.36133	0.73727	-0.57765
H	2.46650	1.93712	-0.22470
O	-3.08091	-0.55584	1.77959
H	-3.25868	0.23791	1.22962
H	-2.21785	-0.36729	2.20383
O	0.39217	-0.03877	-2.09334
H	0.51956	0.88770	-1.77557
H	1.30822	-0.35942	-2.25505
O	-2.70262	-2.31038	-0.21122
H	-2.90196	-1.72539	0.57878
H	-3.24366	-3.10763	-0.10317
O	-0.90029	2.55515	0.93552
H	-0.67714	1.73540	1.43978
H	-1.77341	2.34822	0.53897
O	0.87193	2.46752	-1.00000
H	0.84323	3.29593	-1.50335
H	0.19269	2.56834	-0.25787

G3 Energies at Each Temperature for (H2O)13 Tredecamer

```
Temperature=              200.000000 Pressure=                   1.000000
 E(ZPE)=                     0.318956 E(Thermal)=                 0.336501
 E(QCISD(T))=             -990.980463 E(Empiric)=                -0.332072
 DE(Plus)=                  -0.142775 DE(2DF)=                   -0.959712
 E(Delta-G3)=               -1.032370 E(G3-Empiric)=             -0.332072
 G3(0 K)=                 -993.128437 G3 Energy=               -993.110892
 G3 Enthalpy=             -993.110258 G3 Free Energy=          -993.162943

Temperature=              210.000000 Pressure=                   1.000000
 E(ZPE)=                     0.318956 E(Thermal)=                 0.338022
 E(QCISD(T))=             -990.980463 E(Empiric)=                -0.332072
 DE(Plus)=                  -0.142775 DE(2DF)=                   -0.959712
 E(Delta-G3)=               -1.032370 E(G3-Empiric)=             -0.332072
 G3(0 K)=                 -993.128437 G3 Energy=               -993.109370
 G3 Enthalpy=             -993.108705 G3 Free Energy=          -993.165615

Temperature=              216.650000 Pressure=                   1.000000
 E(ZPE)=                     0.318956 E(Thermal)=                 0.339057
 E(QCISD(T))=             -990.980463 E(Empiric)=                -0.332072
 DE(Plus)=                  -0.142775 DE(2DF)=                   -0.959712
 E(Delta-G3)=               -1.032370 E(G3-Empiric)=             -0.332072
 G3(0 K)=                 -993.128437 G3 Energy=               -993.108336
 G3 Enthalpy=             -993.107650 G3 Free Energy=          -993.167434

Temperature=              230.000000 Pressure=                   1.000000
 E(ZPE)=                     0.318956 E(Thermal)=                 0.341185
 E(QCISD(T))=             -990.980463 E(Empiric)=                -0.332072
 DE(Plus)=                  -0.142775 DE(2DF)=                   -0.959712
 E(Delta-G3)=               -1.032370 E(G3-Empiric)=             -0.332072
 G3(0 K)=                 -993.128437 G3 Energy=               -993.106207
 G3 Enthalpy=             -993.105479 G3 Free Energy=          -993.171183

Temperature=              240.000000 Pressure=                   1.000000
 E(ZPE)=                     0.318956 E(Thermal)=                 0.342822
 E(QCISD(T))=             -990.980463 E(Empiric)=                -0.332072
 DE(Plus)=                  -0.142775 DE(2DF)=                   -0.959712
 E(Delta-G3)=               -1.032370 E(G3-Empiric)=             -0.332072
 G3(0 K)=                 -993.128437 G3 Energy=               -993.104570
 G3 Enthalpy=             -993.103810 G3 Free Energy=          -993.174076

Temperature=              250.000000 Pressure=                   1.000000
 E(ZPE)=                     0.318956 E(Thermal)=                 0.344494
 E(QCISD(T))=             -990.980463 E(Empiric)=                -0.332072
 DE(Plus)=                  -0.142775 DE(2DF)=                   -0.959712
 E(Delta-G3)=               -1.032370 E(G3-Empiric)=             -0.332072
 G3(0 K)=                 -993.128437 G3 Energy=               -993.102899
 G3 Enthalpy=             -993.102107 G3 Free Energy=          -993.177038

Temperature=              260.000000 Pressure=                   1.000000
 E(ZPE)=                     0.318956 E(Thermal)=                 0.346199
 E(QCISD(T))=             -990.980463 E(Empiric)=                -0.332072
 DE(Plus)=                  -0.142775 DE(2DF)=                   -0.959712
```

```
 E(Delta-G3)=                -1.032370 E(G3-Empiric)=              -0.332072
 G3(0 K)=                  -993.128437 G3 Energy=                -993.101194
 G3 Enthalpy=              -993.100370 G3 Free Energy=           -993.180070

Temperature=               273.150000 Pressure=                    1.000000
 E(ZPE)=                      0.318956 E(Thermal)=                  0.348488
 E(QCISD(T))=              -990.980463 E(Empiric)=                 -0.332072
 DE(Plus)=                   -0.142775 DE(2DF)=                    -0.959712
 E(Delta-G3)=                -1.032370 E(G3-Empiric)=              -0.332072
 G3(0 K)=                  -993.128437 G3 Energy=                -993.098904
 G3 Enthalpy=              -993.098039 G3 Free Energy=           -993.184158

Temperature=               298.150000 Pressure=                    1.000000
 E(ZPE)=                      0.318956 E(Thermal)=                  0.352979
 E(QCISD(T))=              -990.980463 E(Empiric)=                 -0.332072
 DE(Plus)=                   -0.142775 DE(2DF)=                    -0.959712
 E(Delta-G3)=                -1.032370 E(G3-Empiric)=              -0.332072
 G3(0 K)=                  -993.128437 G3 Energy=                -993.094413
 G3 Enthalpy=              -993.093469 G3 Free Energy=           -993.192242

Temperature=               310.000000 Pressure=                    1.000000
 E(ZPE)=                      0.318956 E(Thermal)=                  0.355166
 E(QCISD(T))=              -990.980463 E(Empiric)=                 -0.332072
 DE(Plus)=                   -0.142775 DE(2DF)=                    -0.959712
 E(Delta-G3)=                -1.032370 E(G3-Empiric)=              -0.332072
 G3(0 K)=                  -993.128437 G3 Energy=                -993.092226
 G3 Enthalpy=              -993.091244 G3 Free Energy=           -993.196211

Temperature=               320.000000 Pressure=                    1.000000
 E(ZPE)=                      0.318956 E(Thermal)=                  0.357040
 E(QCISD(T))=              -990.980463 E(Empiric)=                 -0.332072
 DE(Plus)=                   -0.142775 DE(2DF)=                    -0.959712
 E(Delta-G3)=                -1.032370 E(G3-Empiric)=              -0.332072
 G3(0 K)=                  -993.128437 G3 Energy=                -993.090353
 G3 Enthalpy=              -993.089339 G3 Free Energy=           -993.199628
```

Molecular Formula Structure
(H2O)14 Quattuordecamer

```
42
        Energy: -667898.0263208
O          2.22488       -2.22752       -1.45428
H          2.68564       -1.33697       -1.51588
H          2.70409       -2.81375       -2.06003
O         -0.40139       -1.35099       -1.36491
H         -0.37642       -1.24428       -0.38275
H          0.44267       -1.80962       -1.57712
O          2.19424       -2.21496        1.38534
H          2.24763       -2.43356        0.42999
H          1.23899       -2.04210        1.53608
O         -3.18324       -1.55523        1.31896
H         -3.24030       -1.60847        0.31589
H         -3.82883       -2.19561        1.65590
O         -0.43788       -1.26179        1.41791
H         -0.43080       -0.34941        1.79538
H         -1.36113       -1.57581        1.55228
```

```
O          3.46137        0.13824        1.42346
H          4.23070        0.05656        2.00791
H          2.98187       -0.74209        1.48345
O          3.45931        0.13815       -1.39723
H          2.84818        0.89449       -1.54417
H          3.67165        0.19852       -0.44094
O          1.63471        2.30064       -1.44500
H          0.75507        1.95394       -1.79144
H          1.85951        3.04949       -2.01874
O         -0.56707        1.21852       -2.42091
H         -0.47188        0.27574       -2.14850
H         -1.46478        1.45338       -2.10427
O         -3.19045        1.31954       -1.36633
H         -3.92142        1.87984       -1.66924
H         -3.19238        1.38985       -0.36720
O         -3.18100       -1.51780       -1.34593
H         -3.31028       -0.56165       -1.53055
H         -2.23283       -1.66647       -1.55791
O          1.70529        2.35633        1.35926
H          1.66247        2.48234        0.38500
H          2.36408        1.63712        1.47600
O         -0.54510        1.35742        2.31825
H         -0.48968        1.53448        3.27050
H          0.27212        1.79859        1.92365
O         -3.19456        1.28554        1.31302
H         -3.33239        0.32916        1.48198
H         -2.29065        1.46018        1.65315
```

G3 Energies at Each Temperature for (H2O)14 Quattuordecamer

```
Temperature=              200.000000 Pressure=                    1.000000
 E(ZPE)=                     0.343451 E(Thermal)=                  0.362478
 E(QCISD(T))=            -1067.209761 E(Empiric)=                 -0.357616
 DE(Plus)=                  -0.156620 DE(2DF)=                    -1.032987
 E(Delta-G3)=               -1.111757 E(G3-Empiric)=              -0.357616
 G3(0 K)=                -1069.525289 G3 Energy=               -1069.506262
 G3 Enthalpy=            -1069.505629 G3 Free Energy=          -1069.561828

Temperature=              210.000000 Pressure=                    1.000000
 E(ZPE)=                     0.343451 E(Thermal)=                  0.364121
 E(QCISD(T))=            -1067.209761 E(Empiric)=                 -0.357616
 DE(Plus)=                  -0.156620 DE(2DF)=                    -1.032987
 E(Delta-G3)=               -1.111757 E(G3-Empiric)=              -0.357616
 G3(0 K)=                -1069.525289 G3 Energy=               -1069.504620
 G3 Enthalpy=            -1069.503955 G3 Free Energy=          -1069.564679

Temperature=              216.650000 Pressure=                    1.000000
 E(ZPE)=                     0.343451 E(Thermal)=                  0.365237
 E(QCISD(T))=            -1067.209761 E(Empiric)=                 -0.357616
 DE(Plus)=                  -0.156620 DE(2DF)=                    -1.032987
 E(Delta-G3)=               -1.111757 E(G3-Empiric)=              -0.357616
 G3(0 K)=                -1069.525289 G3 Energy=               -1069.503503
 G3 Enthalpy=            -1069.502817 G3 Free Energy=          -1069.566620

Temperature=              230.000000 Pressure=                    1.000000
```

```
 E(ZPE)=                     0.343451 E(Thermal)=                    0.367534
 E(QCISD(T))=            -1067.209761 E(Empiric)=                   -0.357616
 DE(Plus)=                  -0.156620 DE(2DF)=                      -1.032987
 E(Delta-G3)=               -1.111757 E(G3-Empiric)=                -0.357616
 G3(0 K)=                -1069.525289 G3 Energy=                 -1069.501206
 G3 Enthalpy=            -1069.500478 G3 Free Energy=            -1069.570622

Temperature=               240.000000 Pressure=                     1.000000
 E(ZPE)=                     0.343451 E(Thermal)=                    0.369301
 E(QCISD(T))=            -1067.209761 E(Empiric)=                   -0.357616
 DE(Plus)=                  -0.156620 DE(2DF)=                      -1.032987
 E(Delta-G3)=               -1.111757 E(G3-Empiric)=                -0.357616
 G3(0 K)=                -1069.525289 G3 Energy=                 -1069.499440
 G3 Enthalpy=            -1069.498680 G3 Free Energy=            -1069.573710

Temperature=               250.000000 Pressure=                     1.000000
 E(ZPE)=                     0.343451 E(Thermal)=                    0.371105
 E(QCISD(T))=            -1067.209761 E(Empiric)=                   -0.357616
 DE(Plus)=                  -0.156620 DE(2DF)=                      -1.032987
 E(Delta-G3)=               -1.111757 E(G3-Empiric)=                -0.357616
 G3(0 K)=                -1069.525289 G3 Energy=                 -1069.497635
 G3 Enthalpy=            -1069.496844 G3 Free Energy=            -1069.576874

Temperature=               260.000000 Pressure=                     1.000000
 E(ZPE)=                     0.343451 E(Thermal)=                    0.372945
 E(QCISD(T))=            -1067.209761 E(Empiric)=                   -0.357616
 DE(Plus)=                  -0.156620 DE(2DF)=                      -1.032987
 E(Delta-G3)=               -1.111757 E(G3-Empiric)=                -0.357616
 G3(0 K)=                -1069.525289 G3 Energy=                 -1069.495796
 G3 Enthalpy=            -1069.494972 G3 Free Energy=            -1069.580112

Temperature=               273.150000 Pressure=                     1.000000
 E(ZPE)=                     0.343451 E(Thermal)=                    0.375416
 E(QCISD(T))=            -1067.209761 E(Empiric)=                   -0.357616
 DE(Plus)=                  -0.156620 DE(2DF)=                      -1.032987
 E(Delta-G3)=               -1.111757 E(G3-Empiric)=                -0.357616
 G3(0 K)=                -1069.525289 G3 Energy=                 -1069.493325
 G3 Enthalpy=            -1069.492460 G3 Free Energy=            -1069.584480

Temperature=               298.150000 Pressure=                     1.000000
 E(ZPE)=                     0.343451 E(Thermal)=                    0.380262
 E(QCISD(T))=            -1067.209761 E(Empiric)=                   -0.357616
 DE(Plus)=                  -0.156620 DE(2DF)=                      -1.032987
 E(Delta-G3)=               -1.111757 E(G3-Empiric)=                -0.357616
 G3(0 K)=                -1069.525289 G3 Energy=                 -1069.488478
 G3 Enthalpy=            -1069.487534 G3 Free Energy=            -1069.593120

Temperature=               310.000000 Pressure=                     1.000000
 E(ZPE)=                     0.343451 E(Thermal)=                    0.382623
 E(QCISD(T))=            -1067.209761 E(Empiric)=                   -0.357616
 DE(Plus)=                  -0.156620 DE(2DF)=                      -1.032987
 E(Delta-G3)=               -1.111757 E(G3-Empiric)=                -0.357616
 G3(0 K)=                -1069.525289 G3 Energy=                 -1069.486118
 G3 Enthalpy=            -1069.485136 G3 Free Energy=            -1069.597363
```

```
Temperature=              320.000000 Pressure=                 1.000000
 E(ZPE)=                     0.343451 E(Thermal)=               0.384644
 E(QCISD(T))=            -1067.209761 E(Empiric)=              -0.357616
 DE(Plus)=                  -0.156620 DE(2DF)=                 -1.032987
 E(Delta-G3)=               -1.111757 E(G3-Empiric)=           -0.357616
 G3(0 K)=                -1069.525289 G3 Energy=            -1069.484096
 G3 Enthalpy=            -1069.483083 G3 Free Energy=       -1069.601016
```

*Corresponding author.

ali.afzalifar@aalto.fi

REFRENCES

(S1) Wölk, J.; Strey, R.; Heath, C. H.; Wyslouzil, B. E. Empirical Function for Homogeneous Water Nucleation Rates. J. Chem. Phys. 2002, 117 (10), 4954–4960.
(S2) Wölk, J.; Strey, R. Homogeneous Nucleation of H2O and D2O in Comparison: The Isotope Effect. J. Phys. Chem. B 2001, 105 (47), 11683–11701.
(S3) Manka, A. A.; Brus, D.; Hyvärinen, A.-P.; Lihavainen, H.; Wölk, J.; Strey, R. Homogeneous Water Nucleation in a Laminar Flow Diffusion Chamber. J. Chem. Phys. 2010, 132 (24), 244505.
(S4) Brus, D.; Zdímal, V.; Smolík, J. Homogeneous Nucleation Rate Measurements in Supersaturated Water Vapor. J. Chem. Phys. 2008, 129 (17), 174501.
(S5) Brus, D.; Zdímal, V.; Uchtmann, H. Homogeneous Nucleation Rate Measurements in Supersaturated Water Vapor II. J. Chem. Phys. 2009, 131 (7), 074507.
(S6) Miller, R. C.; Anderson, R. J.; Kassner, J. L.; Hagen, D. E. Homogeneous Nucleation Rate Measurements for Water over a Wide Range of Temperature and Nucleation Rate. J. Chem. Phys. 1983, 78 (6), 3204–3211.
(S7) Luijten, C. C. M.; Bosschaart, K. J.; van Dongen, M. E. H. High Pressure Nucleation in Water/Nitrogen Systems. J. Chem. Phys. 1997, 106 (19), 8116–8123.
(S8) Holten, V.; Labetski, D. G.; van Dongen, M. E. H. Homogeneous Nucleation of Water between 200 and 240 K: New Wave Tube Data and Estimation of the Tolman Length. J. Chem. Phys. 2005, 123 (10), 104505.
(S9) Kim, Y. J.; Wyslouzil, B. E.; Wilemski, G.; Wölk, J.; Strey, R. Isothermal Nucleation Rates in Supersonic Nozzles and the Properties of Small Water Clusters. J. Phys. Chem. A 2004, 108 (20), 4365–4377.
(S10) Mikheev, V. B.; Irving, P. M.; Laulainen, N. S.; Barlow, S. E.; Pervukhin, V. V. Laboratory Measurement of Water Nucleation Using a Laminar Flow Tube Reactor. J. Chem. Phys. 2002, 116 (24), 10772–10786.
(S11) Saltz, D. Using the Noninteracting Cluster Theory to Predict the Properties of Real Vapor. J. Chem. Phys. 1994, 101 (7), 6038–6051.
(S12) International Association For The Properties of Water and Steam. Revised Release on the IAPWS Formulation 1995 for the Thermodynamic Properties of Ordinary Water Substance for General and Scientific Use. 2018.
(S13) Hill, P. G.; MacMillan, R. D. C. Virial Equations for Light and Heavy Water. Ind. Eng. Chem. Res. 1988, 27 (5), 874–882.
(S14) Haar, L. Nbs/Nrc Steam Tables; CRC Press, 1984.
